# Phytotoxicity Study of (Amino)imidazo[1,2-*a*]pyridine Derivatives Toward the Control of *Bidens pilosa*, *Urochloa decumbens*, and *Panicum maximum* Weeds

**DOI:** 10.1021/acs.jafc.4c09477

**Published:** 2024-12-28

**Authors:** Luan A. Martinho, Daniel M. de Lima, Victor H. J. G. Praciano, Sarah Christina C. Oliveira, Carlos Kleber Z Andrade

**Affiliations:** † Instituto de Química, Laboratório de Química Metodológica e Orgânica Sintética (LaQMOS), Universidade de Brasília, 70904-970 Brasília, DF, Brazil; ‡ Instituto de Ciências Biológicas, Departamento de Botânica, Laboratório de Alelopatia Alfredo Gui Ferreira, Universidade de Brasília, 70910-900 Brasília, DF, Brazil

**Keywords:** imidazo[1,2-*a*]pyridine, GBB reaction, tetrazoles, phytotoxicity, weed control

## Abstract

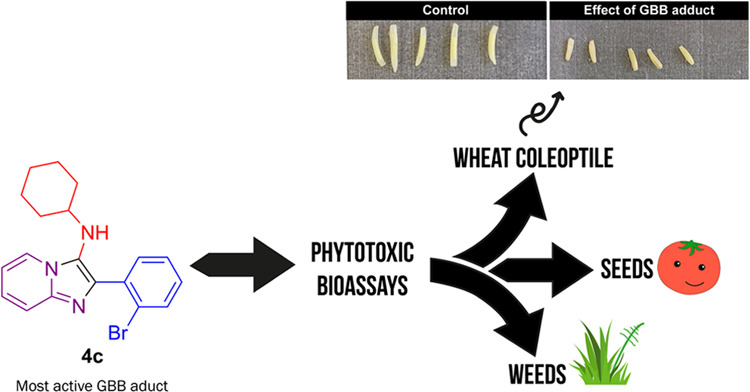

In this work, several imidazo[1,2-*a*]pyridines were synthesized through the Groebke–Blackburn–Bienaymé three-component reaction (GBB-3CR), and their phytotoxicity was evaluated *in vitro* by the influence on the growth of wheat coleoptiles and three important agricultural seeds (*Allium cepa*, *Lactuca sativa*, and *Solanum lycopersicum*) at test concentrations of 1000, 300, 100, 30, and 10 μM. A structure–activity relationship was established, showing the importance of halogen groups at the *ortho* position of the attached aromatic ring and the presence of a cyclohexylamine group for greater activity. Post-modification of some GBB-3CR adducts was carried out, leading to imidazo[1,2-*a*]pyridine-tetrazole hybrids, which were also evaluated in these bioassays. The phytotoxicity on seed germination and growth bioassays demonstrated that *A. cepa* was the most susceptible seed, and the most affected parameters were the root and shoot lengths. The most active compound was also evaluated against *Bidens pilosa*, *Urochloa decumbens*, and *Panicum maximum* weeds under hydroponic conditions to assess its phytotoxic potential at a more advanced level of bioassays. Promising results were also achieved, in which the most affected growth factor by inhibition was the root growth, and a stimulus to shoot growth was noted, making it a promising hit in the search for new herbicides.

## 1 Introduction

The escalation of weed resistance to current techniques aimed at enhancing food production has emerged as a global challenge over the past decade.^1^ The need to meet food demand becomes more urgent in view of the fact that the Zero Hunger is the second objective of the United Nations Sustainable Development Goals, to be achieved by 2030.^2^ The indiscriminate use of chemicals for herbicide control over the years has not only given rise to herbicide-resistant weeds but has also engendered environmental issues, including harm to nontarget organisms and the contamination of soil, water, food, and human health.^3^ Therefore, the search for new agricultural interventions and technologies, which prioritize fastness, efficacy, and cost-effectiveness in weed management practices, has become of paramount importance.^4^

A tool employed to identify novel compounds with a potential phytotoxic activity involves the utilization of allelopathy, which is characterized as the manifestation of defense and signal substance effects exerted by a plant species on the germination, growth, and/or development of other plants, irrespective of whether they belong to the same or different species.^5^ This influence is mediated through the release of secondary metabolites (allelochemicals), such as terpenoids, volatile organic compounds, glycosides, alkaloids, phenolic compounds, and organic acids into the shared environment.^6^

Additionally, another way is an indirect intervention using chemicals that are artificially synthesized, specifically via synthetic organic chemistry.^7^ This synthetic methodology offers a pathway to enable the targeted product on a large scale with both efficiency and economic viability.^8−10^

**Figure 1:**
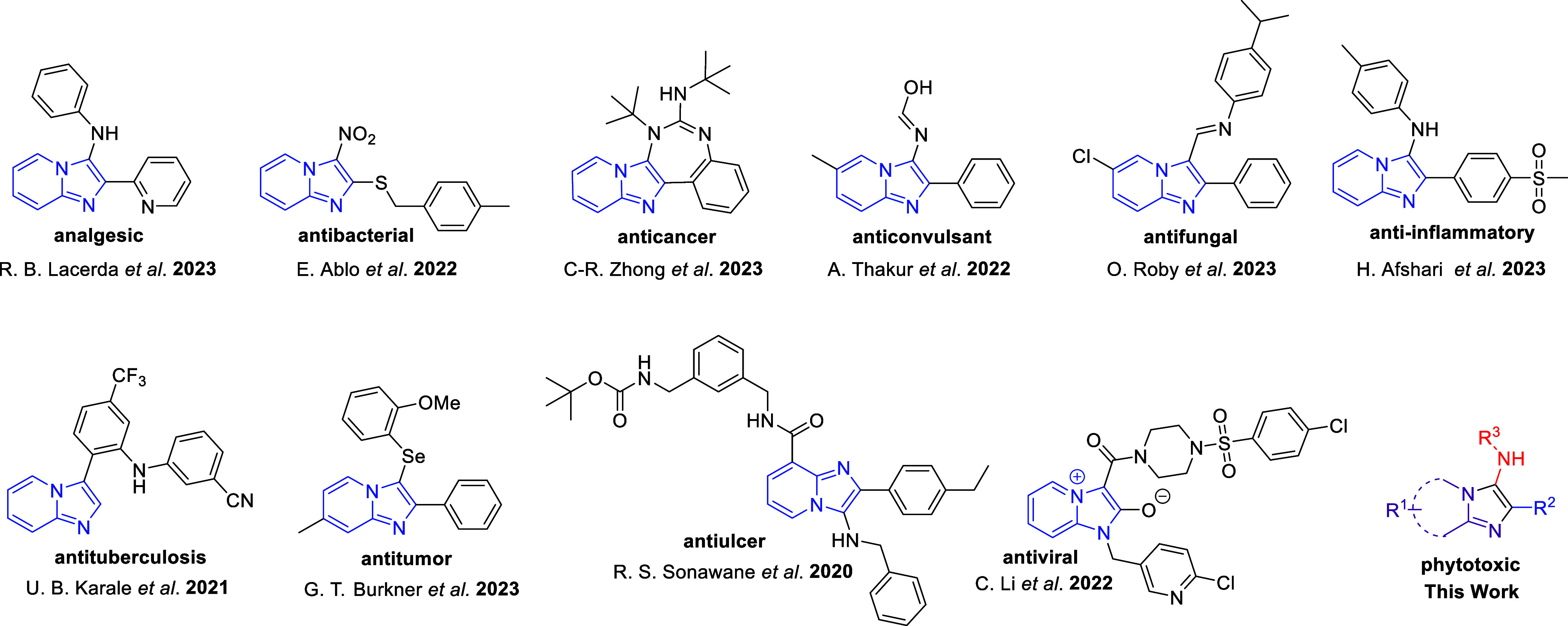
Selected biologically and medicinally active molecules bearing the imidazo[1,2-*a*]pyridine moiety.

In this respect, during the course of our studies on multicomponent reactions (MCRs), we turned our attention to imidazo[1,2-*a*]pyridine derivatives, obtained via the Groebke–Blackburn–Bienaymé three-component reaction (GBB-3CR).^11−13^ This reaction has found diverse applications in combinatorial and medicinal chemistry, with its products being of great use in the discovery of new compounds with potentially significant biological activities,^14^ such as analgesic,^15^ antibacterial,^16^ anticancer,^17^ anticonvulsant,^18^ antifungal,^19^ anti-inflammatory,^20^ antituberculosis,^21^ antitumor,^22^ antiulcer,^23^ and antiviral^24^ (Figure 1). In addition, it is present in several commercial drugs such as Alpidem, Miroprofen, Necopidem, Saripidem, Zolimidine, and Zolpidem.^25^ However, its potential phytotoxic activity is still neglected. To the best of our knowledge, there are only two reports in the literature on the qualitative herbicidal activity of sulfonylurea and phenoxypropionic acid derivatives of imidazo[1,2-*a*]pyridines, which date back to the early 1990s.^26,27^

Herein, we report an extensive phytotoxicity profile of GBB-3CR adducts by using easily accessible starting materials. Promising results were found for some of these compounds against the etiolated wheat coleoptile bioassay and against three important agricultural seeds (*Allium cepa*, *Lactuca sativa*, and *Solanum lycopersicum*). The most active compound was also evaluated against the weeds *Bidens pilosa*, *Urochloa decumbens*, and *Panicum maximum* under hydroponic conditions. Furthermore, the synthesis and initial phytotoxic studies were performed with imidazo[1,2-*a*]pyridine-tetrazole hybrids.

## 2 Materials and Methods

### 2.1 General

All microwave-mediated reactions were performed on a Biotage Initiator^+^ microwave reactor using a sealed vessel with simultaneous cooling, media stirring, and temperature detection via an internal fiber optic probe. The products were purified by column chromatography performed on silica gel (Supelco, pore size 60 Å, 230–400 mesh particle size, 40–63 μm particle size), and mixtures of hexane/ethyl acetate were used as eluents. Thin-layer chromatography was used on the ultraviolet (UV) fluorescent silica gel Merck 60 F254 plates and visualized by treatment with a 10% solution of phosphomolybdic acid (PMA) in ethanol, followed by heating. The Fourier transform infrared (FT-IR) spectra were performed on a Varian 640 spectrometer with TA DLaTGS as the detector in the infrared region (4000–600 cm^–1^) in attenuated total reflection ATR mode (4000–400 cm^–1^) or in KBr mode. Nuclear magnetic resonance (NMR) spectra were obtained on a 600 MHz spectrometer (Bruker Ascend 600). Chemical shifts are given in ppm concerning residual ^1^H signals of CDCl_3_ (δ 7.26) or DMSO-*d*_6_ (δ 2.50), and ^13^C signals are referenced to the solvent signal (δ 77.2) or (δ 39.5). Exact masses were measured on a Triple Tof 5600 Sciex by flow injection analysis using an Eksigent UltraLC 100 Sciex chromatograph set to a flow rate of 0.3 mL/min. A DuoSpray Ion Source (ESI) was used, and the MS spectra were acquired in positive mode, employing external calibration, in the range of 50–1000 Da and 0.1% (v/v) formic acid in acetonitrile as solvent. The melting points were measured with a capillary in LOGEN Scientific equipment (LS III Plus) and were not corrected. Unless otherwise stated, all reagents and solvents were purchased from Sigma-Aldrich (Merck) and used without further purification.

### 2.2 Synthesis of Imidazo[1,2-*a*]pyridine Derivatives via GBB Three-Component Reaction

A Biotage microwave reaction vial of 0.5–2.0 mL containing a mixture of 2-aminopyridine (0.50 mmol), aldehyde (0.50 mmol), isocyanide (0.50 mmol), and phosphotungstic acid hydrate HPW (0.01 mmol, 2 mol %) in EtOH (0.5 mL) was introduced into the cavity of a microwave reactor Biotage Initiator^+^ and heated at 120 °C for 30 min under magnetic stirring. The reaction mixture was cooled to room temperature, and reagents’ consumption was confirmed by thin-layer chromatography (TLC) analysis (mixture of ethyl acetate/hexane). The reaction mixture was concentrated under vacuum, and the crude product was purified by silica gel column chromatography. For full details of the synthesis and characterization of imidazo[1,2-*a*]pyridines (**4a**-**w, 4z, 4bb**-**pp**) studied in this work, see the article previously reported by our research group.^28^ The previously unpublished compounds **4x**, **4y**, **4aa**, and **4qq**-**vv** were synthesized following the standard methodology.

### 2.3 Synthesis of Imidazo[1,2-*a*]pyridine-Tetrazole Hybrids via [3 + 2] Cycloaddition Reaction

In a 2.0–5.0 mL microwave vial, ZnCl_2_ (0.50 mmol), the GBB reaction product (0.50 mmol), NaN_3_ (1.00 mmol), and EtOH (1 mL) were added. The mixture was heated in a microwave at 130 °C for 2 h. After the reaction was complete, the mixture was allowed to cool to room temperature. Then, approximately 3–4 mL of cold water was added to the reaction mixture, followed by 1–2 mL of 2.5 M HCl until a solid precipitated. The resulting solid was collected by vacuum filtration and washed with cold ethanol or water. The expected products were isolated without further purification.

### 2.4 Wheat Coleoptile Bioassay

The wheat coleoptile bioassay was performed according to the procedure previously described by Da Silva et al.^29^ The wheat seeds (*Triticum aestivumL*. cv. BRS394) were supplied by Cooperativa Serra dos Cristais (Crystalina-GO, Brazil). Approximately 100 wheat seeds were added to 15 cm diameter Petri dishes (11 × 11 × 3.5 cm^3^) lined with one sheet of qualitative filter paper and moistened with 15 mL of distilled water. The dishes were placed in a germination chamber for 3 days at 25 ± 1 °C in the dark. Following this period, the etiolated seedling coleoptiles were cut into 4 mm pieces to remove the shoots that had grown, the roots, and the caryopsis with the support of a Van der Veij guillotine under green safelight for use in a bioassay to prevent further growth. All of the samples were dissolved in 0.5% (5 μL/mL) v/v of dimethyl sulfoxide (DMSO), and the compounds were tested at concentrations of 1000, 300, 100, 30, and 10 μM. The GBB adducts were presolubilized in DMSO and diluted with a buffered solution (pH 5.6) containing citric acid monohydrate (1.05 g L^–1^), potassium hydrogen phosphate trihydrate (2.9 g L^–1^), and 2% sucrose. Each treatment was tested in triplicate by adding 2 mL of each solution and five fragments of wheat coleoptile to a glass test tube (16 × 100 mm^2^, 10 mL). The control was a pure buffer solution containing 5 μL/mL of DMSO (negative control). The DCMU herbicide (Diuron) was used as a positive control. The test tubes were capped and kept in a growth chamber at 25 ± 1 °C in the dark and with constant rotation in a drum tube rotor (0.25 rpm). After 24 h, the coleoptiles were removed from the tubes, and the increase in the length of the coleoptile was evaluated. The coleoptiles were placed on a black background sheet, divided into rectangular cells (40 × 20 mm^2^), and classified according to their dilution and replication. The cells were then photographed, and the photos were digitized using the ImageJ program to measure the coleoptiles.^30^ Data were analyzed statistically using Welch’s test. The results are presented as percentages relative to the control: Zero represents the control, positive values represent stimulation of growth, and negative values indicate inhibition of growth, using Excel (Microsoft Corporation, 2024) and OriginPro (version 2024) software. The calculation of IC_50_ values was determined by nonlinear regression, using GraphPadPrism 9 software package.

### 2.5 Seed Bioassay

The phytotoxicity bioassay was performed according to the procedure previously described by Da Silva et al.^29^ The weed species used as target plants in this bioassay included the monocotyledon Creole Onion (*A. cepa*), dicotyledon Lettuce Hanson (*L. sativa*), and Tomato San Marzano (*S. lycopersicum*). The seeds for all three species were purchased from Casa Agropecuária (Brasília-DF, Brazil) under the trade name of Feltrin or Isla Seeds. Bioassays were conducted in Petri dishes (50 mm diameter) with one sheet of qualitative filter paper. Germination and growth were conducted in aqueous solutions at a controlled pH of 6.0 using 10^–2^ M 2-[*N*-morpholino]ethanesulfonic acid (MES) and 1.0 M NaOH. The GBB adducts to be tested were dissolved in a buffer at test concentrations of 1000, 300, 100, 30, and 10 μM. Before evaluating the standard dilutions, 0.5% v/v DMSO was added to enhance the solubility of the products. Four replicates containing 20 seeds each were used. Treatment, control, or internal reference solution (1 mL) was added to each Petri dish. The DCMU herbicide (Diuron) was used as a positive control at the same concentrations, and the buffer solution was used as a negative control. The Petri dishes were sealed with a PVC film to ensure closed-system models. Seeds were incubated at 25 °C in a controlled environment growth chamber in the absence of light. The bioassays took 5 days for *L. sativa* species or 6 days for *A. cepa* and *S. lycopersicum* species. After growth, the plants were frozen at −10 °C for 24 h to prevent further growth during the measurement process. The germinated seeds were placed on a white plastic film background sheet and then photographed, and the photos were digitized using the ImageJ program to measure the parameters evaluated, including germination rate, root length, and shoot length.^30^ Data were statistically analyzed using Welch’s test. Germination percentage, root length, and shoot length are shown as the percent difference from the control by using Excel (Microsoft Corporation, 2024) and OriginPro (version 2024) softwares. The calculation of IC_50_ values was determined by nonlinear regression, using the GraphPadPrism 9 software package.

### 2.6 Hydroponic Bioassay

The hydroponic bioassay was performed according to the procedure previously described by Da Silva et al.^29^ The weed species used as target plants in this bioassay included the asteraceae *B. pilosa* and guinea grasses of *U. decumbens* and *P. maximum*. The seeds for all three species were purchased from Agrocosmus Agropecuária (Alegrete-RS, Brazil). Germination and growth were conducted in a culture medium in a pot with a volume of 3 L filled with a Bioplant Garden Premium substrate for 30 days for *B. pilosa* and 21 days for *Brachiaria decumbens* and *P. maximum*. After their emergence, the samples were transferred to the receptacles containing the solutions of the evaluated compounds at appropriate concentrations. The pure products were predissolved in DMSO 0.5% v/v and diluted in the nutrient solution of Murashige and Skoog basal medium at 1/3 ionic strength and subsequently dissolved at concentrations of 1000, 300, 100, 30, and 10 μM. The DCMU herbicide (Diuron) was used as a positive control at the same concentrations. As a negative control, Murashige and Skoog basal medium at 1/3 ionic strength plus DMSO 0.5% v/v was used. Four replicates were performed for each concentration. Glass containers with a diameter of 6.0 cm and height of 10.0 cm were used in the experiments, and these were wrapped externally with aluminum foil. As a substrate, 75 g of glass spheres with a diameter of 3 mm were used, and 20 mL of solution were added to each container. The ends of the containers were capped with a PVC film to maintain moisture. Each container with a seedling represented one sample plot. The experiment was conducted for 21 or 30 days in an air-conditioned room regulated at 25 °C with a photoperiod of 12 h. 21 or 30 days after transferring the seedlings to the containers, the trials were ended. The seedlings were then stretched on a black plate to measure the length of the aerial part and the roots and then photographed. The photos were digitized using the ImageJ program to measure the parameters evaluated, including the root length and shoot length.^30^ Data were statistically analyzed using Welch’s test. Root and shoot lengths are shown as the percent difference from the control using Excel (Microsoft Corporation, 2024) and OriginPro (version 2024) softwares. The calculation of IC_50_ values was determined by nonlinear regression, using the GraphPadPrism 9 software package.

## 3 Results and Discussion

### 3.1 Synthesis

#### 3.1.1 Synthesis of Imidazo[1,2-*a*]pyridine Derivatives via GBB-3CR

**Scheme 1:**
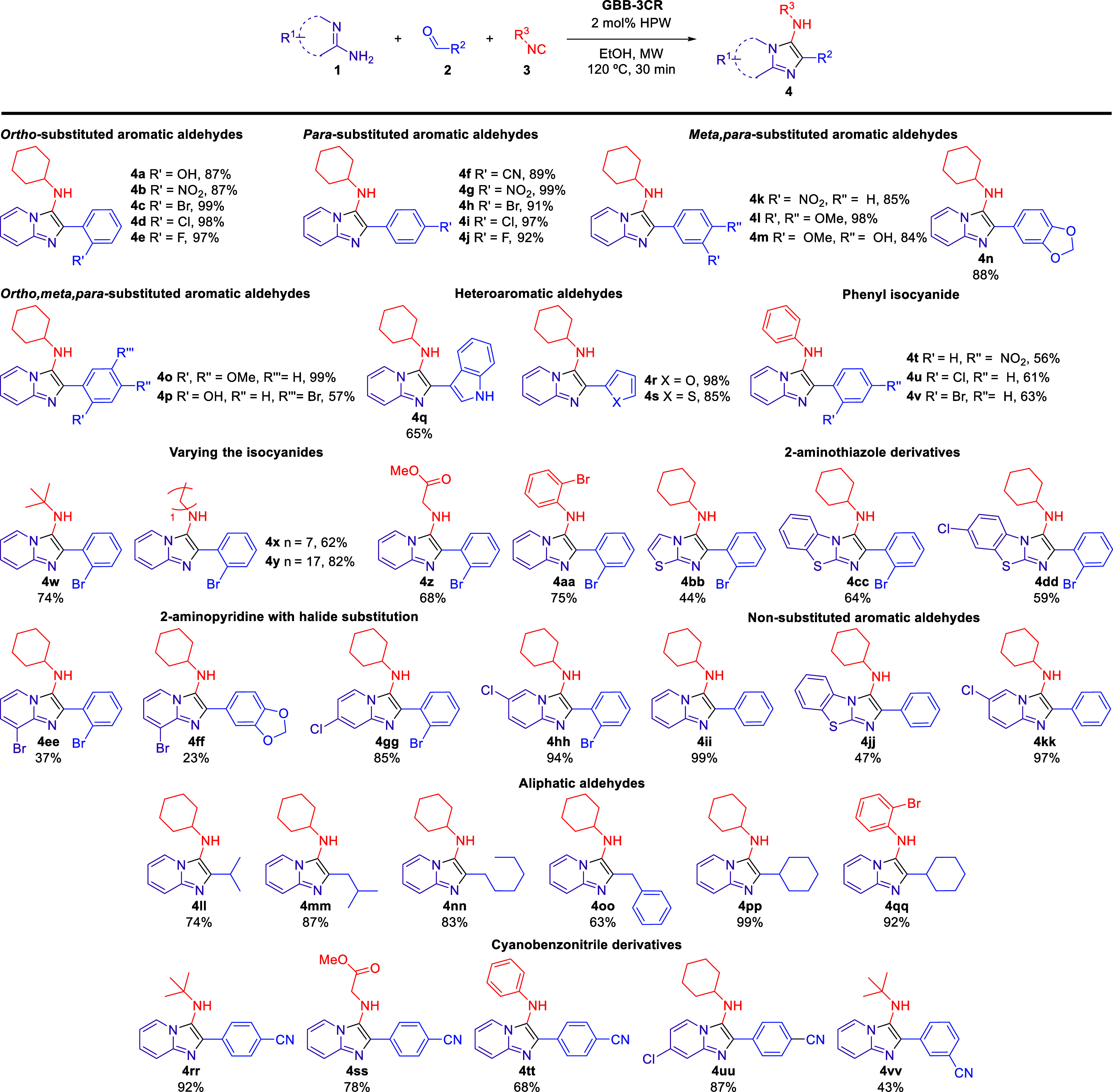
Substrate Scope of the HPW-Catalyzed GBB Reactions Using a Range of Aromatic/Heteroaromatic Aldehydes Reaction conditions: 2-aminopyridine/2-aminothiazole (0.50 mmol), aldehyde (0.50 mmol), isocyanide (0.50 mmol), and HPW (0.01 mmol, 2 mol %) in EtOH (0.50 mL), under microwave (MW) heating. The yields are isolated yields after purification, and the structures were confirmed by FT-IR, NMR, and HRMS.

The synthesis of imidazo[1,2-*a*]pyridine derivatives was accomplished using the GBB-3CR method with a range of 2-aminopyridines/2-aminothiazole (**1**), aldehydes (**2**), and isocyanides (**3**), leveraging a recent approach developed by our research group.^28^ This method employed phosphotungstic acid, H_3_PW_12_O_40_ (HPW), as a highly efficient green catalyst, in ethanol, with microwave (MW) heating, as illustrated in Scheme 1. This protocol was very efficient for a range of aliphatic and (hetero)aromatic aldehydes, several isocyanides, substituted 2-aminopyridines, and 2-aminothiazole derivatives. The imidazo[1,2-*a*]pyridines **4a**-**w, 4z**, and **4bb-pp** are known compounds already reported by our research group.^28^ The previously unpublished compounds **4x**, **4y**, **4aa**, and **4qq**-**vv** were synthesized to verify the influence of 2-bromobenzene synthon and different substitutes for cyanobenzonitrile derivatives in a bioassay phytotoxicity profile.

#### 3.1.2 Synthesis of Imidazo[1,2-*a*]pyridine-tetrazole Hybrids via [3 + 2] Cycloaddition Reaction

**Scheme 2:**
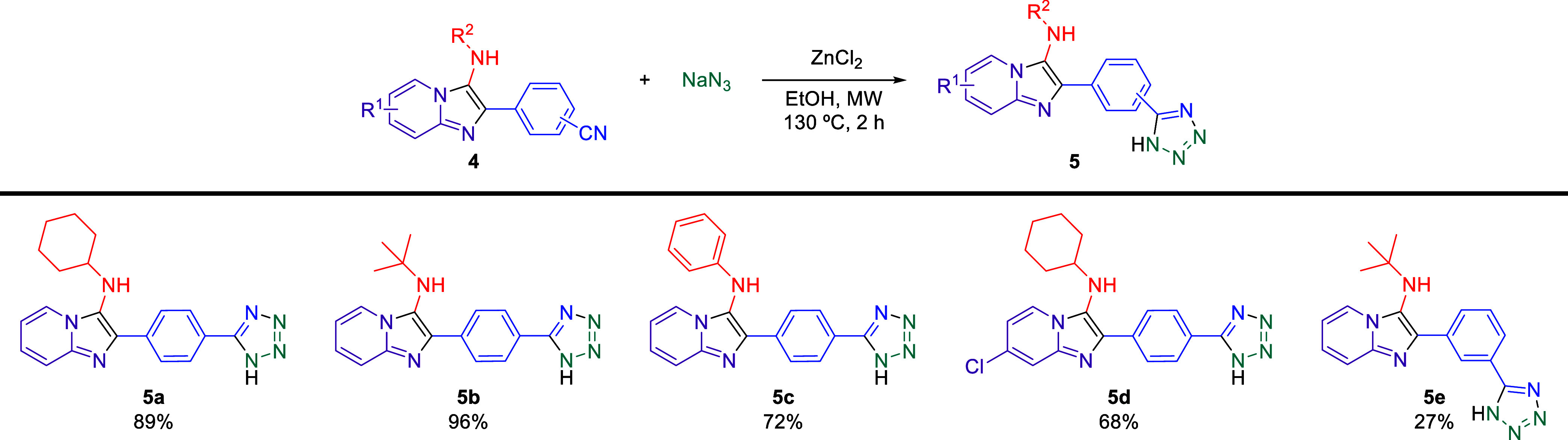
Synthesis of Imidazo[1,2-*a*]pyridine-tetrazole Hybrids by ZnCl_2_-Mediated [3 + 2] Cycloaddition Reactions Reaction conditions: GBB adducts **4rr-4vv** (0.50 mmol), NaN_3_ (1.00 mmol), and ZnCl_2_ (0.50 mmol) in EtOH (1 mL), under MW heating. The yields are isolated yields after purification, and the structures were confirmed by FT-IR, NMR, and HRMs.

Tetrazoles are aromatic heterocyclic compounds with various applications,^31^ including antibacterial,^32^ herbicidal,^33^ anti-inflammatory,^34^ and antihypertensive activities, as seen in drugs like losartan and valsartan.^35^ The synthesis of these compounds often involves the [3 + 2] cycloaddition reaction.^36^ However, attempts to replicate existing methodologies from the literature resulted in impure products with low yields. Consequently, a new methodology was developed herein by using microwave heating. After several trials, the following reaction conditions were identified as ideal for the formation of monosubstituted tetrazoles with NaN_3_ under microwave heating and using ZnCl_2_ as an additive to obtain the corresponding tetrazole hybrids **5a**–**e** as shown in Scheme 2.

### 3.2 Phytotoxicity Bioassay

#### 3.2.1 Etiolated Wheat Coleoptile Bioassay

**Figure 2:**
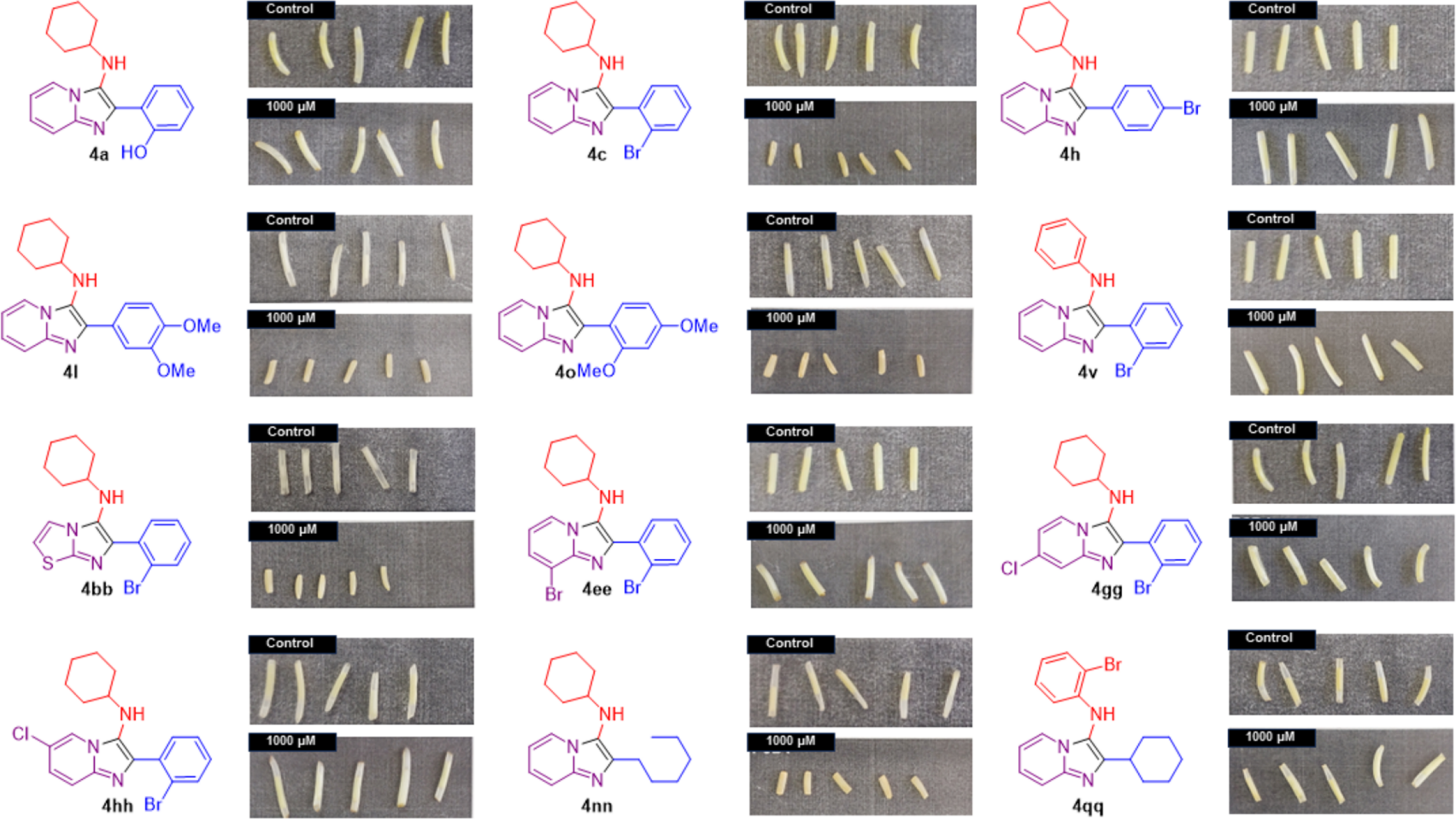
Effect of selected imidazo[1,2-*a*]pyridine derivatives on wheat coleoptile elongation.

**Figure 3:**
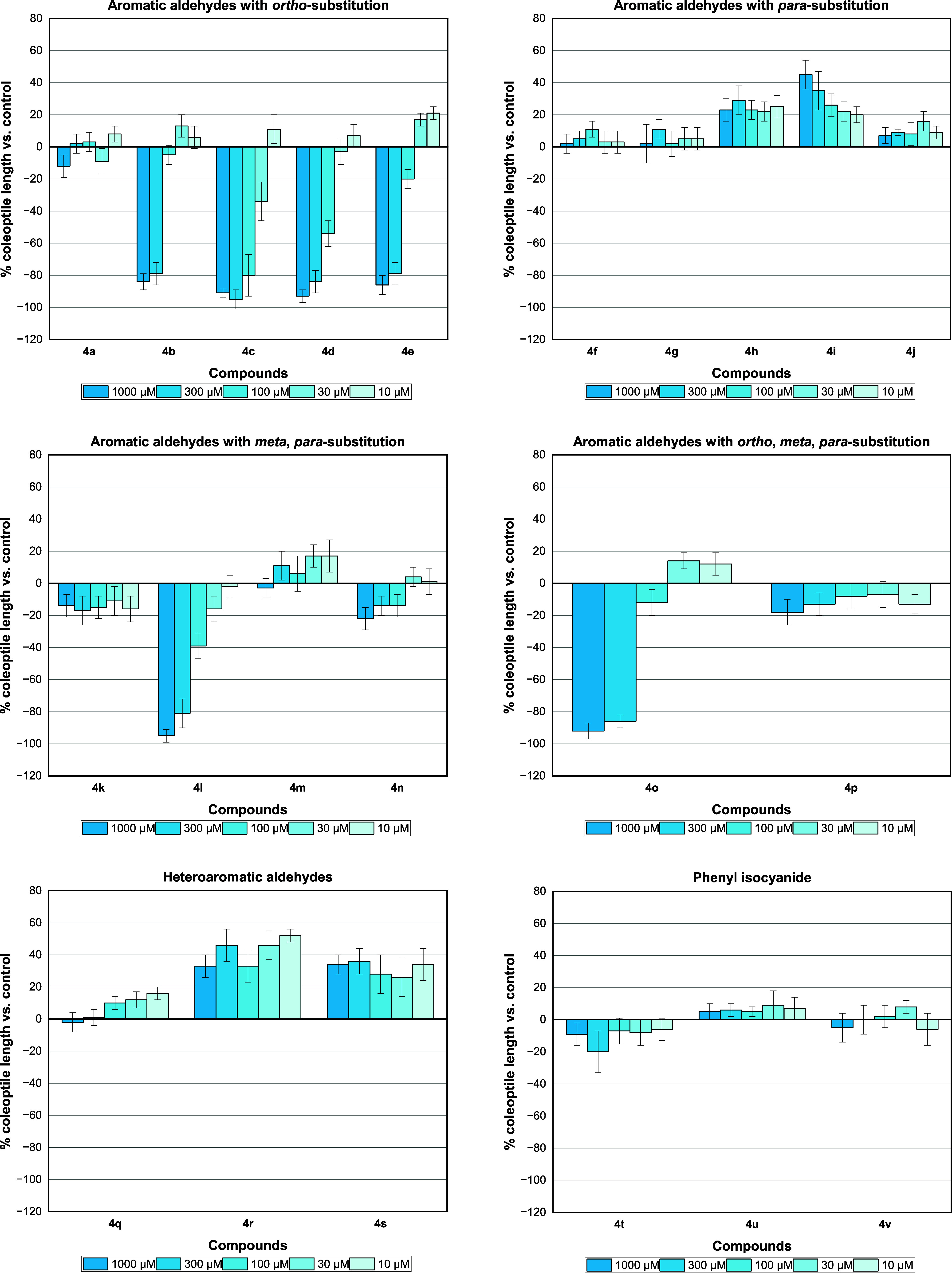
Effect of imidazo[1,2-*a*]pyridine derivatives **4a**-**4v** on wheat coleoptile elongation. Positive values indicate stimulation of growth vs the control, and negative values indicate inhibition. Each bar is the mean ± standard deviation.

**Figure 4:**
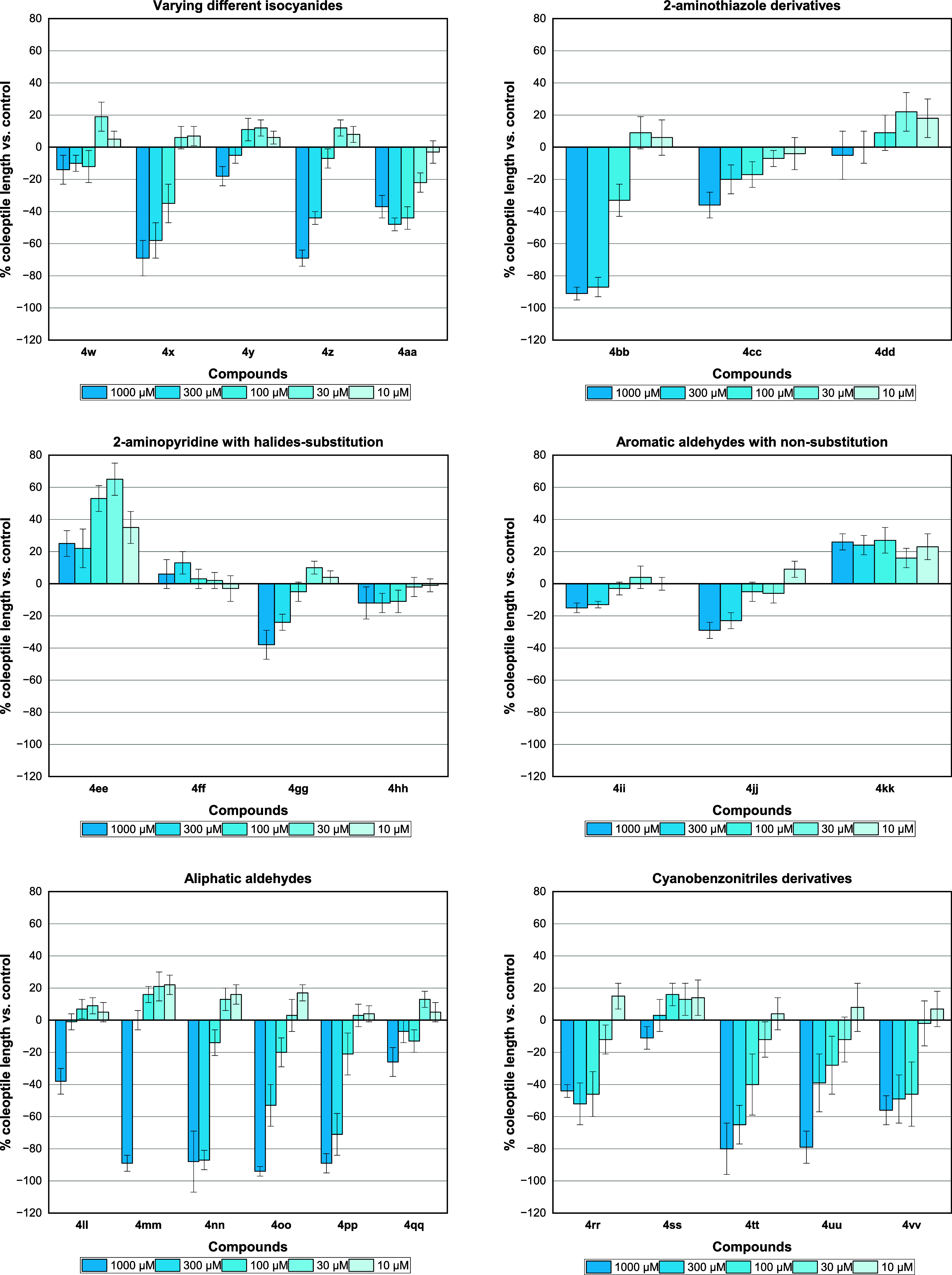
Effect of imidazo[1,2-*a*]pyridine derivatives **4w**-**vv** on wheat coleoptile elongation. Positive values indicate stimulation of growth vs the control, and negative values indicate inhibition. Each bar is the mean ± standard deviation.

**Figure 5:**
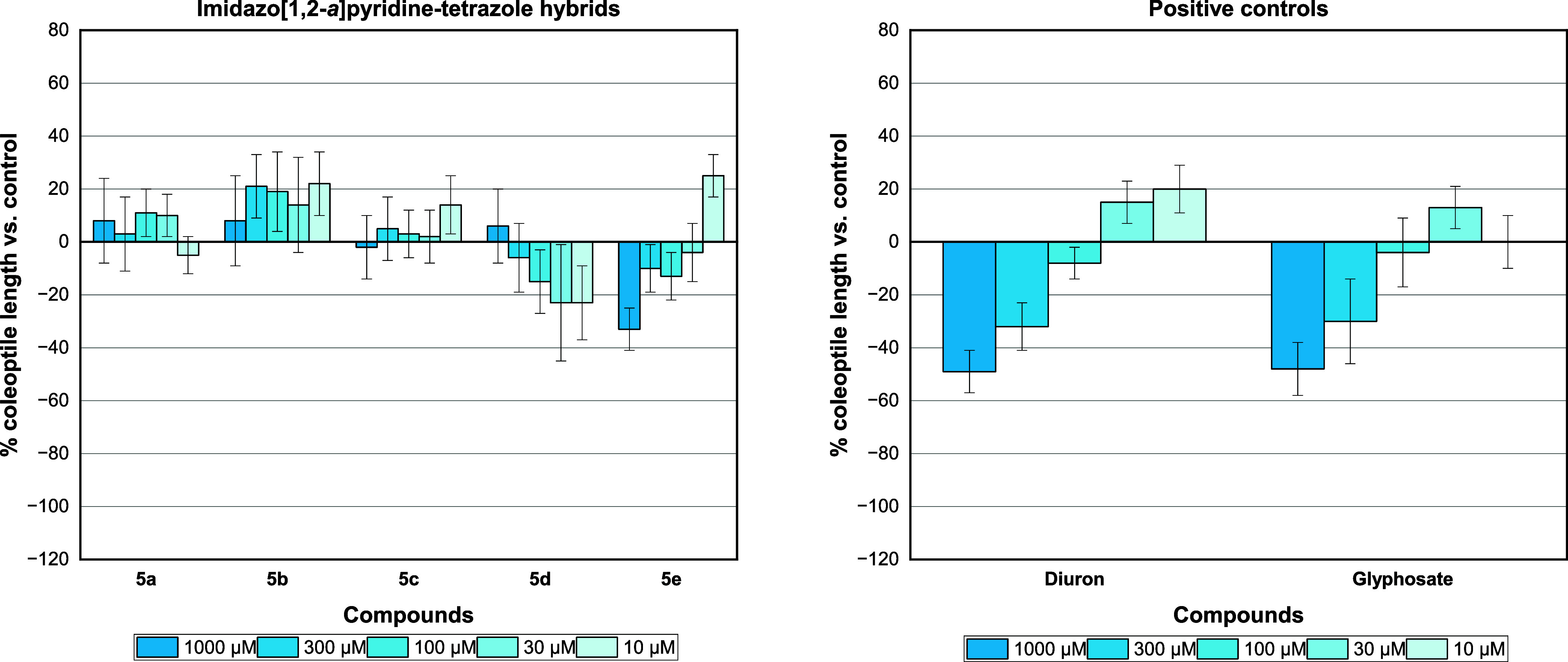
Effect of imidazo[1,2-*a*]pyridine-tetrazole hybrids **5a**-**e** and positive controls (diuron and glyphosate) on wheat coleoptile elongation. Positive values indicate stimulation of growth vs the control, and negative values indicate inhibition.

As already mentioned, there is no information available on the phytotoxic properties of simple GBB adducts. Thus, we first investigated the phytotoxicity profile of these compounds through the etiolated wheat coleoptile bioassay of compounds **4a**-**vv** (Figures 2, 3, 4 and 5). Bioassays using coleoptiles for phytotoxicity assessment have been used for both natural and synthetic compounds and are well documented in the literature.^29,37−40^

*Ortho*-substituted compounds bearing electron-donating groups such as hydroxyl (**4a**) were much less active or not active at all with low inhibition (12%) at the higher concentration (1000 μM) (Figure 2). Electron-withdrawing groups in this structure (**4b**-**e**) seem to play a key role in the activity once high inhibition rates were observed for the first two concentrations (1000 and 300 μM). The compound with bromine (**4c**) was the most active and resulted in an inhibition of 80% at the third tested concentration (100 μM). For comparison, chlorine **4d** (54%) or fluorine **4e** (20%) showed much lower effects at the same concentration (Figure 3).

For *para*-substituted compounds (**4f**-**j**), no activity was observed for those containing strong electron-withdrawing groups (CN **4f** or NO_2_**4g**). Interestingly, stimulatory growth activity was noted for compounds containing halide substituents (**4h**-**j**), especially chlorine **4i**, in which there was a dose–response profile according to the concentration tested. These results are intriguing compared with the inhibition activity observed for compound **4d** with chlorine at the *ortho* position.

Compound **4k** with a NO_2_ group at *meta*-substitution did not show appreciable phytotoxic activity. However, *meta*, *para*- and *ortho*,*para*-OMe compounds (**4l** and **4o**, respectively) showed a strong inhibition, and the activity was 81 and 86%, respectively, at the second tested concentration (300 μM). Nevertheless, the introduction of a hydroxyl group in the *para* position (**4m**) or a dioxolane ring (**4n**) resulted in a loss of activity.

The loss of phytotoxic activity for OH-substituted GBB adducts was verified with the use of 5-bromosalicylaldehyde (**4p**), in which the inhibition activity was again suppressed. This fact was also observed for the structure containing the indole derivative (**4q**). Furthermore, the presence of heteroaromatic rings (**4r** or **4s**) brought about a stimulatory effect instead.

Noteworthy, the exchange of the cyclohexyl ring for a planar phenyl ring (**4t**-**v**) resulted in neither inhibition nor stimulus activity for compounds containing bromine or chlorine at the ortho position. The use of other aliphatic groups (**4w**-**aa**) demonstrated that the cyclohexyl ring is essential for the phytotoxic activity of the *ortho*-Br derivative (**4c**). Indeed, the use of a bulky *tert*-butyl group (**4w**) or a long carbon chain (**4y**) did not show a significant effect on the growth. In addition, moderate inhibition (69%) was observed for the highest concentration (1000 μM) of octyl (**4x**) or methyl cyanoacetate (**4z**) groups (Figure 4).

A study was then carried out to verify the influence of different bicyclic portions on the activity, maintaining the *ortho*-Br-substituted phenyl ring. The study began with the use of 2-aminothiazole derivatives (**4bb**-**dd**) in which an 87% inhibition was achieved only for the 2-aminothiazole compound **4bb**, even at the second concentration (300 μM), whereas **4cc** and **4dd** did not show significant activity.

Next, the presence of halides in the 2-aminopyridine structure (**4ee**-**hh**) was studied. An interesting stimulus at low concentrations (30 and 10 μM) was observed for the compound containing bromine in position 3 (**4ee**) compared to the lead compound **4c**. The presence of chlorine atoms in positions 4 (**4gg**) or 5 (**4hh**) did not show substantial inhibition or stimulus activity. These results demonstrated that the absence of substituents on the pyridine moiety of the imidazo[1,2-*a*]pyridine ring is indispensable for the observed activity in the wheat coleoptile elongation assay.

Nonsubstituted aromatic compounds (**4ii**-**kk**) were also tested in this assay, and low inhibition activities of 15% and 29% at the first concentration (1000 μM) were observed for the adducts containing the 2-aminopyridine (**4ii**) or 2-aminobenzothiazole (**4jj**) moiety, respectively. Differently, the 2-aminopyridine derivative containing a chlorine atom in position 5 (**4kk**) showed stimulus activity.

Finally, the influence of aliphatic groups (compounds **4ll**-**qq**) on the activity was investigated. Overall, these compounds showed high inhibition growth (up to 94%) only at higher doses (1000 μM) in the wheat coleoptile elongation assay. In contrast, these compounds can stimulate growth at lower doses (30 and 10 μM).

To verify the possible interference of electron-withdrawing groups and their relationship with the 2-aminopyridine and isocyanide components, cyanobenzonitrile aldehyde was selected as the standard for synthesizing the GBB **4rr-vv** adducts. These adducts were then used in the cycloaddition reaction to obtain imidazo[1,2-*a*]pyridine-tetrazole hybrids **5a**–**e**. Thus, etiolated wheat coleoptile bioassay measurements were carried out and compared with negative and positive controls. (Figure 5)

For the imidazo[1,2-*a*]pyridine structures, promising results were obtained, indicating that changes in the isocyanide group influence the phytotoxic activity. The presence of halide in the imidazo ring and the ester group favored inhibition by nearly 80% at the highest concentration (1000 μM). Additionally, it is worth noting that the presence of cyclohexyl and phenyl groups resulted in no inhibition or growth stimulation, unlike aldehydes with substituents in the *ortho* position.

Although the GBB products demonstrated excellent phytotoxic activity, replacing the nitrile group with tetrazole resulted in stimulation compared with the negative control in almost all molecules. The exception was molecule **5e**, which showed a slightly satisfactory result. This confirms that the *meta*-position favored inhibition under both conditions, with the presence of the tetrazole group acting as a stimulating factor.

**Scheme 3:**
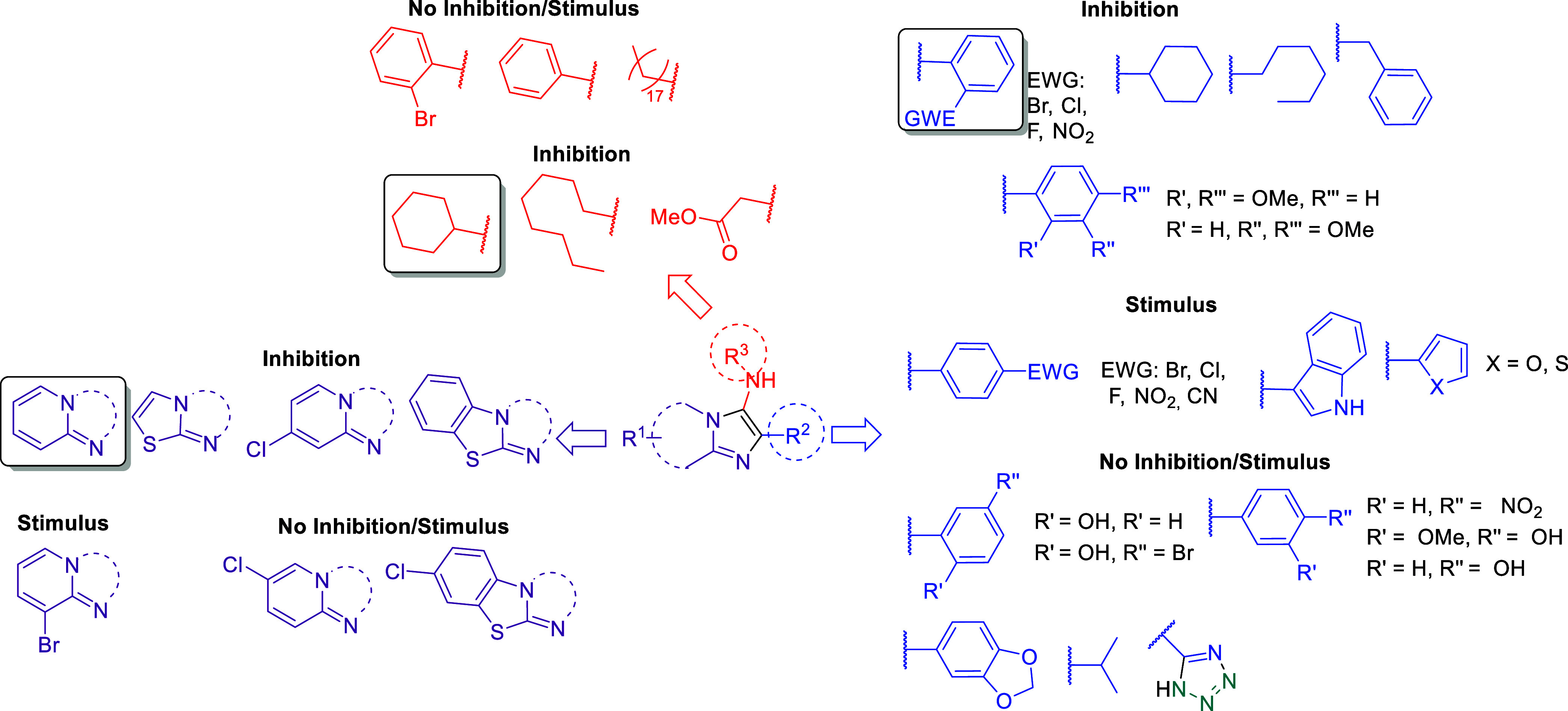
Rationale of the Structure/Activity Relationship for Phytotoxicity of GBB Adducts on Wheat Coleoptiles

In summary, the presence of an *ortho*-Br or -Cl at the imidazo[1,2-*a*]pyridine ring combined with a cyclohexyl group is essential for a good phytotoxic profile, giving a strong inhibition up to the third concentration (300 μM). These values were even higher than those achieved using the commercial herbicides diuron and glyphosate, which had a moderate inhibition activity of 49 and 48% in the first concentration (1000 μM), respectively. In view of these results, a structure–activity relationship could be established (Scheme 3).

The coleoptile bioassay results allowed us to calculate the IC_50_ value for each product evaluated through a sigmoidal dose–response curve model used to compare the IC_50_ values of the tested compounds (Table 1). Accordingly, it was confirmed that the most active compounds were **4c** (44.60 μM), **4d** (96.69 μM), and **4l** (122.2 μM). These values were much lower than those achieved by using the herbicides diuron (617.2 μM) and glyphosate (835.8 μM). These IC_50_ values corroborated our previous conclusions regarding the relative activities of the products on wheat coleoptile elongation.

**Table 1:** IC_50_ (μM) of Phytotoxicity Bioassays on Wheat Seed Coleoptiles for the Different Compounds Tested in This Work

entry	compound	IC_50_ (μM)	log IC_50_	*R*^2^
1	**4a**	7402	3.869	0.9794
2	**4b**	214.6	2.332	0.9097
3	**4c**	44.60	1.649	0.9395
4	**4d**	96.69	1.985	0.9653
5	**4e**	168.9	2.228	0.9287
6	**4f**	-a	-	-
7	**4g**	-a	-	-
8	**4h**	-a	-	-
9	**4i**	-a	-	-
10	**4j**	-a	-	-
11	**4k**	-a	-	-
12	**4l**	122.2	2.087	0.9874
13	**4m**	7607	3.881	0.9776
14	**4n**	3203	3.506	0.9788
15	**4o**	176.9	2.248	0.9118
16	**4p**	6782	3.831	0.9876
17	**4q**	7964	3.901	0.9832
18	**4r**	-a	-	-
19	**4s**	-a	-	-
20	**4t**	-a	-	-
21	**4u**	-a	-	-
22	**4v**	-a	-	-
23	**4w**	3961	3.598	0.9449
24	**4x**	199.1	2.299	0.9617
25	**4y**	3186	3.503	0.9846
26	**4z**	395.4	2.597	0.9764
27	**4aa**	1097	3.040	0.7909
28	**4bb**	137.1	2.137	0.9408
29	**4cc**	1852	3.268	0.9880
30	**4dd**	4960	3.695	0.9683
31	**4ee**	-a	-a	0.8551
32	**4ff**	-a	-a	0.9836
33	**4gg**	1224	3.088	0.9808
34	**4hh**	8058	3.906	0.9880
35	**4ii**	4710	3.673	0.9904
36	**4jj**	1923	3.284	0.9720
37	**4kk**	-a	-	-
38	**4ll**	1671	3.223	0.9802
39	**4 mm**	592.5	2.773	0.8756
40	**4nn**	168.2	2.226	0.9124
41	**4oo**	234.4	2.370	0.9695
42	**4pp**	194.3	2.288	0.9692
43	**4qq**	2415	3.383	0.9673
44	**4rr**	137.1	2.137	0.8255
45	**4ss**	4216	3.625	0.9806
46	**4tt**	146.2	2.165	0.9907
47	**4uu**	329.5	2.518	0.9766
48	**4vv**	195.0	2.290	0.9002
49	**5a**	-a	-	-
50	**5b**	-a	-	-
51	**5c**	-a	-	-
52	**5d**	-a	-	-
53	**5e**	1624	3.210	0.9309
54	**Diuron**	617.2	2.790	0.9586
55	**Glyphosate**	835.8	2.922	0.9732

a 50% inhibition was not achieved at the highest concentration.

#### 3.2.2 Seed Bioassay

**Figure 6:**
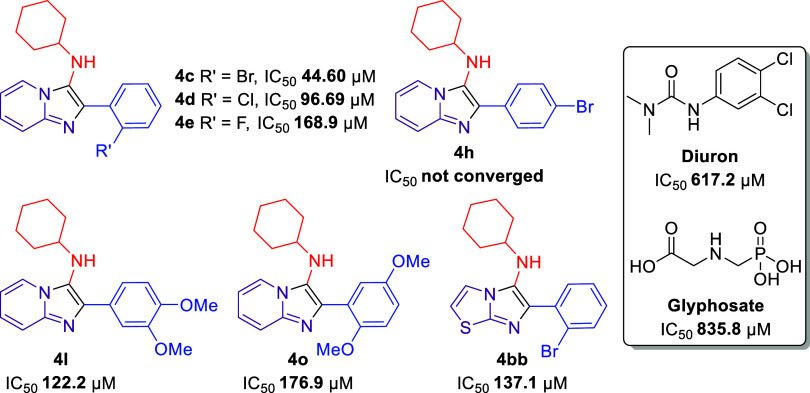
GBB adducts and herbicides (diuron and glyphosate) selected for phytotoxicity bioassay on seeds of *A. cepa*, *L. sativa*, and *S. lycopersicum*.

According to the phytotoxicity study for GBB adducts on wheat seed coleoptiles, the most active compounds were selected for the phytotoxic bioassay toward the most sensitive seeds of *A. cepa*, *L. sativa*, and *S. lycopersicum* (Figure 6). These are standard target species (STS) commonly used in seed bioassays.^39^ The selected compounds were **4c**, **4d**, **4e**, **4l, 4o**, and **4bb** for their higher activity in wheat coleoptile bioassays according to the IC_50_ values compared to the positive controls of diuron and glyphosate herbicides (Table 1). In addition, compound **4h** was also evaluated due to its observed stimulus activity.

In general, the inhibitory effect of the GBB adducts and diuron herbicide on seed germination was the parameter least affected and was more pronounced for root growth with high sensitivity than for shoot growth of the target species. As in the results described above, all compounds exhibited high inhibitory activity, especially at the highest tested concentration (1000 μM). This inhibition of shoot growth decreased quickly with dilution.

**Figure 7:**
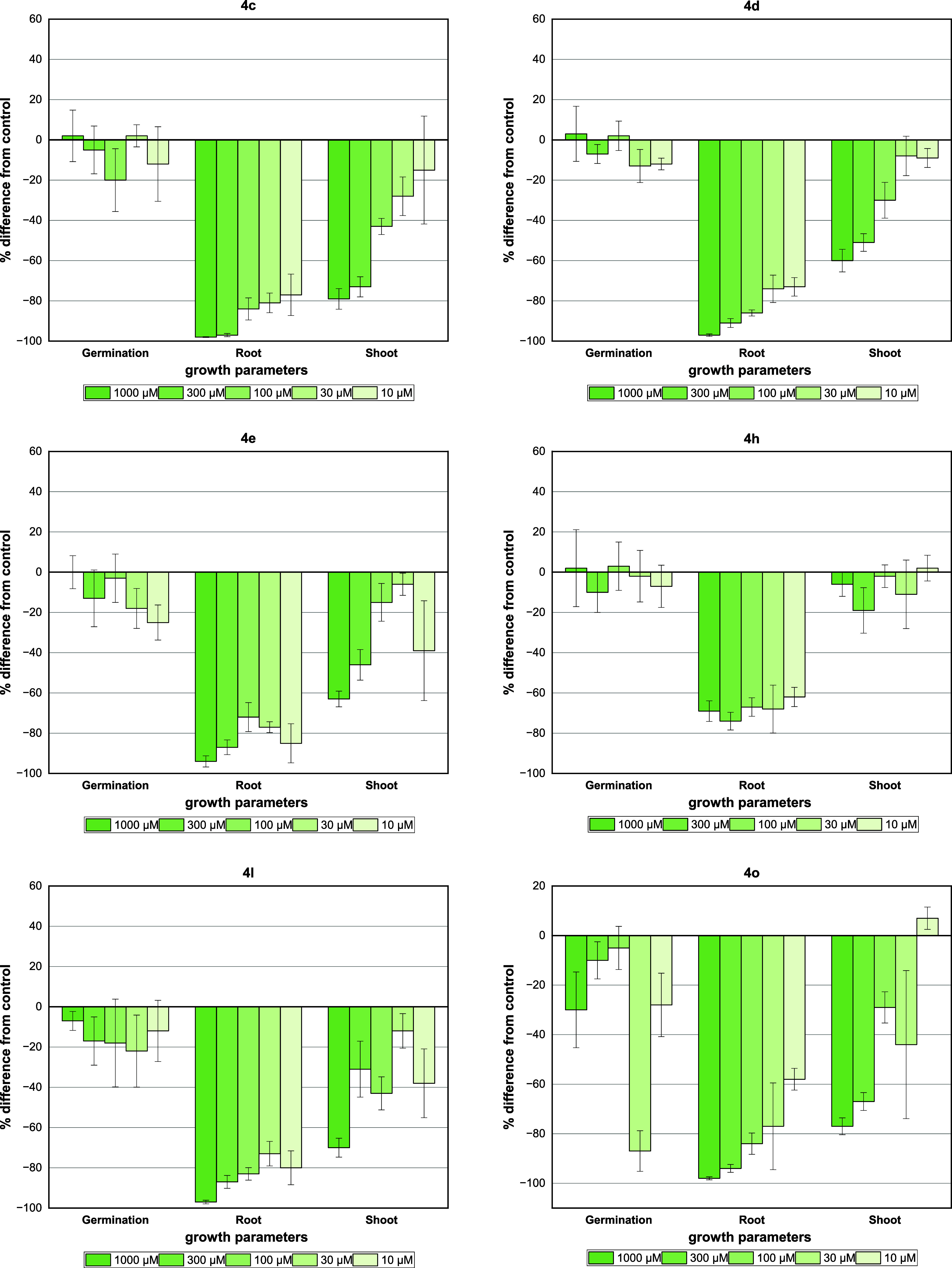

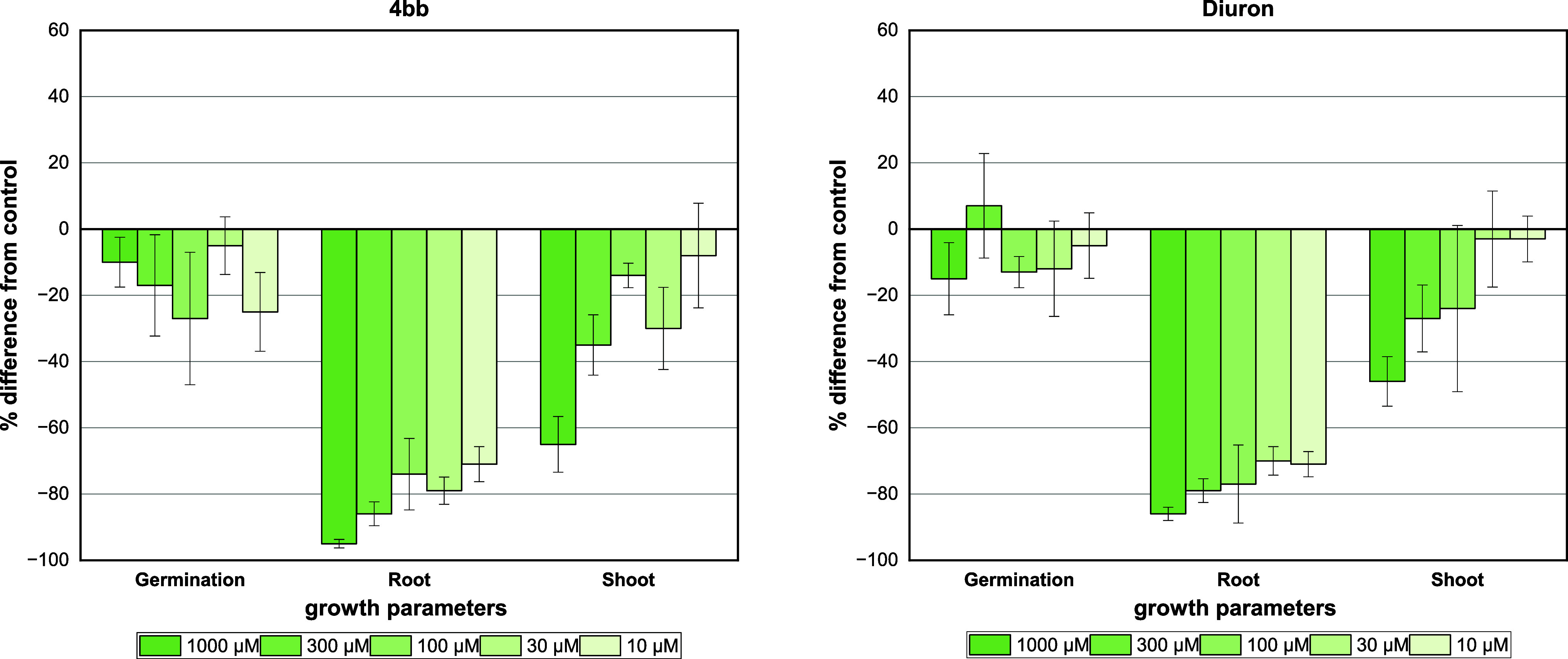
Effect of GBB adducts and DCMU herbicide (diuron) on germination and growth in *A. cepa* seeds. Positive values indicate stimulation of growth vs the control, and negative values indicate inhibition. Each bar is the mean ± standard deviation.

The results for germination, root, and shoot length of *A. cepa* seeds are shown in Figure 7. Most of the tested compounds did not show activity with regard to the germination rate of the weeds used in this bioassay. The inhibitory activity on the root length for the products evaluated was higher than that on the shoot length. In this case, the highest activities were found for compounds **4c** (98 and 79% inhibition), **4d** (97 and 60% inhibition), and **4e** (94 and 63% inhibition) for roots and shoot length at the highest tested concentration (1000 μM), respectively. These inhibitions are even better than those observed for diuron (86 and 46% inhibition).

Compound **4h** did not show stimulus activity as previously reported in the coleoptile bioassay, being very active at the root length (69% inhibition) but showed no activity on shoot length (6% inhibition). For the OMe-substituted compounds (**4l**, **4o**), an inhibition of over 77% at root growth was observed up to the fourth concentration (30 μM). However, high inhibition of the shoot length was obtained only in the first concentration (1000 μM). Similar results were observed for the derivative containing the 2-aminothiazole group (**4bb**).

According to the IC_50_ values from the results on germination, root, and shoot length (Table 2), the most active compounds were found to be **4c** (IC_50_ = 0.3012 and 11.56 μM), **4e** (IC_50_ = 0.09213 and 66.76 μM), and **4o** (IC_50_ = 0.7309 and 14.32 μM) for roots and shoot length, respectively.

**Table 2:** IC_50_ Values (μM) Obtained from the Inhibition on All Growth Parameters Analyzed for *A. cepa* Seeds

		germination rate		root		shoot	
entry	compound	IC_50_ (μM)	*R*^2^	IC_50_ (μM)	*R*^2^	IC_50_ (μM)	*R*^2^
1	**4c**	-a	-	0.3012	0.9788	11.56	0.9821
2	**4d**	-a	-	0.3877	0.9742	35.77	0.9683
3	**4e**	-a	-	0.0921	0.9268	66.76	0.8645
4	**4h**	-a	-	0.3345	0.8812	1,768	0.9630
5	**4l**	-a	-	0.2305	0.9519	68.89	0.8372
6	**4o**	0.1501	0.1691	0.7309	0.9923	14.32	0.8847
7	**4bb**	-a	-	0.3304	0.9519	67.88	0.9428
8	**Diuron**	1288	0.9577	0.2969	0.9408	105.0	0.9743

a 50% inhibition was not achieved at the highest concentration.

**Figure 8:**
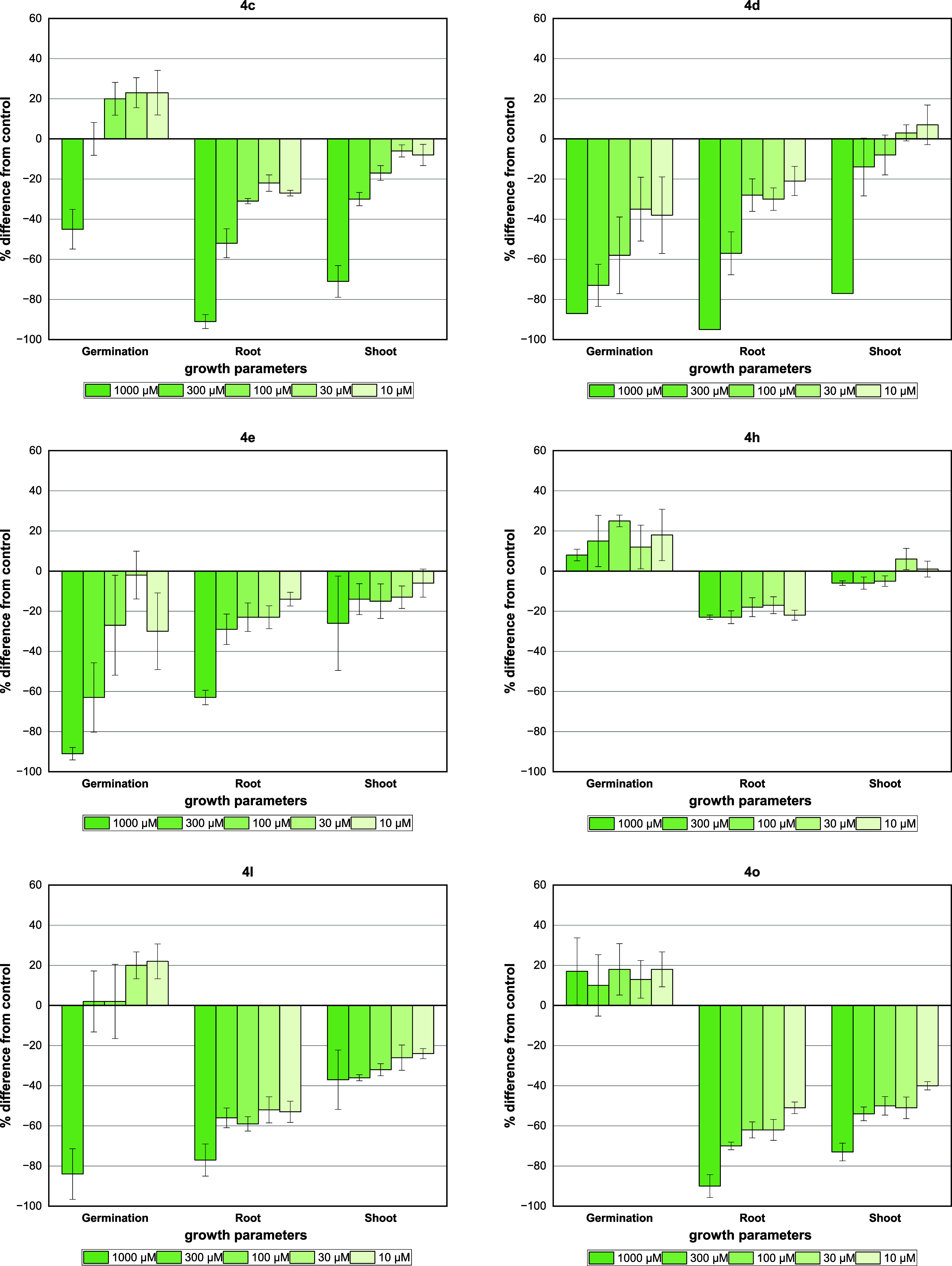

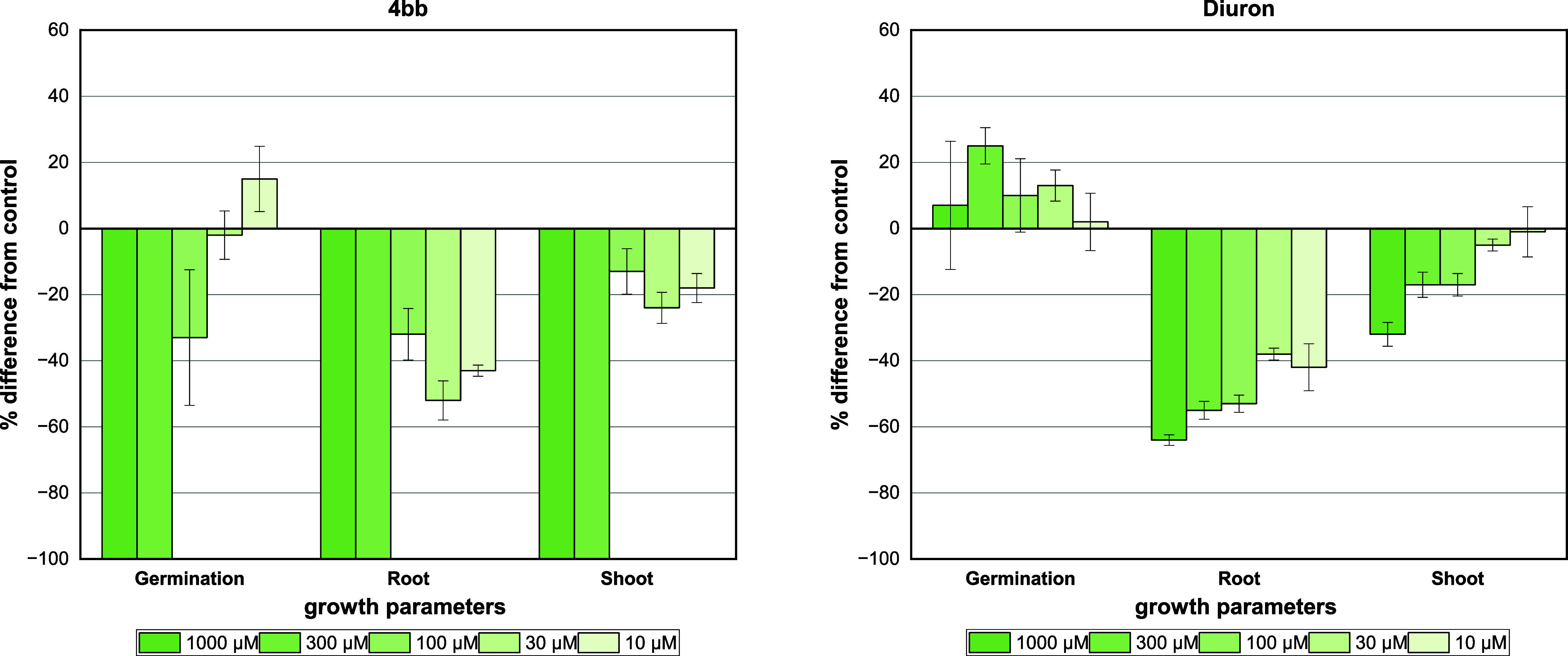
Effect of GBB adducts and DCMU herbicide (diuron) on germination and growth of *L. sativa* seeds. Positive values indicate stimulation of growth *v*s the control, and negative values indicate inhibition. Each bar is the mean ± standard deviation.

All tested compounds showed a significant effect on the germination rate of the *L. sativa* seed especially compounds **4d**, **4e**, and **4l** in which around 91–87% inhibition was found at the highest tested concentration, whereas a stimulus effect was observed for compounds **4h**, **4o**, and diuron (Figure 8). Interestingly, the same effect was obtained for adduct **4c** under higher dilution conditions. Compounds **4c** (91% inhibition), **4d** (95% inhibition), and **4o** (90% inhibition) were the most active against the root length with superior inhibition activities compared to diuron (64% inhibition) at a concentration of 1000 μM.

For shoot length, *L. sativa* was the most sensitive to the inhibitory effects of compounds **4c**, **4d**, and **4o** with inhibition values between 77 and 71% at the first concentration (1000 μM). In contrast, compound **4h** did not show a significant effect on the root and shoot length. Interestingly, adduct **4bb** showed a complete inhibition in the first two concentrations (1000 and 300 μM) on growth parameters of the *L. sativa*.

Table 3 summarizes the IC_50_ values (μM) obtained from the inhibition of all of the growth parameters of *L. sativa*. The introduction of chlorine or bromine atoms at the *ortho* position (**4e**) or 2-aminothiazol (**4bb**) at the GBB adducts improves the activity against *L. sativa* seeds with lower values of IC_50_ (Table 3). Furthermore, compounds **4l** and **4o** also demonstrated excellent IC_50_ values for root growth inhibition.

**Table 3:** IC_50_ Values (μM) Obtained from the Inhibition on All Growth Parameters Analyzed for *L. sativa* Seeds

		germination rate		root		shoot	
entry	compound	IC_50_ (μM)	*R*^2^	IC_50_ (μM)	*R*^2^	IC_50_ (μM)	*R*^2^
1	**4c**	111.9	0.9486	29.36	0.9486	57.18	0.9888
2	**4d**	6.865	0.9341	25.36	0.9472	60.83	0.9516
3	**4e**	25.17	0.9216	79.26	0.9641	374.2	0.9844
4	**4h**	2,704	0.9686	695.0	0.9515	1,187	0.9913
5	**4l**	61.17	0.8885	0.7609	0.7823	254.8	0.8924
6	**4o**	-a		0.9633	0.8862	27.17	0.7657
7	**4bb**	11.93	0.9405	9.774	0.7910	15.48	0.8520
8	**Diuron**	-a		29.07	0.7696	217.3	0.9849

a 50% inhibition was not achieved at the highest concentration.

**Figure 9:**
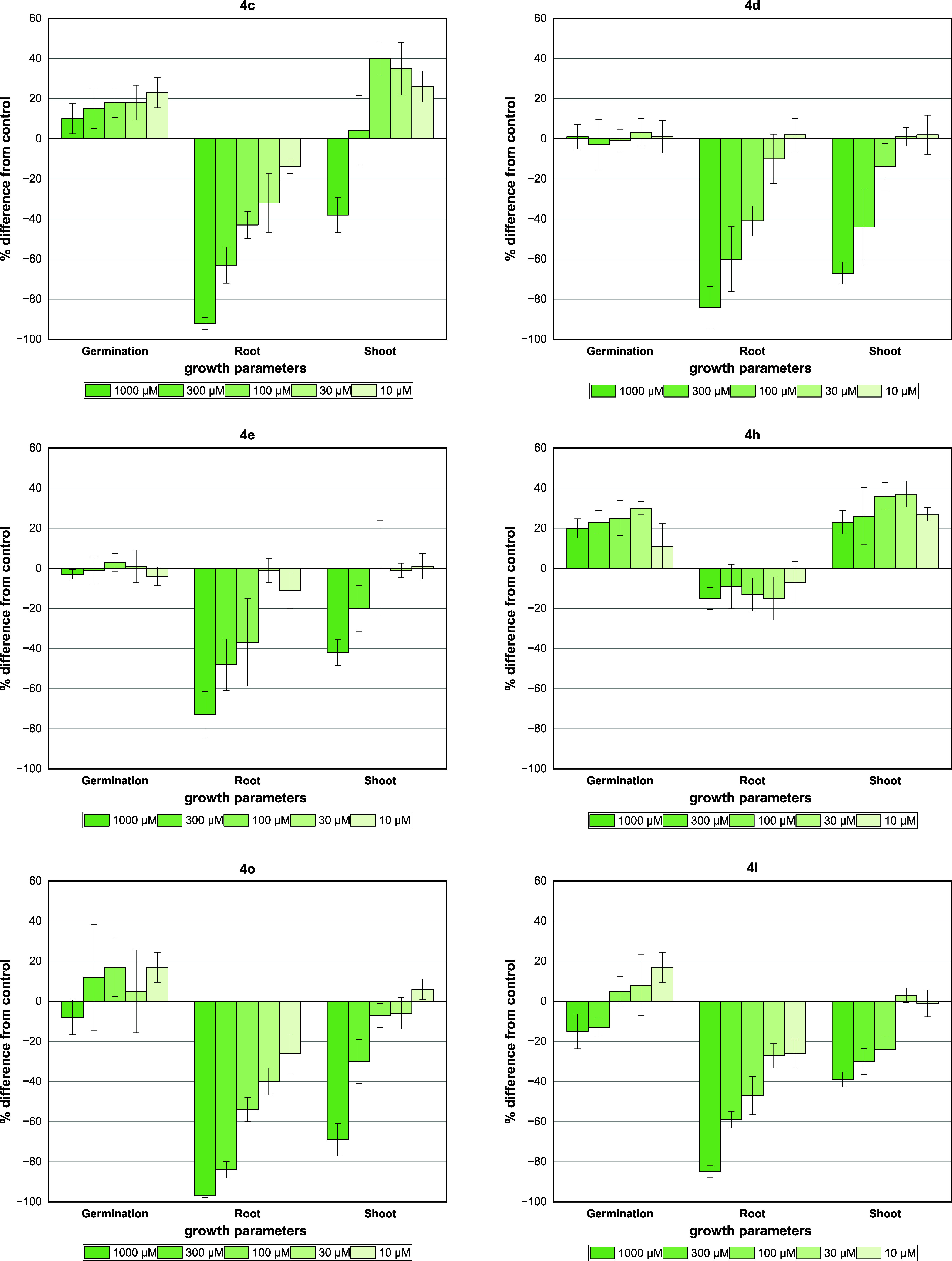

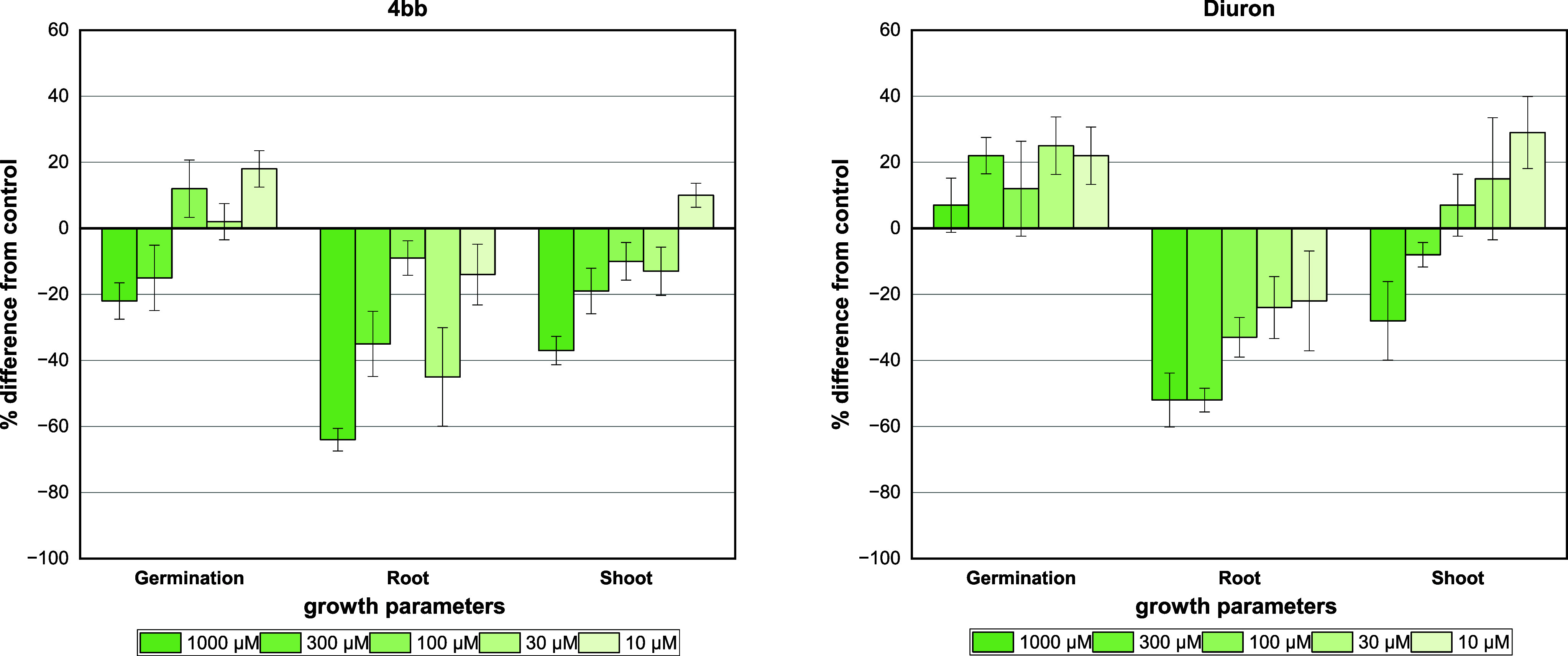
Effect of GBB adducts and DCMU herbicide (diuron) on germination and growth of *S. lycopersicum* seeds. Positive values indicate stimulation of growth *v*s the control, and negative values indicate inhibition. Each bar is the mean ± standard deviation.

The GBB adducts **4c** and **4h** and diuron caused a significant stimulation in the *S. lycopersicum* germination rate growth in at least one of the evaluated concentrations (Figure 9). However, compounds **4d**, **4e 4o**, **4l**, and **4bb** did not show a significant effect. Regarding the activity on the elongation of *S. lycopersicum* roots and shoots, the tested compounds **4c**, **4d**, **4e, 4l**, and **4o** showed inhibitory activity of 92, 84, 73, 85, and 97% on the root length at the first concentration of 1000 μM, respectively. Interestingly, for these compounds, a good dose–response relationship was observed. In contrast, compound **4h** did not show any significant activity.

Moderate activity was observed for **4bb** (64% inhibition) and diuron (52% inhibition) at the first concentration. Compounds **4d** and **4o** were the most active on *S. lycopersicum* at shoot length, with inhibition values of 67 and 69% at the highest concentration tested, whereas low values of inhibition were observed for products **4l** (39% inhibition), **4bb** (37% inhibition), and diuron (28% inhibition). A stimulation effect was observed for product **4h** similar to the results on the coleoptile bioassay.

The results obtained for *S. lycopersicum* indicate that this species is sensitive to compounds bearing halides or methoxy substituents at the *ortho-* or *meta-para-* positions of the aromatic ring with remarkable results for compounds **4c, 4d, 4l**, and **4o**. These compounds showed the highest inhibition of all growth parameters analyzed, which was corroborated through IC_50_ calculations (Table 4).

**Table 4:** IC_50_ Values (μM) Obtained from the Inhibition on All Growth Parameters Analyzed for *S. lycopersicum* Seeds

		germination rate		root		shoot	
entry	Compound	IC_50_ (μM)	*R*^2^	IC_50_ (μM)	*R*^2^	IC_50_ (μM)	*R*^2^
1	**4c**	2760	0.9726	14.45	0.9774	125.0	0.9038
2	**4d**	-a	-	16.19	0.9923	41.27	0.9935
3	**4e**	3609	0.9967	28.82	0.9691	129.8	0.9951
4	**4h**	-a	-	1,183	0.9817	-a	-
5	**4l**	308.8	0.9691	16.88	0.9585	118.3	0,9498
6	**4o**	594.9	0.9711	6.687	0.9771	55.77	0.9881
7	**4bb**	224.2	0.9599	83.99	0.8392	158.2	0.9677
8	**Diuron**	1606	0.9610	61.46	0.8978	156.9	0.9569

a 50% inhibition was not achieved at the highest concentration.

**Figure 10:**
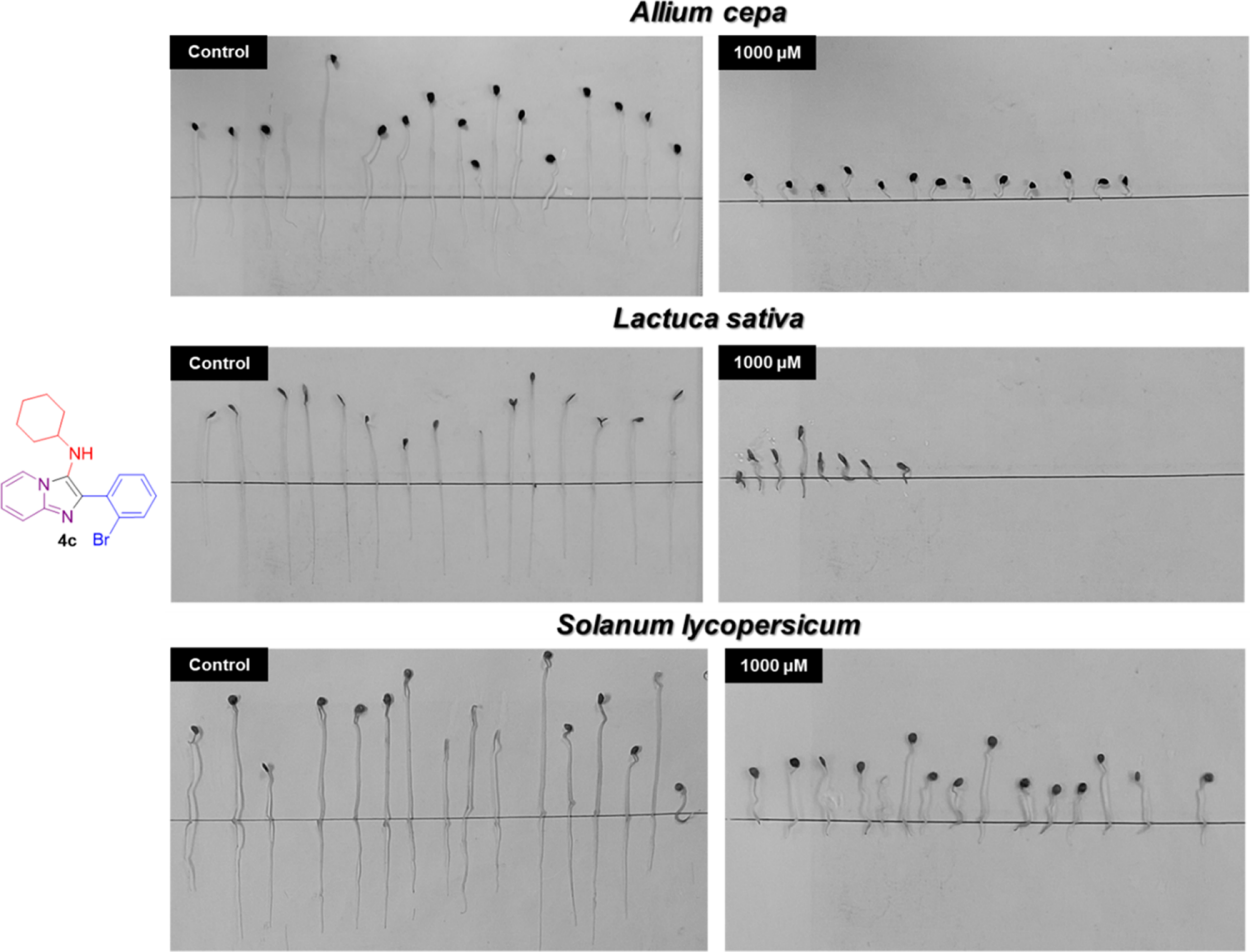
Effect of GBB adduct **4c** on germination and growth in sensitive seeds of *A. cepa*, *L. sativa*, and *S. lycopersicum*.

Finally, the preliminary results of wheat coleoptile elongation of the GBB adducts showed that adducts **4c, 4d, 4l**, and **4o** were the most phytotoxic, highlighting compound **4c** containing the bromine atom at the *ortho* position of the aromatic ring, which showed better results in the etiolated wheat coleoptile bioassay, a fact that was corroborated through the phytotoxic bioassay to the most sensitive seeds of *A. cepa*, *L. sativa*, and *S. lycopersicum* (Figure 10).

#### 3.2.3 Hydroponic Bioassay

A further study to confirm the phytotoxicity activity of these compounds was carried out under hydroponic conditions using three different weeds: *B. pilosa*, *U. decumbens*, and *P. maximum*. The choice for these species resides in the fact that they are common invasive species in Brazil, which are usually combatted with traditional herbicides.^41−43^ The GBB adduct **4c** was selected for this hydroponic bioassay due to its significant inhibitory effects on germination and growth in sensitive seeds such as *A. cepa*, *L. sativa*, and *S. lycopersicum*. Additionally, diuron was chosen as a positive control in this bioassay.

**Figure 11:**
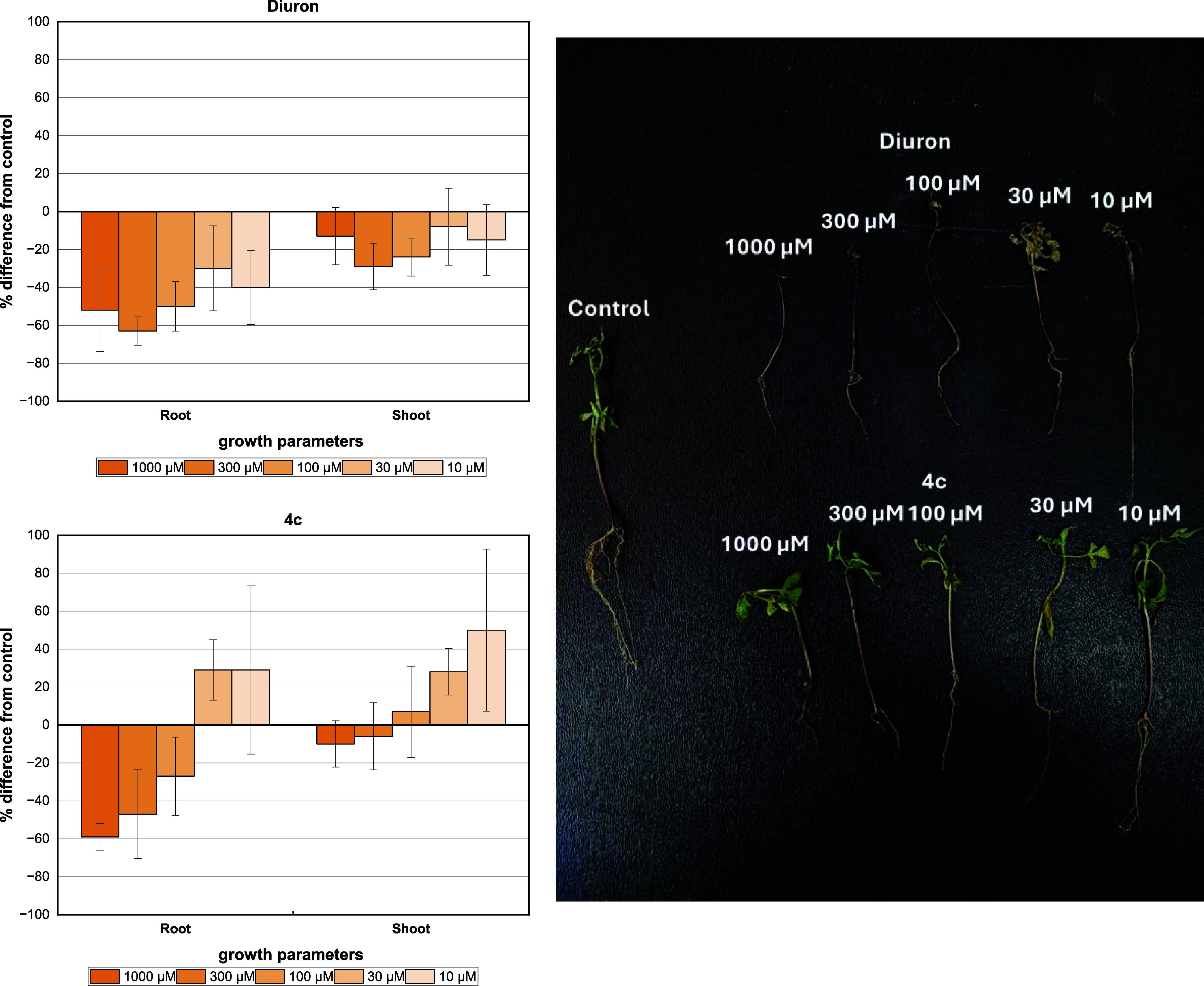
Effect of the DCMU herbicide (diuron) and GBB adduct **4c** on the hydroponic growth of shoot and root on *B. pilosa*. Positive values indicate stimulation of growth vs the control, and negative values indicate inhibition. Each bar is the mean ± standard deviation.

The GBB adduct **4c** exhibited a good dose–response relationship against the root length of *B. pilosa* (Figure 11). This growth parameter demonstrated sensitivity to the compound with the highest concentration (1000 μM) resulting in a reduction of 59%, surpassing the efficacy of diuron (52%). However, the effect on the aerial part of the plant was less satisfactory. In contrast, for diuron, the inhibition rates remained constant within the concentration ranges tested, accompanied by visible necrosis of the leaves.

**Figure 12:**
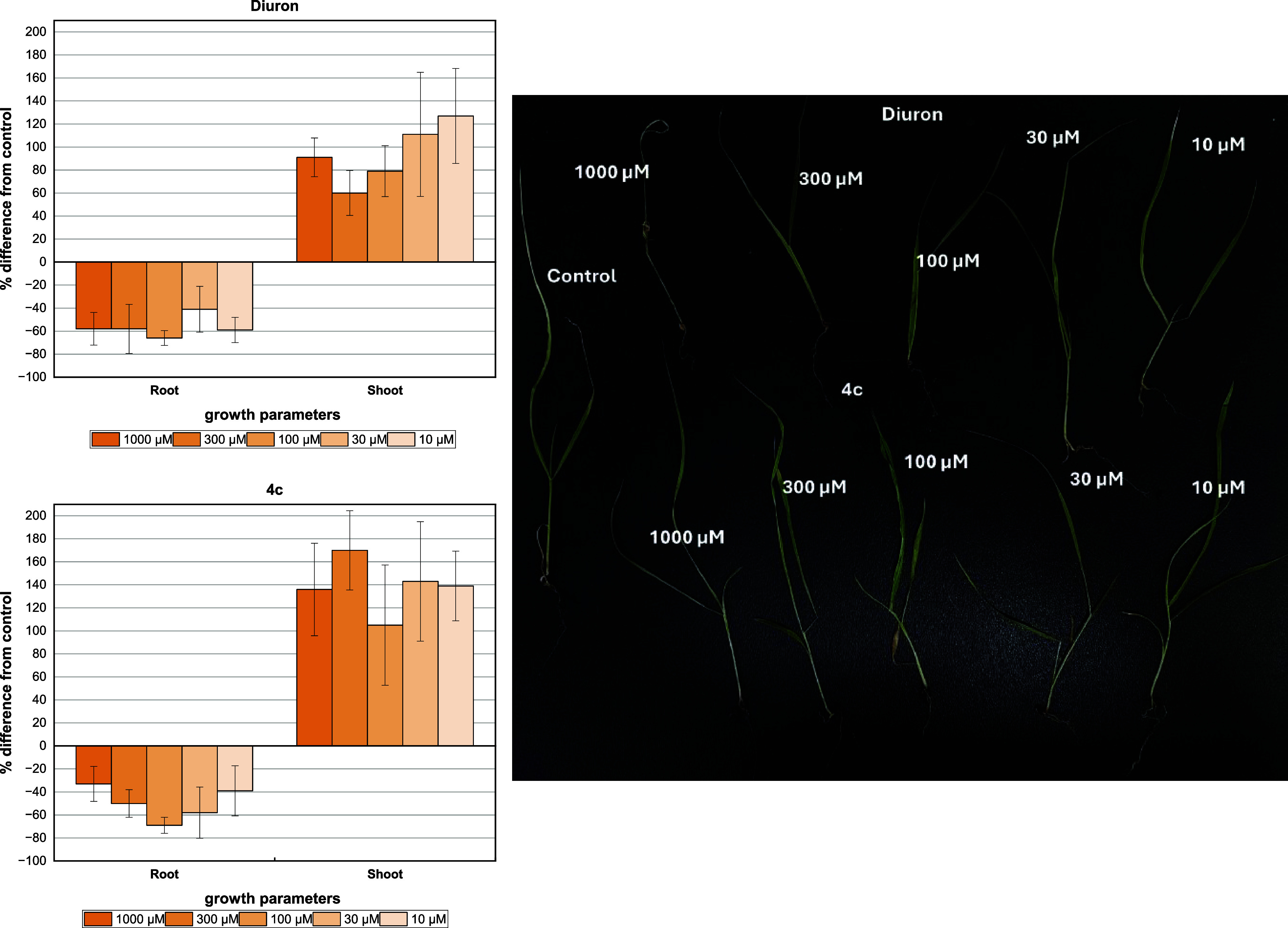
Effect of the DCMU herbicide (diuron) and GBB adduct **4c** on the hydroponic growth of shoot and root on *U. decumbens*. Positive values indicate stimulation of growth vs the control, and negative values indicate inhibition. Each bar is the mean ± standard deviation.

Compound **4c** showed a good and effective inhibition profile over the root length of *U. decumbens* in all tested concentrations (1000–10 μM) (Figure 12). The inhibitory effects ranged from 39 to 69%, similar to those of diuron (41–66%). In contrast, no inhibition was observed on the growth of shoots by any of the compounds tested. Interestingly, a large stimulation effect was observed, even at the fifth concentration (10 μM). **4c** maintained shoot growth stimulation levels of over 139% for this species, a result close to that found for diuron (127%). Nonetheless, necrosis in the aerial part was not noted.

**Figure 13:**
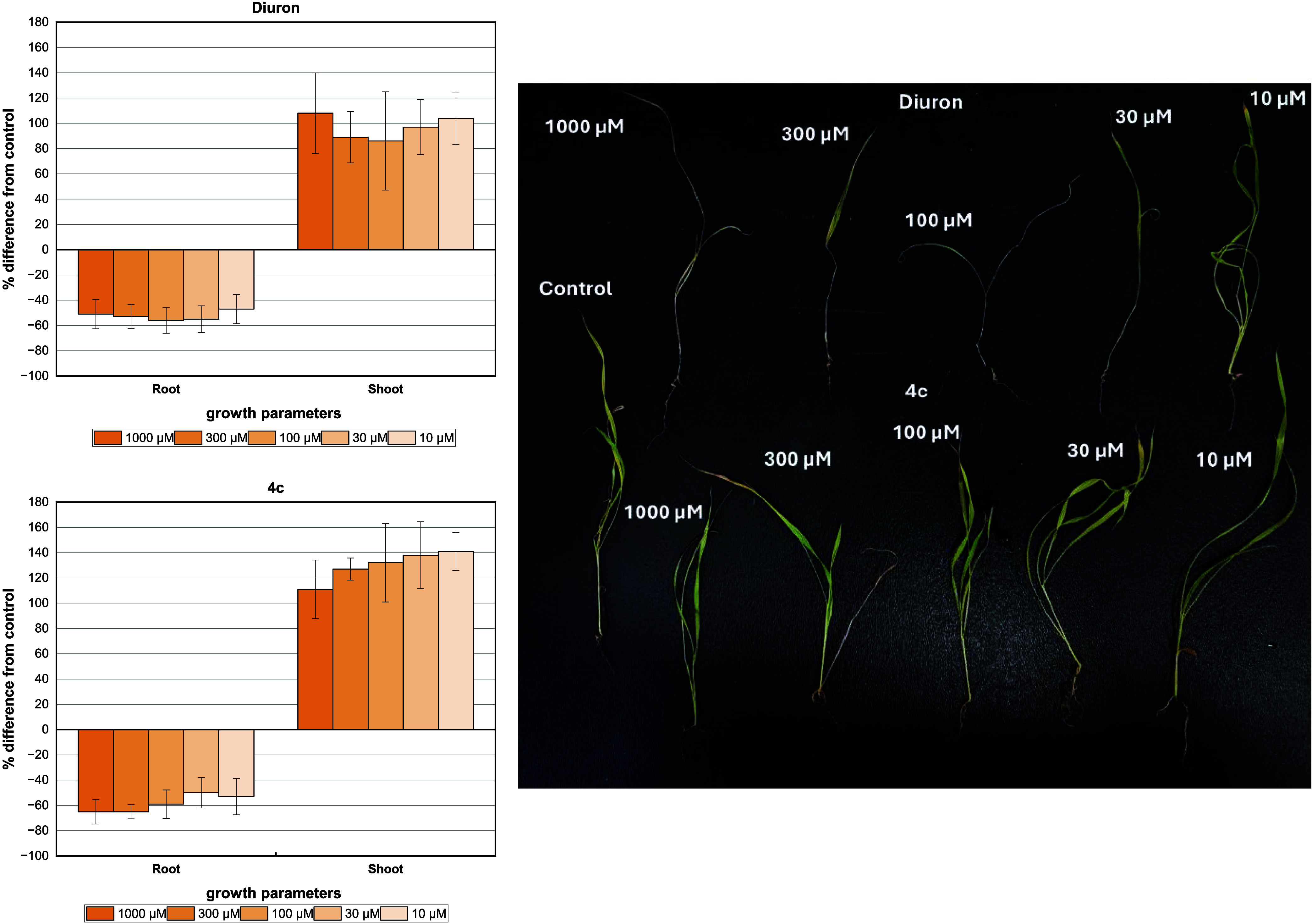
Effect of the DCMU herbicide (diuron) and GBB adduct **4c** on the hydroponic growth of shoot and root on *P. maximum*. Positive values indicate stimulation of growth vs the control, and negative values indicate inhibition. Each bar is the mean ± standard deviation.

Similar results were found on the shoot lengths of *P. maximum* when exposed to compound **4c**, exhibiting similar activities to that of diuron (Figure 13), whereas a high stimulus effect was observed compared with the negative control. In terms of the growth parameters, the roots were significantly inhibited in all tested concentrations (1000–10 μM) with inhibition levels ranging from 50 to 65% and from 41 to 56% for compound **4c** and diuron, respectively. Therefore, inhibition was observed even at the lowest concentration (10 μM).

The results obtained herein indicate that the GBB adduct **4c** is phytotoxic to both eudicots (*B. pilosa*) and monocots (*U. decumbens* and *P. maximum*) weed species under hydroponic conditions. The lower inhibition rate observed in grasses may be due to their high resistance to phytotoxic compounds. As a result, **4c** can be considered a hit compound, which could potentially lead to the development of new herbicides for controlling these weed species. This work may represent a preliminary approach to the use of GBB adducts for weed control in agriculture.

In summary, imidazo[1,2-*a*]pyridine derivatives were prepared by the Groebke–Blackburn–Bienaymé three-component reaction using easily accessible and cheap reagents under HPW catalysis, and their phytotoxicity properties were studied. Initially, the derivatives were evaluated through an etiolated wheat coleoptile bioassay. The results from this bioassay revealed an interesting structure–activity relationship in which the aldehydes *ortho*-substituted with electron-withdrawing groups such as bromine or chlorine combined with a cyclohexyl group and the imidazopyridine ring without any substituent were essential for phytotoxic activity providing a strong inhibition until the third dose. Differently, these groups in the *para* position showed a stimulus effect.

Next, the phytotoxic bioassay was selected for more sensitive seeds (*A. cepa*, *L. sativa*, and *S. lycopersicum*) in which the phytotoxic potential for the GBB adduct **4c** was confirmed, as it demonstrated the highest inhibition on all growth parameters analyzed of germination rate and root and shoot length compared to the positive control, the commercial herbicide diuron. The results obtained make this molecule a promising hit in the search for new herbicides, which is currently under investigation.

Furthermore, a mild and efficient method was developed for the synthesis of monosubstituted tetrazoles under microwave irradiation in the presence of ZnCl_2_. This method was subsequently used to promote a [3 + 2] cycloaddition reaction in the GBB products (**4rr**-**vv**) to obtain the corresponding tetrazolic compounds (**5a**-**e**). The imidazo[1,2-*a*]pyridine structures demonstrated promising results; however, the substitution of the nitrile group for a tetrazole did not increase the activity of the compounds, except for **5e**. Interesting results were also achieved against *B. pilosa*, *U. decumbens*, and *P. maximum* weeds, in which the growth factor most affected by inhibition was root growth, and a stimulus to shoot growth was noted. Due to the nature of the bioassays and the results obtained herein, we believe the application of the active compounds with herbicidal potential can be evaluated as postemergent because the most important results of inhibition are expressed in the initial development of the plants (seed bioassay) and also in the development phase (hydroponics bioassay).

**Figure 14:**
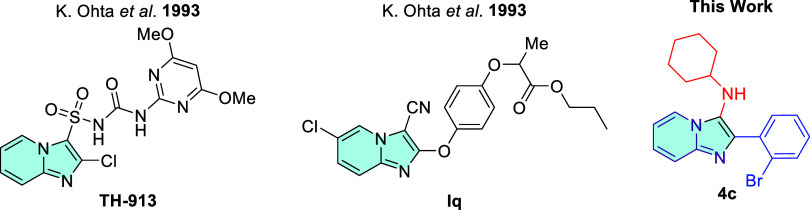
Structural comparison between sulfonylurea (**TH-193**) or phenoxypropionic acid (**Iq**) derivatives of imidazo[1,2-*a*]pyridines with GBB adduct **4c**.

The GBB-3CR methodology used in this study offers a faster, simpler, and more efficient approach to synthesizing a wide range of imidazo[1,2-*a*]pyridine derivatives. When compared to the results by Ohta et al.,^26,27^ ours showed different trends, primarily due to differences in the substituents attached to the imidazo[1,2-*a*]pyridine core and to the methodology. The most active sulfonylurea (**TH-193**) or phenoxypropionic acid (**Iq**) derivatives featured a lack of substituents or the presence of a chlorine atom at the 5-position of the pyridine ring, along with a chlorine or a nitrile group attached to the imidazole ring (Figure 14). In our findings, the absence of substituents was not shown to be essential. However, the presence of an *N*-cyclohexyl group, derived from the isocyanide component, was determined to be crucial for the observed phytotoxicity profile activity. As the mechanism of action of these molecules as well as their ecotoxicity are still unknown, additional studies still must be carried out.

## Supporting Information Available

Typical experimental procedures and spectroscopic data (FT-IR, NMR, and mass spectra) of new compounds studied in this article (PDF)
