# Supplementary material for: Phytotoxicity Study of (Amino)imidazo[1,2-a]pyridine Derivatives Toward the Control of Bidens pilosa, Urochloa decumbens, and Panicum maximum Weeds
Source: J Agric Food Chem. 2024 Dec 28;73(1):298–317. doi: 10.1021/acs.jafc.4c09477 (PMC11726675; doi:10.1021/acs.jafc.4c09477)
Supplement: Supplementary file 1 — jf4c09477_si_001.pdf [file jf4c09477_si_001.pdf]

Supporting Information

Phytotoxicity Study of (Amino)imidazo[1,2-*a*]pyridine Derivatives Towards the Control of *Bidens pilosa*, *Urochloa decumbens*, and *Panicum maximum* Weeds

Luan A. Martinho,[a] Daniel M. de Lima,[a] Victor H. J. G. Praciano,[a] Sarah Christina C. Oliveira,[b]* Carlos Kleber Z. Andrade[a]*

[a] Instituto de Química, Laboratório de Química Metodológica e Orgânica Sintética (LaQMOS), Universidade de Brasília, 70904-970, Brasília, DF, Brazil

[b] Instituto de Ciências Biológicas, Departamento de Botânica, Laboratório de Alelopatia Alfredo Gui Ferreira, Universidade de Brasília, 70910-900 Brasília, DF, Brazil

*e-mail: ckleber@unb.br

Typical procedures

General Procedure A: aminopyridine (0.5 mmol), cyanobenzaldehyde (0.5 mmol), HPW (2 mol%), isocyanide (0.5 mmol), and EtOH (0.5 mL) were sequentially added to a 2-5 mL vial. The mixture was subjected to microwave irradiation at 120 ºC for 30 minutes. After the reaction was complete, monitored by TLC, the reaction mixture was cooled to room temperature, and then purified by column chromatography (Martinho; Andrade, 2024).

General Procedure B: $ZnCl_2$ (0.5 mmol), the GBB product (0.5 mmol), NaN_3 (1.0 mmol), and EtOH (1.0 mL) were added, respectively, to a 2-5 mL vial. The mixture was heated in the microwave at 130 ºC for 2 hours. After the reaction was complete, the mixture was cooled to room temperature. Then, approximately 3-4 mL of cold water was added to the reaction mixture, followed by 1-2 mL of 2.5 M HCl until a solid precipitated. The solid was then filtered under vacuum and washed with ethanol or cold water.

Spectroscopic data for new compounds

2-(2-Bromophenyl)-*N*-octylimidazo[1,2-*a*]pyridin-3-amine **(4x)**

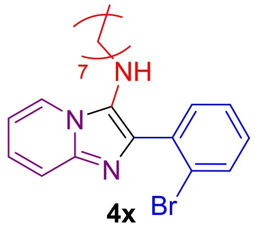

4x

4x was obtained from 2-aminopyridine (0.047 g; 0.50 mmol), 2-bromobenzaldehyde (0.50 mmol; 0.058 mL), HPW (0.029 g; 2 mol%), *n*-octyl isocyanide (0.50 mmol; 0.089 mL) in EtOH (0.50 mL), following procedure **A**, in 62% yield as a brown oil. R_f = 0.43 (30% AcOEt/Hexane).

FT-IR (ATR): 2926, 2854, 1633, 1568, 1433, 1352, 1194, 1024, 750, 627, 600 cm^{-1}.

^{1}H NMR (600 MHz, $CDCl_3$): δ 8.21 (dt, *J* = 6.9, 1.2 Hz, 1H), 7.74 – 7.67 (m, 2H), 7.65 (dd, *J* = 7.5, 1.7 Hz, 1H), 7.44 (td, *J* = 7.5, 1.2 Hz, 1H), 7.33 – 7.24 (m, 2H), 6.93 (td, *J* = 6.9, 1.2 Hz, 1H), 3.42 (t, *J* = 6.4 Hz, 1H), 2.85 (q, *J* = 6.7 Hz, 2H), 1.38 – 1.30 (m, 2H), 1.30 – 1.23 (m, 2H), 1.22 – 1.15 (m, 6H), 1.15 – 1.09 (m, 2H), 0.88 (t, *J* = 7.2 Hz, 3H) ppm.

^{13}C NMR (151 MHz, $CDCl_3$): δ 140.5, 132.8, 132.8, 132.8, 129.9, 127.6, 127.3, 125.0, 123.2, 122.9, 117.1, 112.5, 48.2, 31.8, 30.3, 29.3, 29.2, 26.8, 22.6, 14.1 ppm.

HRMS (ESI-QTOF): *m*/*z* calculated for $C_{21}H_{27}BrN_3^+$, 400.1383 [M+H]$^+$; found: 400.1395.

2-(2-Bromophenyl)-*N*-octadecylimidazo[1,2-*a*]pyridin-3-amine **(4y)**

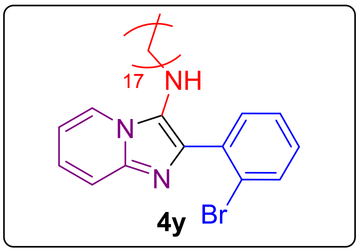

4y was obtained from 2-aminopyridine (0.047 g; 0.50 mmol), 2-bromobenzaldehyde (0.50 mmol; 0.058 mL), HPW (0.029 g; 2 mol%), *n*-octadecyl isocyanide (0.50 mmol; 0.140 g) in EtOH (0.50 mL), following procedure **A**, in 82% yield as an orange oil. R_f = 0.52 (30% AcOEt/Hexane).

FT-IR (ATR): 2918, 2850, 1572, 1502, 1471, 1356, 1232, 1188, 1024, 752, 654, 642, 617 cm^{-1}.

^{1}H NMR (600 MHz, $CDCl_3$): δ 8.17 (d, *J* = 6.8 Hz, 1H), 7.68 (dd, *J* = 8.1, 1.3 Hz, 1H), 7.65 – 7.60 (m, 2H), 7.42 (td, *J* = 7.6, 1.3 Hz, 1H), 7.28 (td, *J* = 7.6, 1.7 Hz, 1H) 7.21 (ddd, *J* = 8.7, 6.8, 1.3 Hz, 1H), 6.88 (t, *J* = 6.8 Hz, 1H), 3.38 (t, *J* = 6.6 Hz, 1H), 2.84 (q, *J* = 6.6 Hz, 2H), 1.37 – 1.08 (m, 32H), 0.90 (t, *J* = 7.0 Hz, 3H) ppm.

^{13}C NMR (151 MHz, $CDCl_3$): δ 140.9, 135.4, 134.9, 132.8, 132.7, 129.7, 127.5, 127.2, 124.3, 123.2, 122.7, 117.4, 112.0, 48.3, 31.9, 30.3, 29.7, 29.7, 29.7, 29.7, 29.6, 29.6, 29.5, 29.4, 29.3, 26.8, 22.7, 14.1 ppm.

HRMS (ESI-QTOF): *m*/*z* calculated for $C_{31}H_{47}BrN_3^+$, 540.2948 $[M+H]^+$; found: 540.2950.

N,2-bis(2-Bromophenyl)imidazo[1,2-*a*]pyridin-3-amine (**4aa**)

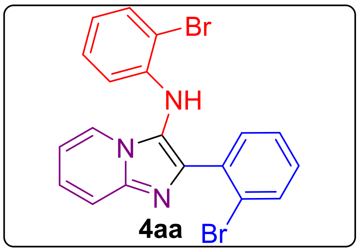

4aa was obtained from 2-aminopyridine (0.047 g; 0.50 mmol), 2-bromobenzaldehyde (0.50 mmol; 0.058 mL), HPW (0.029 g; 2 mol%), 1-bromo-2-isocyanobenzene (0.50 mmol, 0.091 g) in EtOH (0.50 mL), following procedure **A**, in 75% yield as a purplish white solid; m.p. 142-143 ºC. R_f = 0.55 (50% AcOEt/Hexane).

FT-IR (ATR): 3068, 1593, 1487, 1446, 1431, 1390, 1352, 1304, 1284, 1230, 1020, 924, 748, 729, 650 cm^{-1}.

^{1}H NMR (600 MHz, $CDCl_3$): δ 7.82 (dt, *J* = 6.8, 1.2 Hz, 1H), 7.75 (dt, *J* = 9.1, 1.2 Hz, 1H), 7.65 (dd, *J* = 8.1, 1.2 Hz, 1H), 7.61 (dd, *J* = 7.5, 1.7 Hz, 1H), 7.48 (dd, *J* = 8.1, 1.3 Hz, 1H), 7.36 (td, *J* = 7.5, 1.2 Hz, 1H), 7.32 (ddd, *J* = 9.1, 6.7, 1.3 Hz, 1H), 7.23 (ddd, *J* = 8.1, 7.4, 1.5 Hz, 1H), 7.02 – 6.96 (m, 1H), 6.87 (td, *J* = 6.8, 1.2 Hz, 1H), 6.67 (ddd, *J* = 8.1, 7.5, 1.5 Hz, 1H), 6.46 (s, 1H), 6.07 (dd, *J* = 8.1, 1.5 Hz, 1H) ppm.

^{13}C NMR (151 MHz, $CDCl_3$): δ 142.4, 141.5, 139.4, 134.3, 132.9, 132.9, 132.4, 130.0, 128.6, 127.5, 125.4, 123.2, 123.1, 120.6, 118.7, 118.0, 113.2, 112.8, 109.8 ppm.

HRMS (ESI-QTOF): *m*/*z* calculated for $C_{19}H_{14}Br_2N_3^+$, 441.9549 $[M+H]^+$; found: 441.9556.

N-(2-Bromophenyl)-2-cyclohexylimidazo[1,2-*a*]pyridin-3-amine **(4qq)**

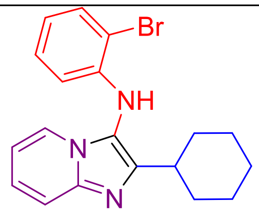

4qq

4qq was obtained from 2-aminopyridine (0.047 g; 0.50 mmol), cyclohexanecarbaldehyde (0.50 mmol, 0.061 mL), HPW (0.029 g; 2 mol%), 1-bromo-2-isocyanobenzene (0.50 mmol, 0.091 g) in EtOH (0.50 mL), following procedure **A**, in 92% yield as a white solid; m.p. 148-149 ºC. R_f = 0.46 (30% AcOEt/Hexane).

FT-IR (ATR): 3263, 2924, 1593, 1572, 1487, 1446, 1348, 1286, 1020, 752, 737, 652, 615 cm^{-1}.

^{1}H NMR (600 MHz, $CDCl_3$): δ 7.77 (dt, *J* = 6.8, 1.2 Hz, 1H), 7.69 (dt, *J* = 9.0, 1.2 Hz, 1H), 7.55 (dd, *J* = 8.0, 1.3 Hz, 1H), 7.25 (ddd, *J* = 9.0, 6.7, 1.3 Hz, 1H), 7.05 (ddd, *J* = 8.4, 7.3, 1.3 Hz, 1H), 6.80 (td, *J* = 6.7, 1.2 Hz, 1H), 6.74 (ddd, *J* = 8.0, 7.3, 1.5 Hz, 1H), 6.13 (dd, *J* = 8.0, 1.5 Hz, 1H), 5.98 (s, 1H), 2.81 (tt, *J* = 11.9, 3.6 Hz, 1H), 1.92 – 1.85 (m, 2H), 1.85 – 1.69 (m, 5H), 1.41 – 1.25 (m, 3H) ppm.

^{13}C NMR (151 MHz, $CDCl_3$): δ 142.4, 142.3, 132.8, 128.8, 125.1, 122.4, 120.6, 117.2, 116.3, 113.1, 112.4, 109.4, 36.5, 32.4, 26.5, 25.9 ppm.

HRMS (ESI-QTOF): *m*/*z* calculated for $C_{19}H_{21}BrN_3^+$, 370.0913 $[M+H]^+$; found: 370.0914.

4-(3-(*tert*-Butylamino)imidazo[1,2-*a*]pyridin-2-yl)benzonitrile **(4rr)**

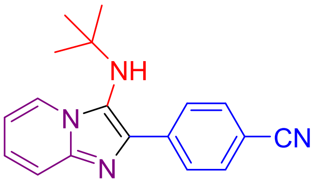

4rr

4rr was obtained from 2-aminopyridine (0.047 g; 0.50 mmol), 4-cyanobenzaldehyde (0.066 g; 0.50 mmol), HPW (0.029 g; 2 mol%), *tert*-butyl isocyanide (0.057 mL; 0.50 mmol) in EtOH (0.50 mL), following procedure **A**, in 92% yield as a white solid; m.p. 158-160 ºC. R_f = 0.27 (40% AcOEt/Hexane).

FT-IR (KBr): 3284, 2966, 2225, 1630, 1606, 1551, 1502, 1443, 1383, 1360, 1329, 1213, 850, 754, 737, 553, 428 cm^{-1}.

^{1}H NMR (600 MHz, DMSO-d_6): δ 8.43 (dt, *J* = 6.8, 1.2 Hz, 1H), 8.41 (dt, *J* = 8.6, 1.6 Hz, 2H), 7.86 (dt, *J* = 8.6, 1.6 Hz, 2H), 7.50 (dt, *J* = 9.0, 1.2 Hz, 1H), 7.23 (ddd, *J* = 9.0, 6.8, 1.2 Hz, 1H), 6.92 (td, *J* = 6.8, 1.2 Hz, 1H), 4.80 (s, 1H), 1.02 (s, 9H) ppm.

^{13}C NMR (151 MHz, DMSO-d_6): δ 141.9, 140.6, 136.3, 132.4, 128.5, 126.1, 125.3, 124.9, 119.6, 117.4, 112.0, 109.5, 56.5, 30.5 ppm.

HRMS (ESI-QTOF): *m*/*z* calculated for $C_{18}H_{19}N_4^+$, 291.1604 $[M+H]^+$; found: 291.1611.

Methyl (2-(4-cyanophenyl)imidazo[1,2-*a*]pyridin-3-yl)glycinate **(4ss)**

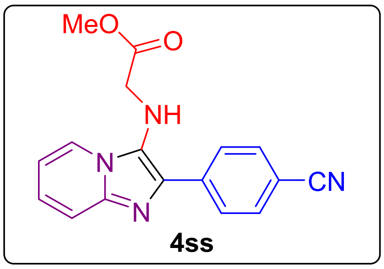

4ss was obtained from 2-aminopyridine (0.047 g; 0.50 mmol), 4-cyanobenzaldehyde (0.066 g; 0.50 mmol), HPW (0.029 g; 2 mol%), methyl isocyanoacetate (0.045 mL; 0.5 mmol) in MeOH (0.50 mL), following procedure **A**, in 78% yield as a brownish oil. R_f = 0.26 (50% AcOEt/Hexane).

FT-IR (KBr): 3196, 2227, 1743, 1682, 1487, 1443, 1385, 1203, 1151, 1020, 991, 847, 775, 739, 553, 521, 413 cm^{-1}.

^{1}H NMR (600 MHz, DMSO-*d*$_6$): δ 8.48 (dt, *J* = 7.0, 1.2 Hz, 1H), 8.35 – 8.30 (m, 2H), 7.91 – 7.86 (m, 2H), 7.49 (dt, *J* = 9.1, 1.2 Hz, 1H), 7.23 (ddd, *J* = 9.1, 6.4, 1.2 Hz, 1H), 6.93 (td, *J* = 7.0, 1.2 Hz, 1H), 5.59 (t, *J* = 6.4 Hz, 1H), 3.85 (d, *J* = 6.4 Hz, 2H) ppm.

^{13}C NMR (151 MHz, DMSO-*d*$_6$): δ 172.5, 160.2, 148.1, 148.1, 141.3, 139.5, 137.4, 132.8, 131.7, 128.5, 127.3, 127.2, 125.2, 124.7, 119.7, 117.4, 112.3, 112.0, 109.3, 108.5, 52.1, 49.0 ppm.

HRMS (ESI-QTOF): *m*/*z* calculated for $C_{17}H_{15}N_4O_2^+$, 307.1190 $[M+H]^+$; found: 307.1188.

4-(3-(Phenylamino)imidazo[1,2-*a*]pyridin-2-yl)benzonitrile **(4tt)**

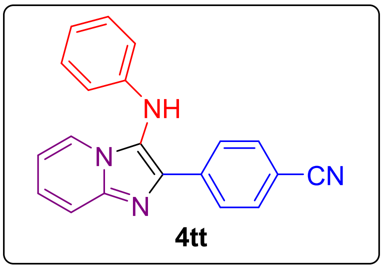

4tt was obtained from 2-aminopyridine (0.047 g; 0.50 mmol), 4-cyanobenzaldehyde (0.066 g; 0.50 mmol), HPW (0.029 g; 2 mol%), phenyl isocyanate (0.053 mL; 0.50 mmol) in EtOH (0.50 mL), following procedure **A**, in 68% yield as a brownish solid; m.p. 210-216 ºC. R_f = 0.21 (40% AcOEt/Hexane).

FT-IR (KBr): 2359, 2227, 1603, 1570, 1489, 1443, 1425, 1385, 1356, 1342, 1308, 1292, 1275, 1238, 1190, 1176, 1149, 1068, 1028, 991, 960, 930, 914, 874, 845, 773, 760, 742, 704, 688, 600, 546, 523, 426, 409 cm^{-1}.

^{1}H NMR (600 MHz, DMSO-*d*$_6$): δ 8.35 (s, 1H), 8.25 – 8.21 (m, 2H), 7.98 (dt, *J* = 6.8, 1.1 Hz, 1H), 7.89 – 7.85 (m, 2H), 7.67 (dt, *J* = 9.1, 1.1 Hz, 1H), 7.36 (ddd, *J* = 9.1, 6.8, 1.1 Hz, 1H), 7.15 (td, *J* = 8.4, 7.3 Hz, 2H), 6.96 (td, *J* = 6.8, 1.1 Hz, 1H), 6.75 (tt, *J* = 7.3, 1.1 Hz, 1H), 6.52 (dd, 2H) ppm.

^{13}C NMR (151 MHz, DMSO-*d*$_6$): δ 145.5, 142.6, 138.7, 135.9, 133.0, 130.1, 127.3, 126.4, 123.8, 121.1, 119.5, 119.4, 117.9, 113.5, 113.2, 110.1 ppm.

HRMS (ESI-QTOF): *m*/*z* calculated for $C_{20}H_{15}N_4^+$, 311.1291 $[M+H]^+$; found: 311.1292.

4-(7-Chloro-3-(cyclohexylamino)imidazo[1,2-*a*]pyridin-2-yl)benzonitrile **(4uu)**

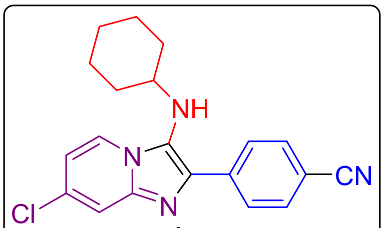

4uu

4uu was obtained from 2-amino-4-chloropyridine (0.064 g; 0.50 mmol), 4-cyanobenzaldehyde (0.066 g; 0.50 mmol), HPW (0.029 g; 2 mol%), cyclohexyl isocyanide (0.062 mL; 0.50 mmol) in EtOH (0.50 mL), following procedure **A**, in 87% yield as a greenish solid. m.p. 225-231 ºC. R_f = 0.50 (50% AcOEt/Hexane).

FT-IR (KBr): 3354, 3082, 2929, 2852, 2225, 2077, 1653, 1610, 1506, 1450, 1425, 1346, 1234, 1201, 1074, 939, 852, 793, 611, 550 cm^{-1}.

^{1}H NMR (600 MHz, DMSO-*d*$_6$): δ 8.59 (d, *J* = 7.3 Hz, 1H), 8.49 (d, *J* = 7.3 Hz, 1H), 8.34 (dd, *J* = 8.3, 5.3 Hz, 2H), 8.19 (d, *J* = 8.3 Hz, 1H), 7.94 (d, *J* = 8.3 Hz, 1H), 7.86 (d, *J* = 2.1 Hz, 1H), 7.77 (d, *J* = 2.1 Hz, 1H), 7.32 (dd, *J* = 7.3, 2.1 Hz, 1H); 7.17 (dd, *J* = 7.3, 2.1 Hz, 2H); 5.36 – 5.14 (m, 1H); 1.80 – 1.71 (m, 2H); 1.66 – 1.60 (m, 2H); 1.31 – 1.21 (m, 2H); 1.13 – 1.05 (m, 3H) ppm.

^{13}C NMR (151 MHz, DMSO-*d*$_6$): δ 140.0; 139.1, 133.0, 128.8, 128.2, 127.9, 127.8, 127.4, 126.1, 125.8, 119.4, 115.9, 115.0, 114.9, 113.9, 110.2, 57.2, 57.1, 33.9, 33.8, 25.7, 25.0 ppm.

HRMS (ESI-QTOF): *m*/*z* calculated for $C_{20}H_{20}ClN_4^+$, 351.1371 $[M+H]^+$, found: 351.1367.

3-(3-(*tert*-Butylamino)imidazo[1,2-*a*]pyridin-2-yl)benzonitrile **(4vv)**

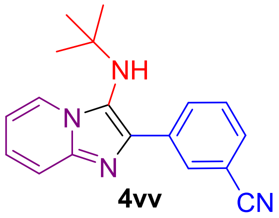

4vv

4vv was obtained from 2-aminopyridine (0.047 g; 0.50 mmol), 3-cyanobenzaldehyde (0.066 g; 0.50 mmol), HPW (0.029 g; 2 mol%), *tert*-butyl isocyanide (0.057 mL; 0.50 mmol) in EtOH (0.50 mL), following procedure **A**, in 43% yield as a yellowish solid. m.p. 155-159 ºC. R_f = 0.30 (40% AcOEt/Hexane).

FT-IR (KBr): 3292, 2962, 2227, 1632, 1603, 1581, 1554, 1504, 1473, 1444, 1371, 1327, 1281, 1213, 1092, 920, 877, 804, 756, 739, 708, 688, 613, 555, 472 cm^{-1}.

^{1}H NMR (600 MHz, DMSO-d_6): δ 8.61 (t, *J* = 1.8 Hz, 1H), 8.49 (dt, 1H), 8.43 (dt, *J* = 6.6, 1.2 Hz, 1H), 7.74 (dt, *J* = 7.7, 1.2 Hz, 1H), 7.64 (t, *J* = 7.7 Hz, 1H), 7.50 (dt, *J* = 9.0, 1.2 Hz, 1H), 7.23 (ddd, *J* = 9.0, 6.6, 1.2 Hz, 1H), 6.92 (td, *J* = 6.6, 1.2 Hz, 1H), 4.82 (s, 1H), 1.01 (s, 9H) ppm.

^{13}C NMR (151 MHz, DMSO-d_6): δ 141.8, 137.1, 136.2, 132.5, 131.2, 130.9, 129.9, 125.3, 125.2, 124.9, 119.4, 117.3, 112.0, 111.6, 56.4, 30.5 ppm.

HRMS (ESI-QTOF): *m*/*z* calculated for $C_{18}H_{19}N_4^+$, 291.1604 [M+H]$^+$; found: 291.1604.

2-(4-(1*H*-Tetrazol-5-yl)phenyl)-*N*-cyclohexylimidazo[1,2-*a*]pyridin-3-amine **(5a)**

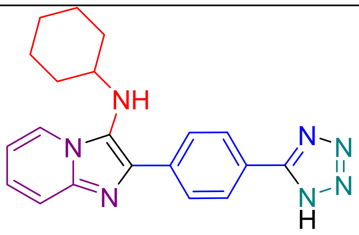

5a

5a was obtained from **4f** product (0.158 g; 0.50 mmol), NaN_3 (0.065 g; 1.00 mmol), $ZnCl_2$ (0.068 g; 0.50 mmol) in EtOH (1.0 mL), following procedure **B**, in 89% yield as a yellowish solid. m.p. 150-154 ºC. R_f = 0.38 (50% AcOEt/Hexane).

FT-IR (KBr): 3271, 3070, 2927, 2852, 1653, 1612, 1525, 1448, 1427, 1390, 1367, 1308, 1234, 1201, 1149, 1086, 1011, 854, 758, 532, 432 cm^{-1}.

^{1}H NMR (600 MHz, DMSO-d_6): δ 8.64 (d, *J* = 6.8 Hz, 1H), 8.38 (d, *J* = 8.2 Hz, 2H), 8.23 (d, *J* = 8.2 Hz, 2H), 7.77 (d, *J* = 9.0 Hz, 1H), 7.66 (t, *J* = 8.2 Hz, 1H), 7.29 (t, *J* = 6.8 Hz, 1H), 5.35 (s, 1H), 1.78 (d, *J* = 12.7 Hz, 2H), 1.63 (q, *J* = 5.2 Hz, 2H), 1.49 (s, 1H), 1.34 – 1.24 (m, 2H), 1.13 – 1.06 (m, 3H) ppm.

^{13}C NMR (151 MHz, DMSO-d_6): δ 155.7, 141.0, 137.6, 133.4, 127.5, 127.5, 127.4, 125.2, 124.1, 122.8, 117.1, 112.4, 57.2, 34.0, 25.9, 25.0 ppm.

HRMS (ESI-QTOF): *m*/*z* calculated for $C_{20}H_{22}N_7^+$, 360.1931 [M+H]$^+$; found: 360.1928.

2-(4-(1*H*-Tetrazol-5-yl)phenyl)-*N*-(*tert*-butyl)imidazo[1,2-*a*]pyridin-3-amine **(5b)**

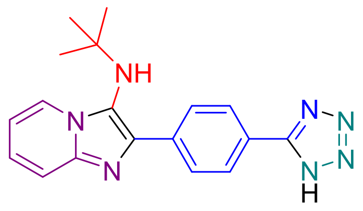

5b

5b was obtained from product **4rr** (0.145 g; 0.50 mmol), NaN_3 (0.065 g; 1.00 mmol), $ZnCl_2$ (0.068; 0.50 mmol) in EtOH (1.0 mL), following procedure **B**, in 96% yield as a white solid. m.p. 234-236 ºC. R_f = 0.35 (50% AcOEt/Hexane).

FT-IR (KBr): 3267, 2968, 2727, 1653, 1618, 1529, 1504, 1433, 1392, 1367, 1203, 1147, 1065, 995, 912, 852, 756, 528 cm^{-1}.

^{1}H NMR (600 MHz, DMSO-*d*$_6$): δ 8.90 (dt, *J* = 6.8, 1.2 Hz, 1H), 8.37 – 8.27 (m, 4H), 7.94 (dd, *J* = 4.0, 1.0 Hz, 2H), 7.50 (dt, *J* = 6.8, 4.0 Hz, 1H), 5.34 (s, 1H), 1.04 (s, 9H) ppm.

^{13}C NMR (151 MHz, DMSO-*d*$_6$): δ 137.9, 133.6, 131.1, 129.5, 128.8, 127.8, 126.6, 126.4, 125.4, 116.8, 112.7, 57.0, 30.2 ppm.

HRMS (ESI-QTOF): *m*/*z* calculated for $C_{18}H_{20}N_7^+$, 334.1775 [M+H]$^+$; found: 334.1776.

2-(4-(1*H*-Tetrazol-5-yl)phenyl)-*N*-phenylimidazo[1,2-*a*]pyridin-3-amine **(5c)**

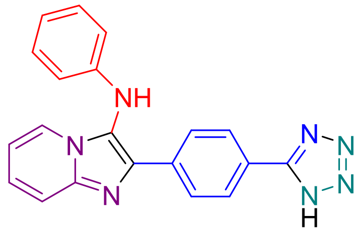

5c

5c was obtained from product **4tt** (0.155 g; 0.50 mmol), NaN_3 (0.065 g; 1.00 mmol), $ZnCl_2$ (0.068; 0.50 mmol) in EtOH (1.0 mL), following procedure **B**, in 72% yield as a yellowish solid. m.p. 158-160 ºC. R_f = 0.37 (50% AcOEt/Hexane).

FT-IR (KBr): 3365, 3194, 1659, 1601, 1498, 1433, 1315, 1250, 1149, 1078, 995, 849, 750, 692, 525 cm^{-1}.

^{1}H NMR (600 MHz, DMSO-*d*$_6$): δ 8.44 (s, 1H), 8.23 – 8.20 (m, 2H), 8.04 (d, *J* = 6.9 Hz, 1H), 7.91 – 7.87 (m, 2H), 7.72 (d, *J* = 9.0 Hz, 1H), 7.44 (t, *J* = 6.9 Hz, 1H), 7.16 (t, 2H), 7.03 (t, *J* = 6.9 Hz, 1H), 6.76 (t, *J* = 7.9 Hz, 1H), 6.55 (d, *J* = 7.9 Hz, 2H) ppm.

^{13}C NMR (151 MHz, DMSO-*d*$_6$): δ 145.3, 142.1, 137.8, 133.1, 130.1, 127.8, 127.7, 127.3, 124.1, 121.3, 119.5, 119.4, 117.3, 113.8, 113.6, 110.4 ppm.

HRMS (ESI-QTOF): *m*/*z* calculated for $C_{20}H_{16}N_7^+$, 354.1462 [M+H]$^+$; found: 354.1462.

2-(4-(1*H*-Tetrazol-5-yl)phenyl)-7-chloro-*N*-cyclohexylimidazo[1,2-*a*]pyridin-3-amine **(5d)**

5d was obtained from product **4uu** (0.175 g; 0.50 mmol), NaN_3 (0.065 g; 1.00 mmol), $ZnCl_2$ (0.068; 0.50 mmol) in EtOH (1.0 mL), following procedure **B**, in 68% yield as a greenish solid. m.p. 264-267 ºC. R_f = 0.55 (90% AcOEt/Hexane).

^{1}H NMR (600 MHz, DMSO-*d*$_6$): δ 8.64 (d, *J* = 7.2 Hz, 1H), 8.36 (d, *J* = 8.1 Hz, 3H), 8.23 (d, *J* = 8.1 Hz, 3H), 7.91 (s, 1H), 7.37 (s, 1H), 1.79 (d, *J* = 12.3 Hz, 3H), 1.64 (s, 2H), 1.50 (s, 1H), 1.31 – 1.25 (m, 3H), 1.10 (s, 3H) ppm.

^{13}C NMR (151 MHz, DMSO-*d*$_6$): δ 128.3, 127.9, 127.9, 126.3, 57.1, 33.8, 25.7, 25.0 ppm.

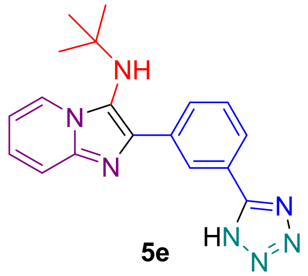

5e

HRMS (ESI-QTOF): *m/z* calculated for $C_{20}H_{21}ClN_7^+$, 394.1541 [M+H]$^+$; found: 394.1549.

2-(3-(1*H*-Tetrazol-5-yl)phenyl)-*N*-(*tert*-butyl)imidazo[1,2-*a*]pyridin-3-amine **(5e)**

5e was obtained from product **4vv** (0.145 g; 0.50 mmol), NaN_3 (0.065 g; 1.00 mmol), $ZnCl_2$ (0.068; 0.50 mmol) in EtOH (1.0 mL), following procedure **B**, in 27% yield as a white solid. m.p. 258-263 ºC. R_f = 0.38 (50% AcOEt/Hexane). The presence of a mixture of two rotamers was observed in the ^{1}H and ^{13}C NMR spectrum.

FT-IR (KBr): 3406, 3298, 2966, 2870, 2789, 2725, 2079, 1657, 1529, 1497, 1444, 1367, 1200, 1068, 1018, 916, 800, 762, 690, 455 cm^{-1}.

^{1}H NMR (600 MHz, DMSO-*d*$_6$): δ 8.89 (t, *J* = 1.8 Hz, 0.64H), 8.71 (dt, *J* = 6.8, 1.2 Hz, 0.64H), 8.61 (dt, *J* = 6.8, 1.2 Hz, 0.36H), 8.57 (t, *J* = 1.5 Hz, 0.36H), 8.43 (dt, *J* = 7.8, 1.5 Hz, 0.36H), 8.34 (dt, *J* = 7.8, 1.5 Hz, 0.64H), 8.09 (dt, *J* = 7.8, 1.5 Hz, 0.64H), 7.84 (dt, *J* = 7.8, 1.5 Hz, 0.36H), 7.78 – 7.62 (m, 2.64H), 7.52 (ddd, *J* = 9.0, 6.8, 1.2 Hz, 0.36H), 7.28 (td, *J* = 6.8, 1.2 Hz, 0.64H), 7.16 (td, *J* = 6.8, 1.2 Hz, 0.36H), 5.09 (s, 0.64H), 5.01 (s, 0.36H), 1.03 (s, 5,76 H), 1.00 (s, 3,24 H) ppm.

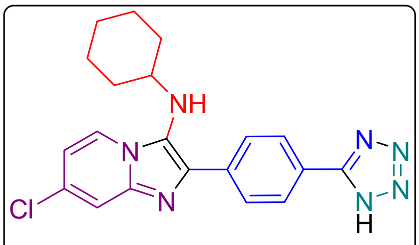

5d

^{13}C NMR (151 MHz, DMSO-*d*$_6$): δ 156.1, 140.2, 139.4, 134.2, 132.8, 132.6, 132.1, 132.0, 131.6, 130.8, 130.2, 130.1, 128.7, 127.3, 126.9, 125.9, 125.8, 125.6, 125.1, 119.1, 115.5, 114.9, 114.5, 114.0, 111.9, 56.7, 56.6, 30.3 ppm.

HRMS (ESI-QTOF): *m/z* calculated for $C_{18}H_{20}N_7^+$, 334.1775 [M+H]$^+$; found: 334.1775.

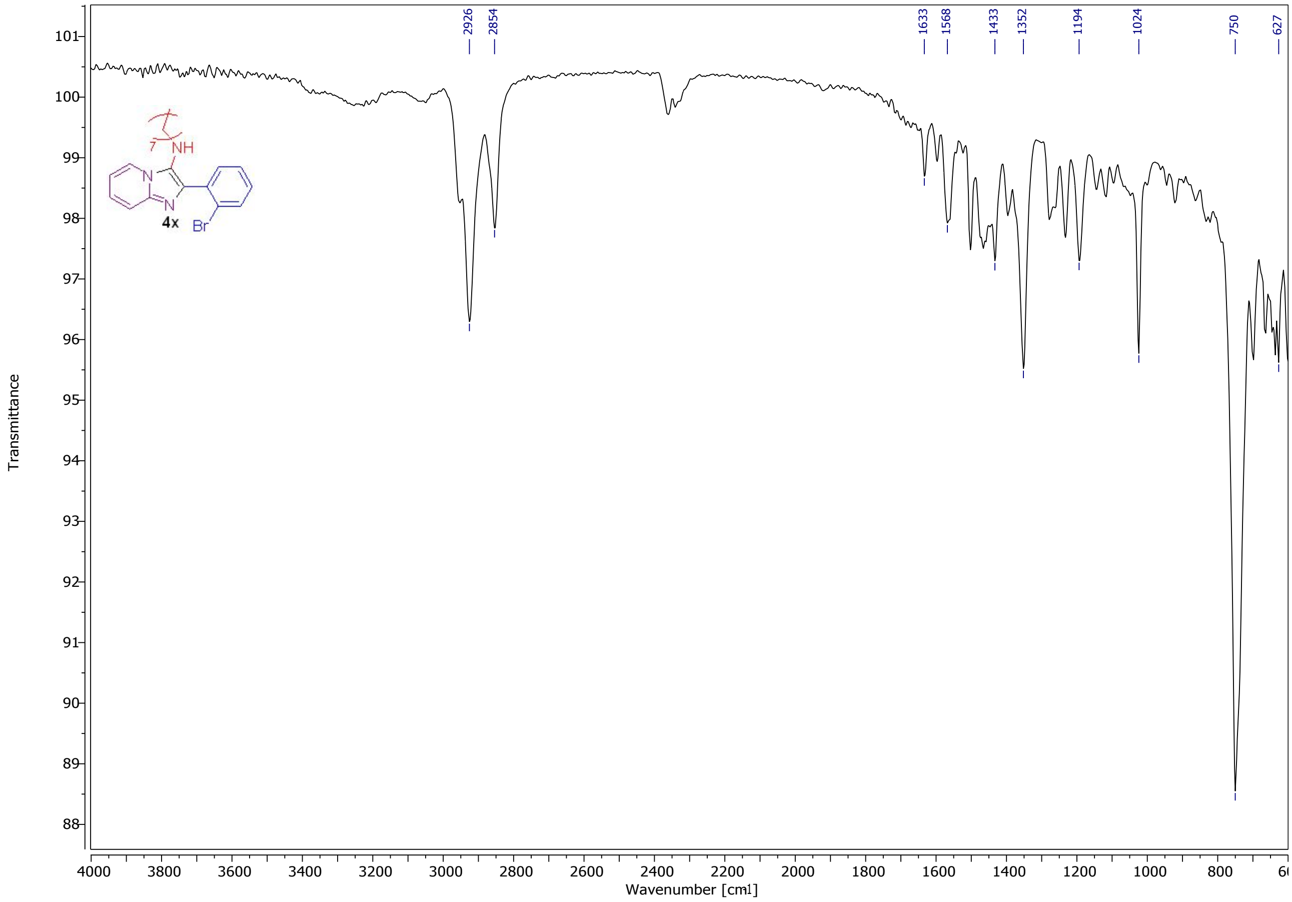

Figure S1. FT-IR (ATR) of compound **4x**.

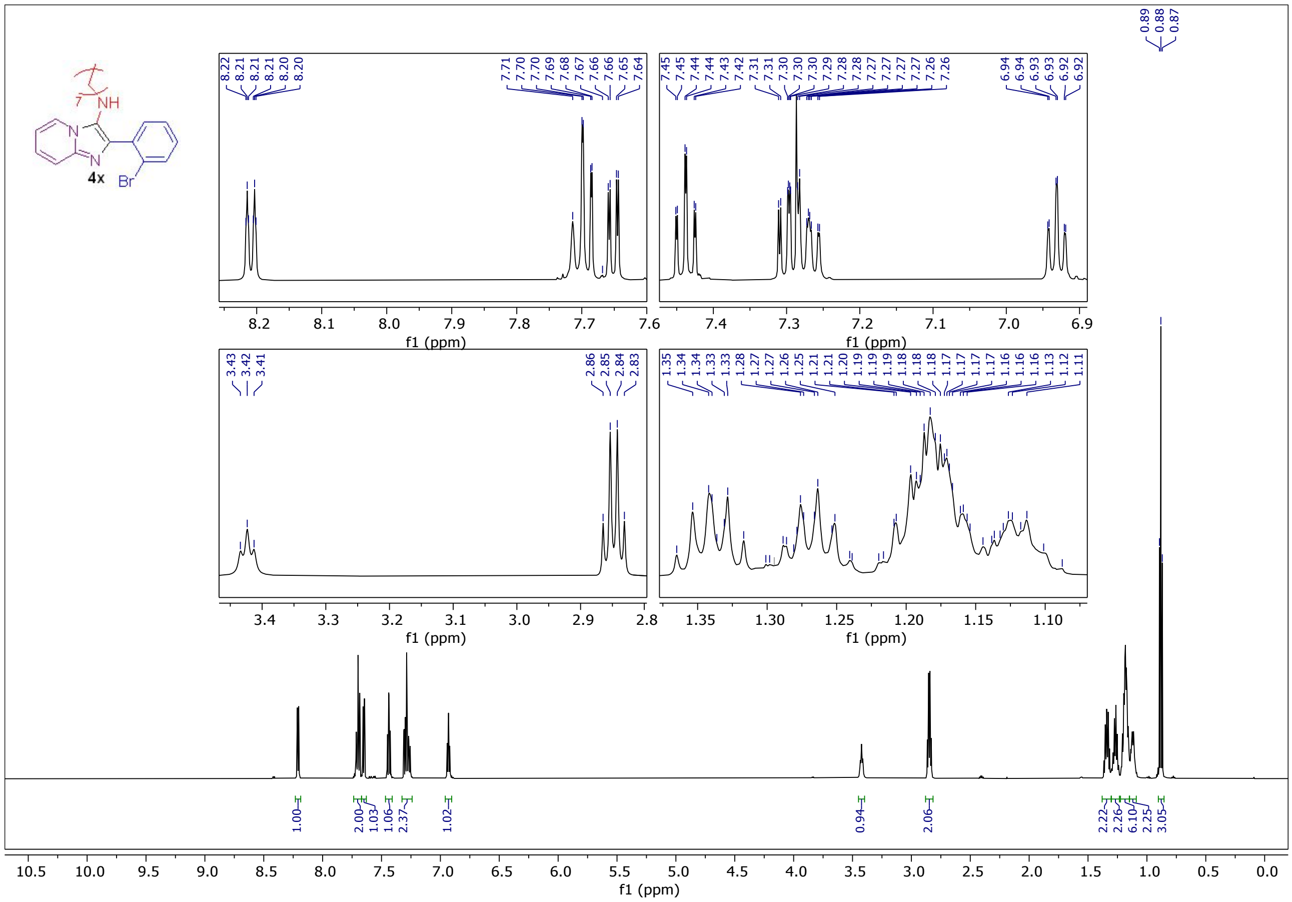

Figure S2. ^{1}H NMR spectrum (600 MHz, $CDCl_3$) of compound **4x**.

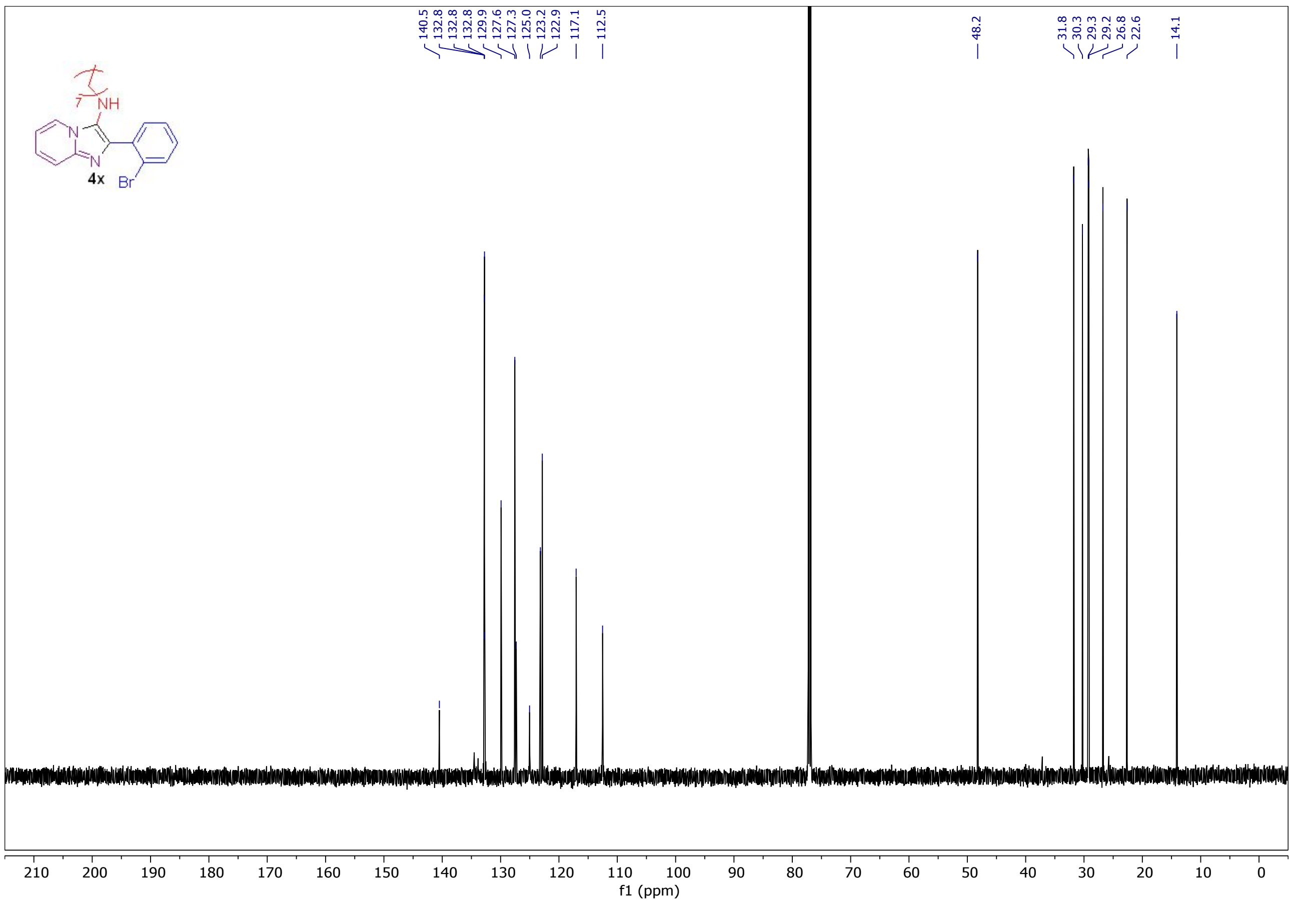

Figure S3. ^{13}C NMR spectrum (151 MHz, $CDCl_3$) of compound **4x**.

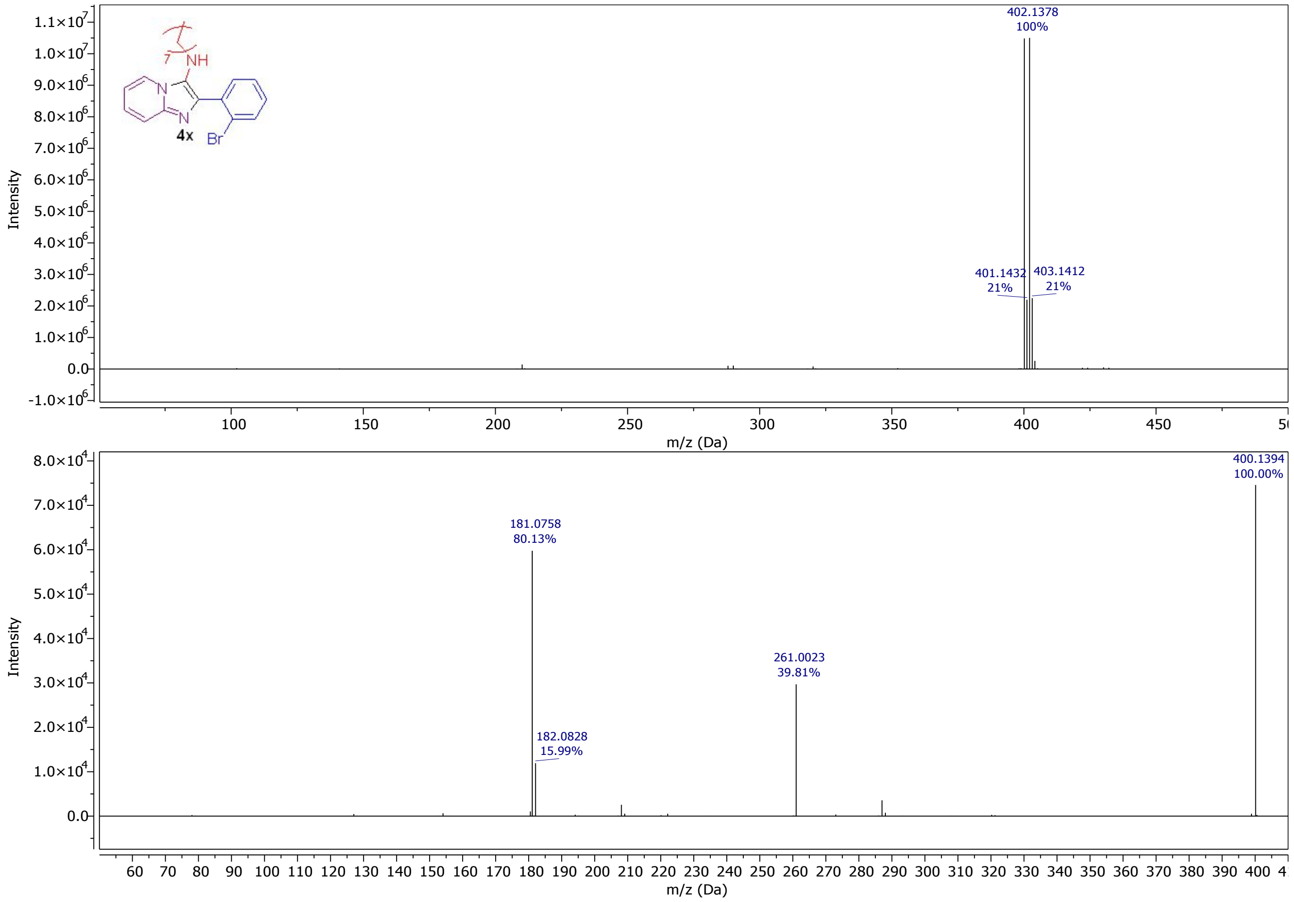

Figure S4. HRMS (ESI-QTOF) of compound **4x** and HRMS/MS for $[M+H]^+$.

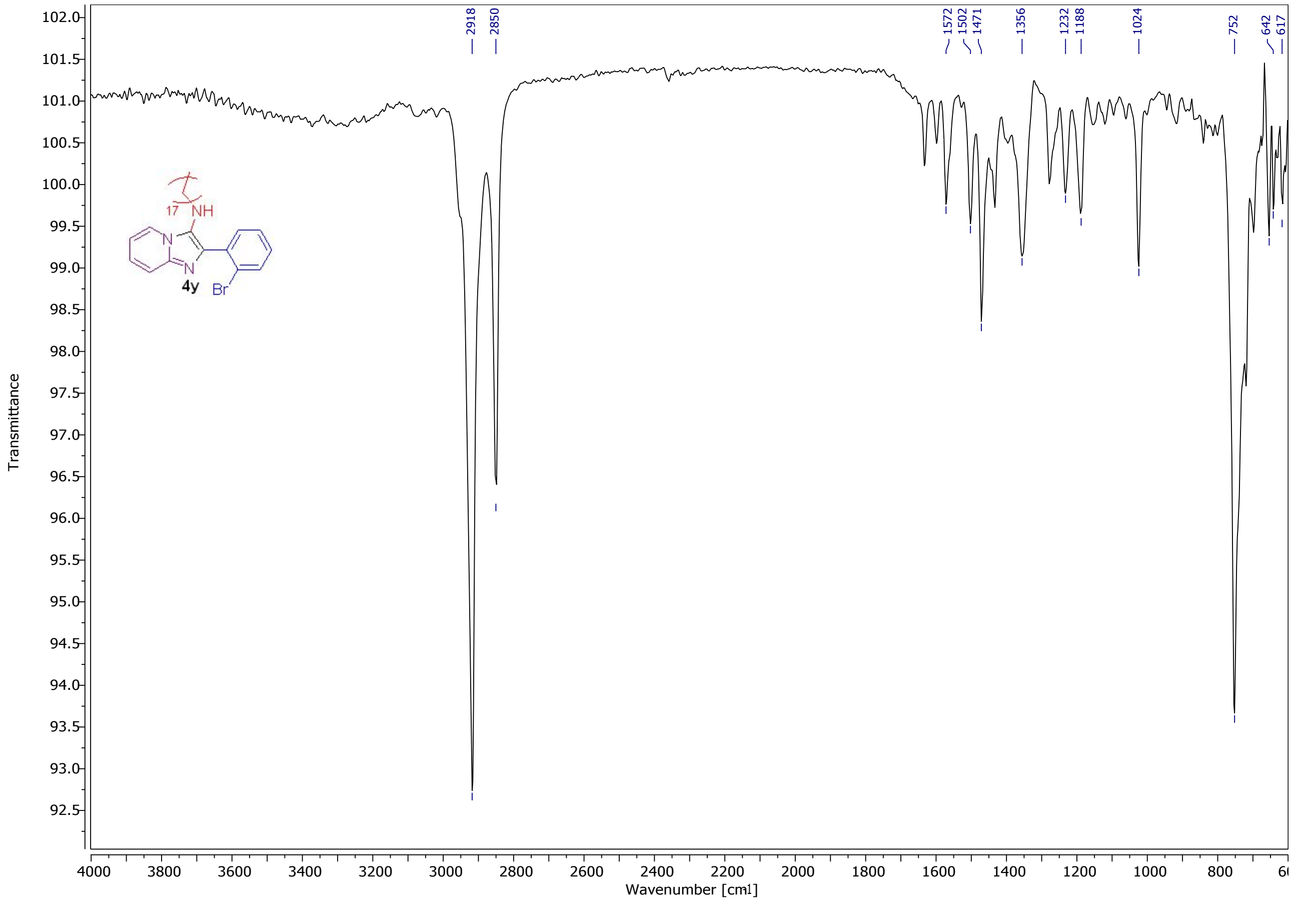

Figure S5. FT-IR (ATR) of compound **4y**.

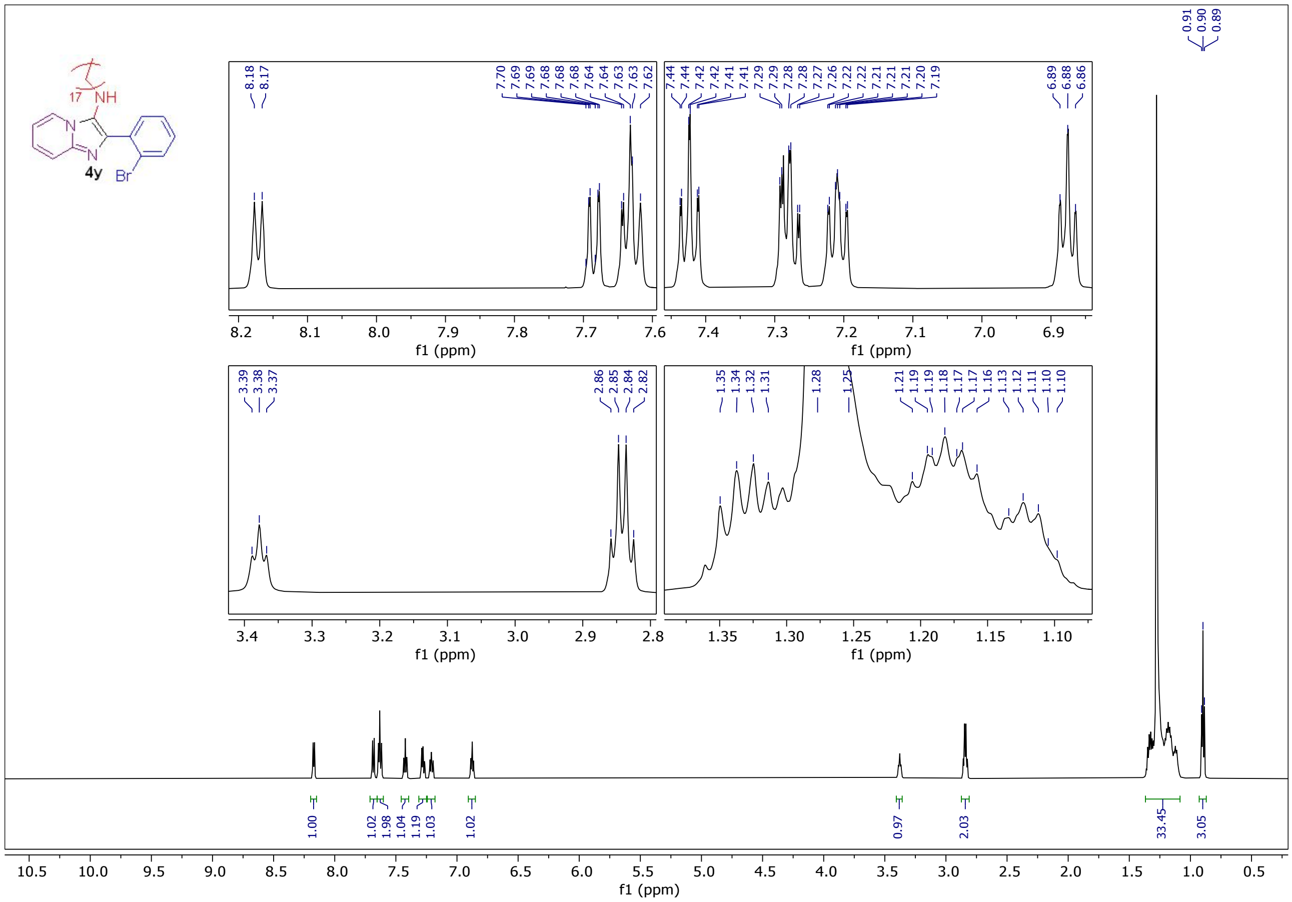

Figure S6. ^{1}H NMR spectrum (600 MHz, $CDCl_3$) of compound **4y**.

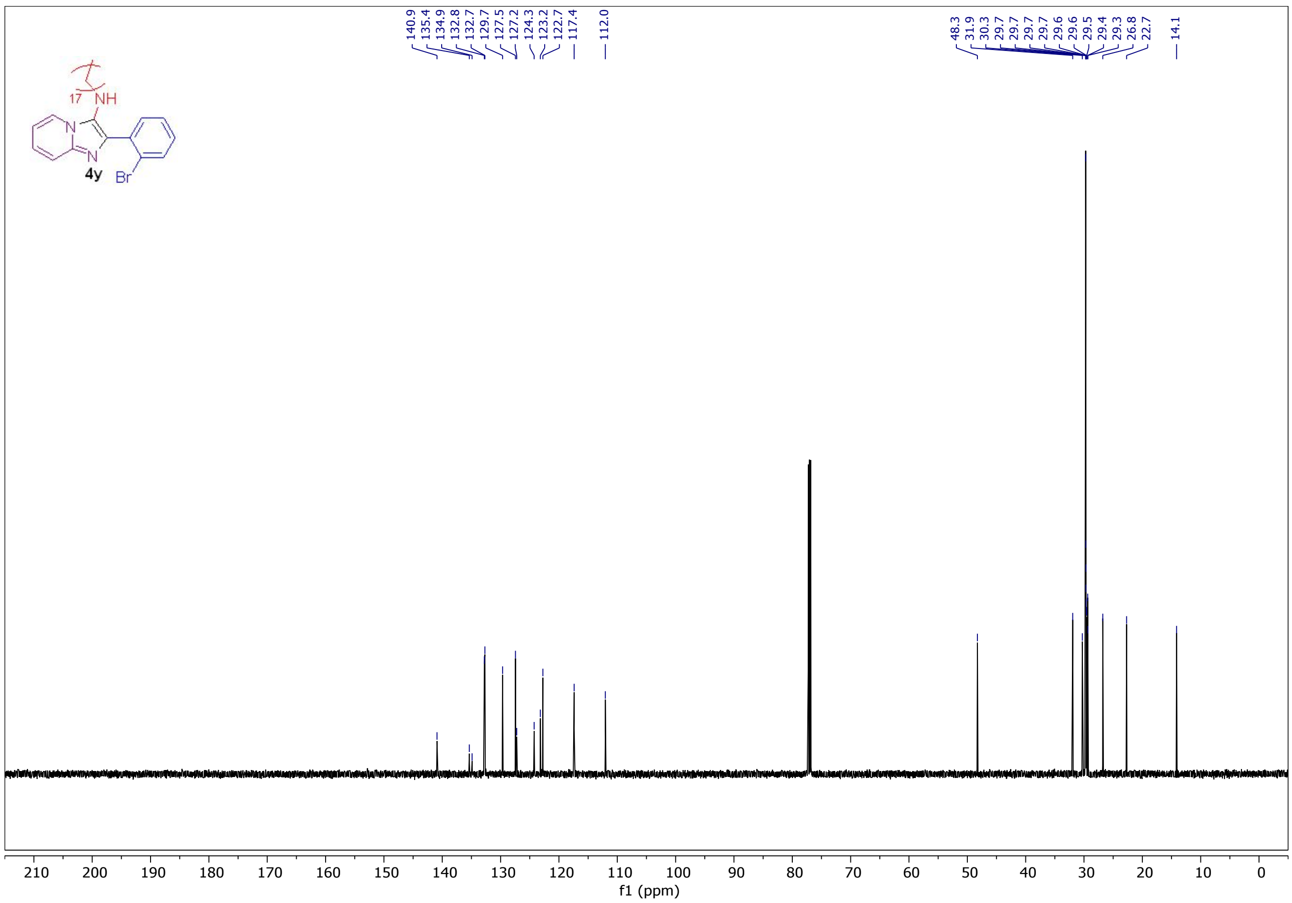

Figure S7. ^{13}C NMR spectrum (151 MHz, $CDCl_3$) of compound **4y**.

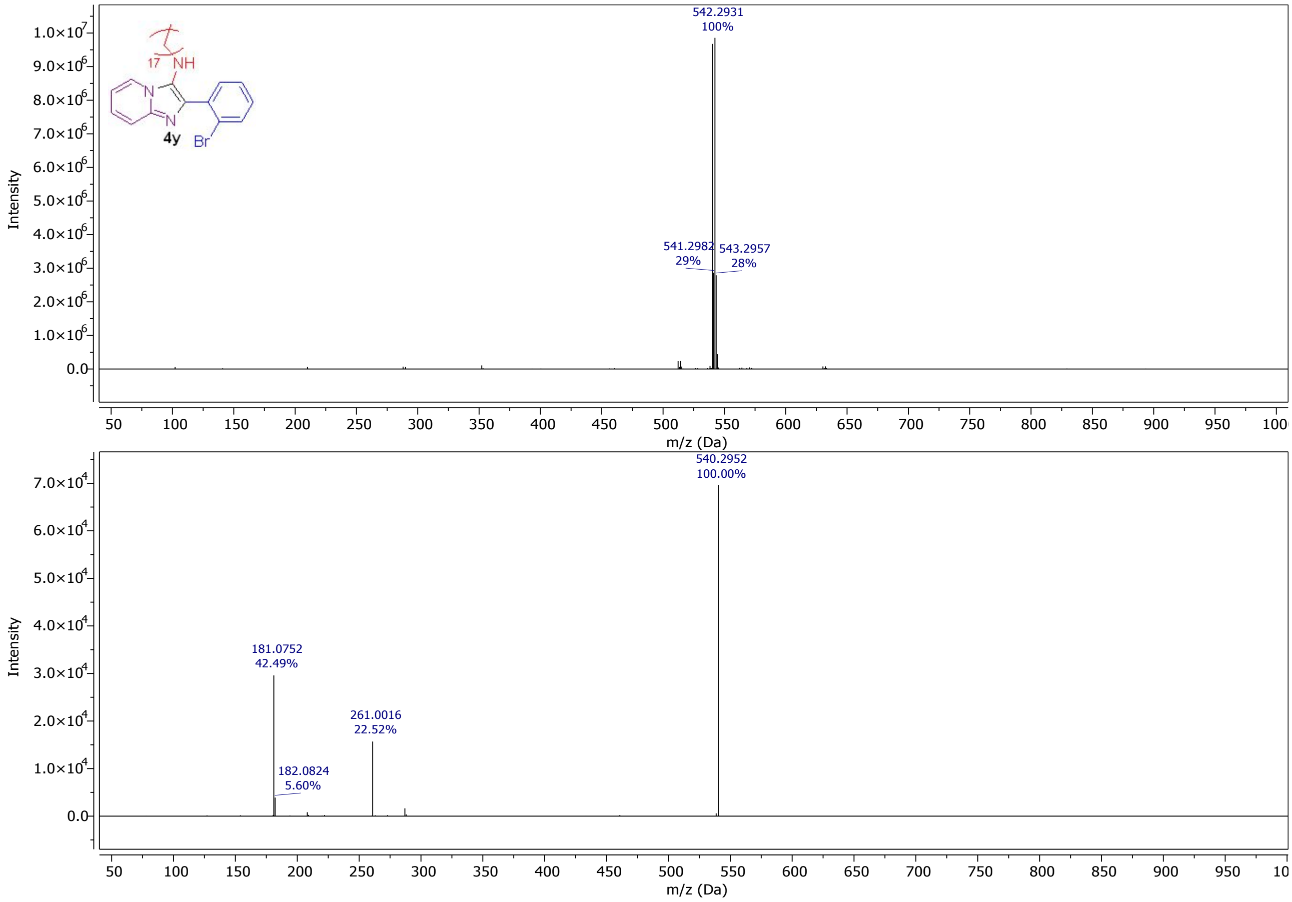

Figure S8. HRMS (ESI-QTOF) of compound **4y** and HRMS/MS for $[M+H]^+$.

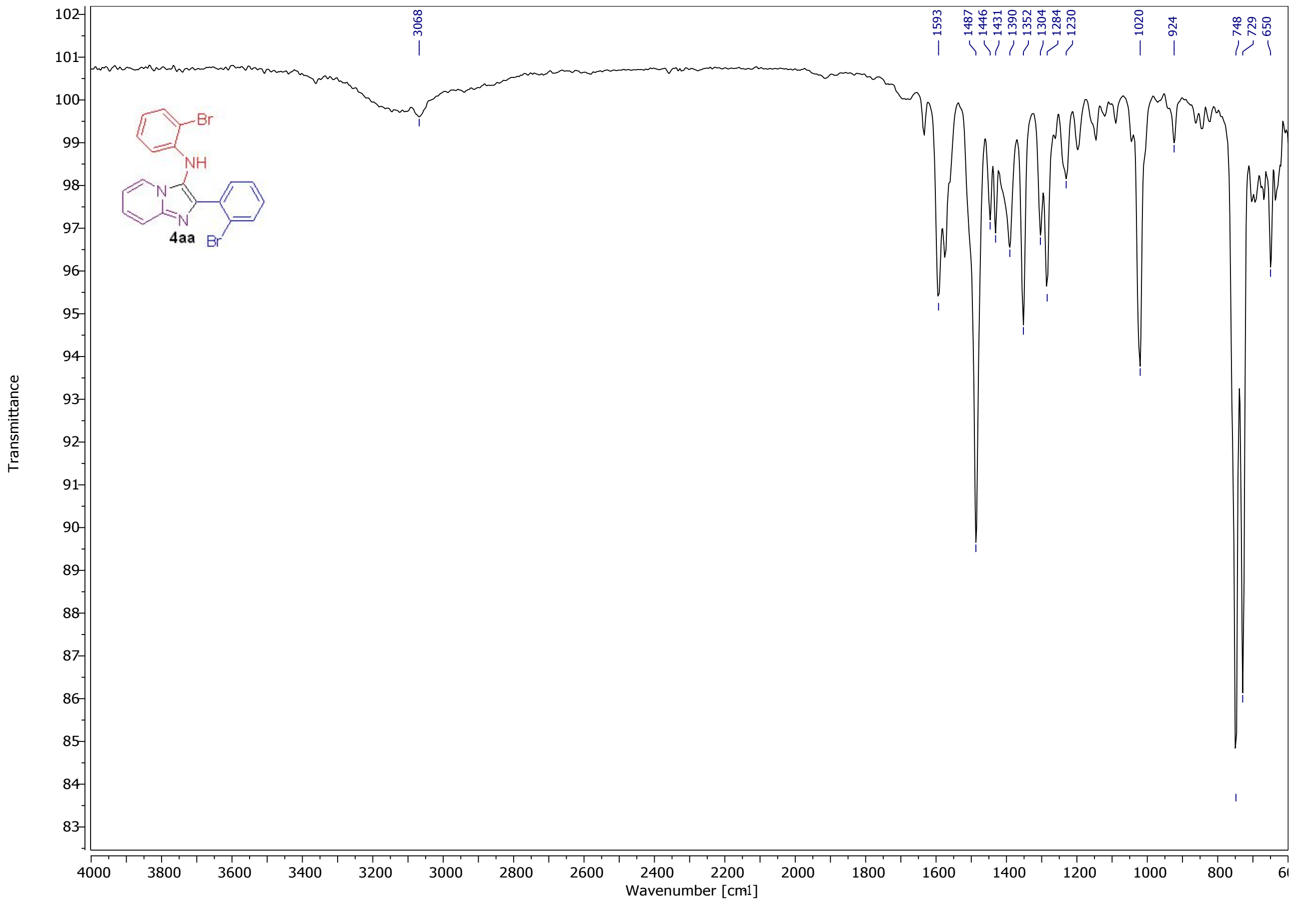

Figure S9. FT-IR (ATR) of compound **4aa**.

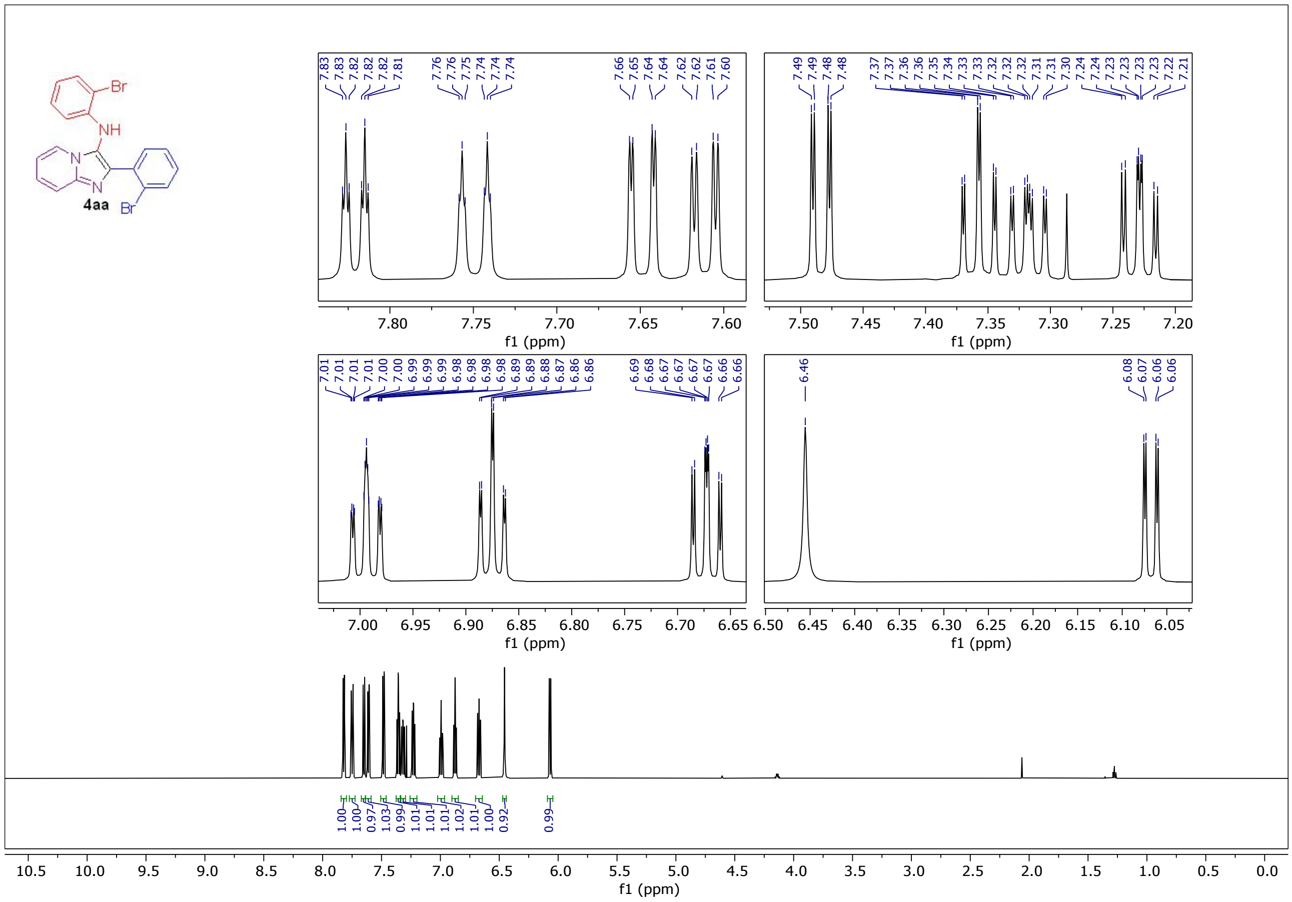

Figure S20. ^1H NMR spectrum (600 MHz, $CDCl_3$) of compound **4aa**.

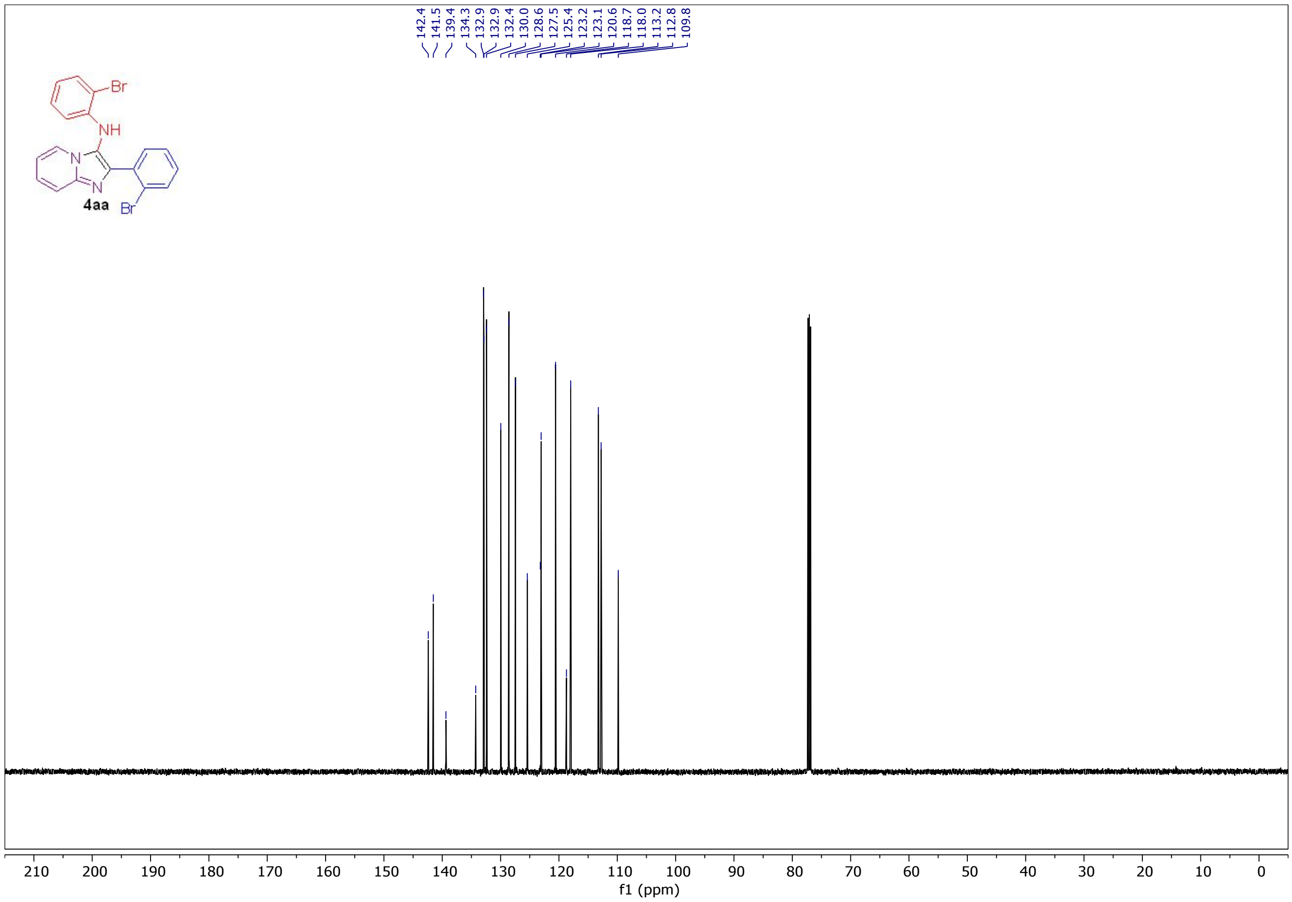

Figure S31. ^{13}C NMR spectrum (151 MHz, $CDCl_3$) of compound **4aa**.

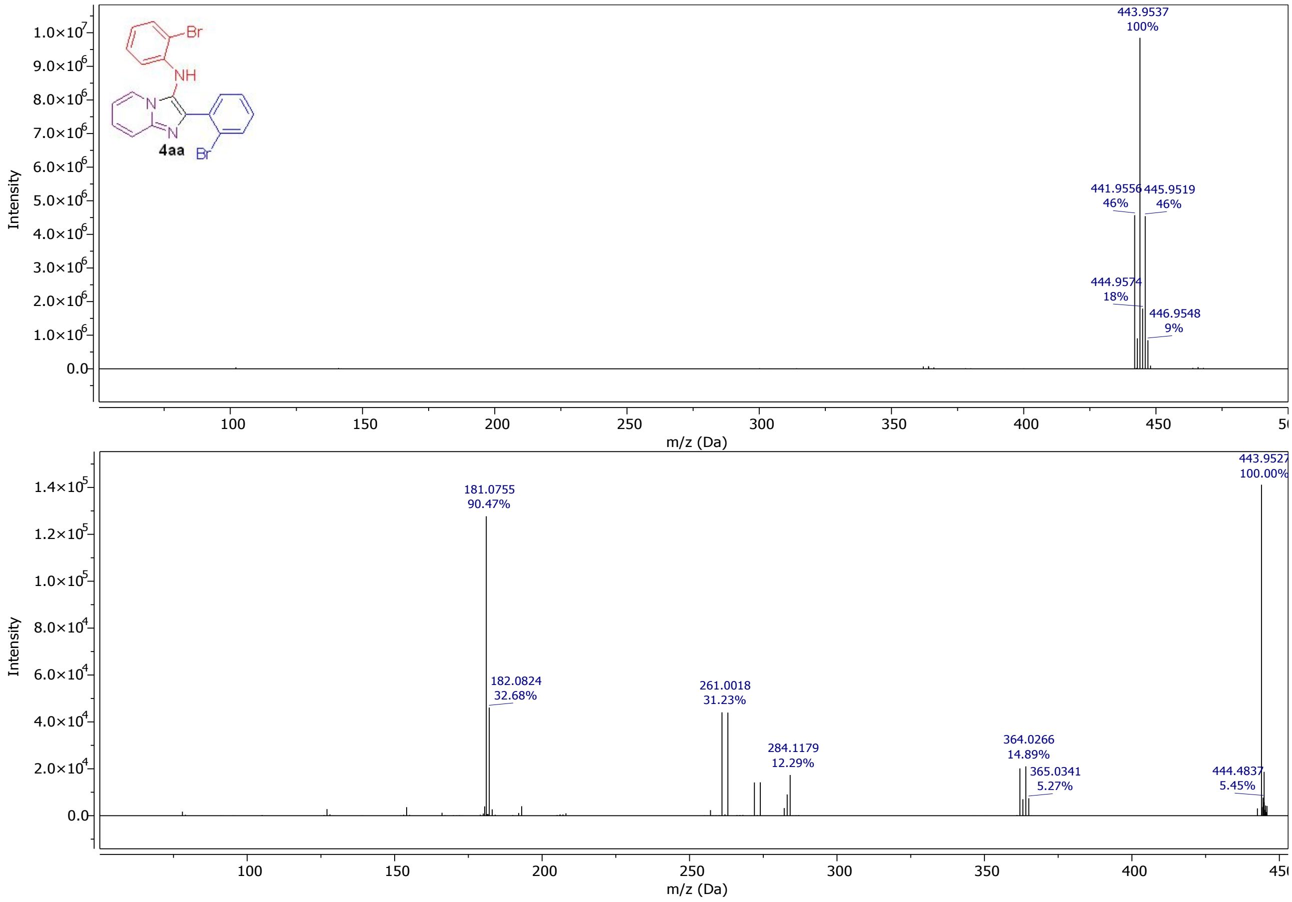

Figure S42. HRMS (ESI-QTOF) of compound **4aa** and HRMS/MS for $[M+H]^+$.

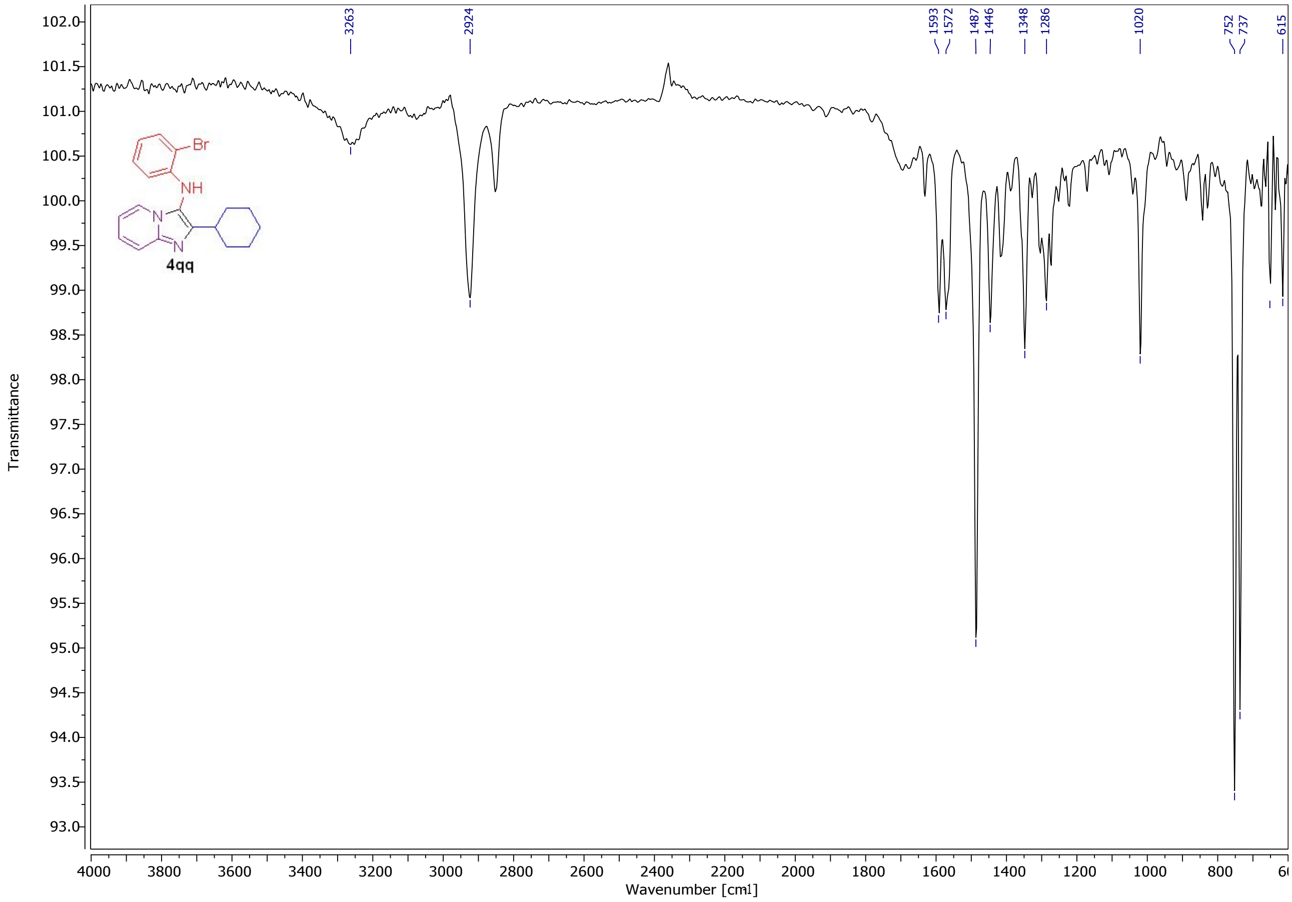

Figure S53. FT-IR (ATR) of compound **4qq**.

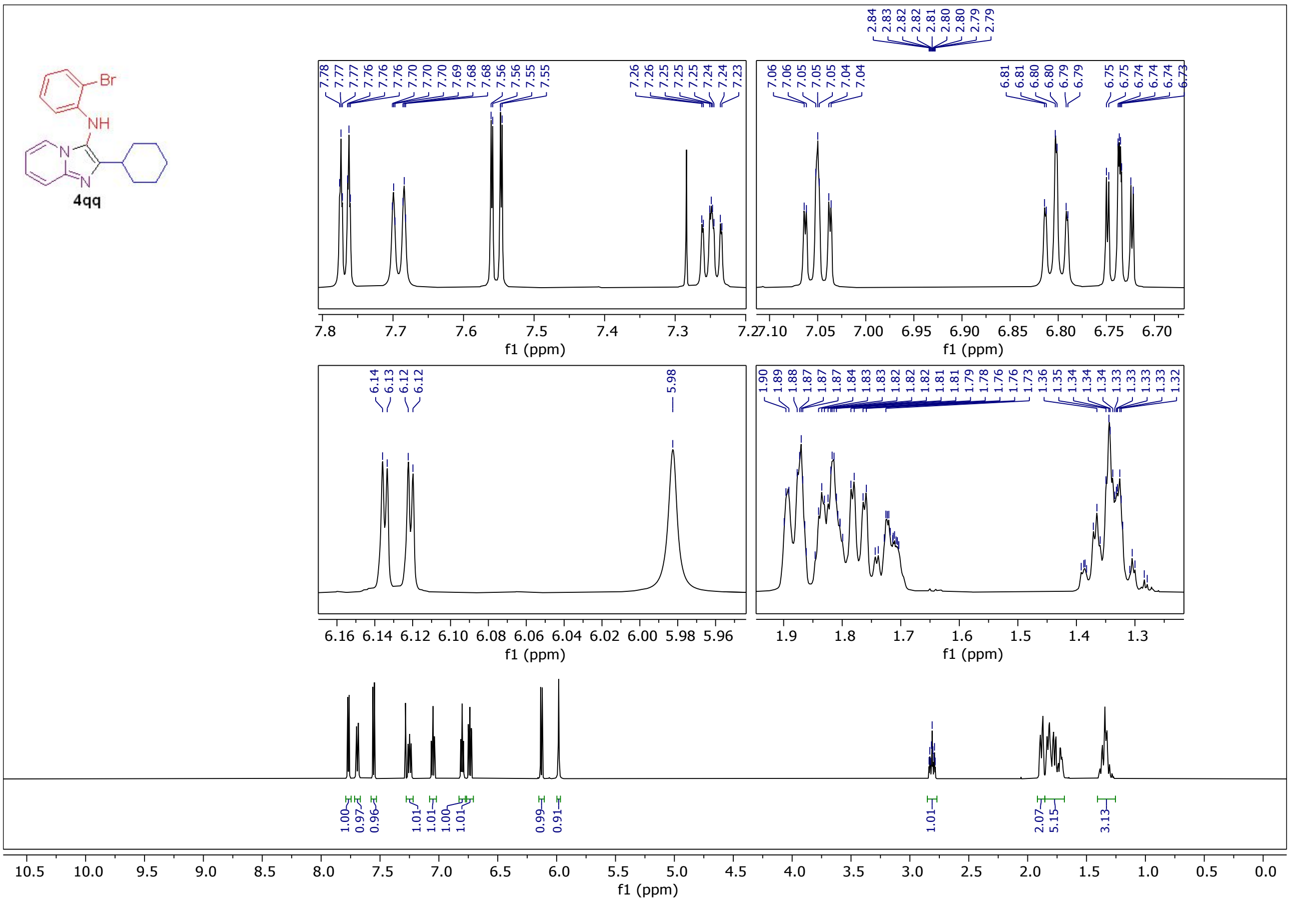

Figure S64. ^{1}H NMR spectrum (600 MHz, $CDCl_3$) of compound **4qq**.

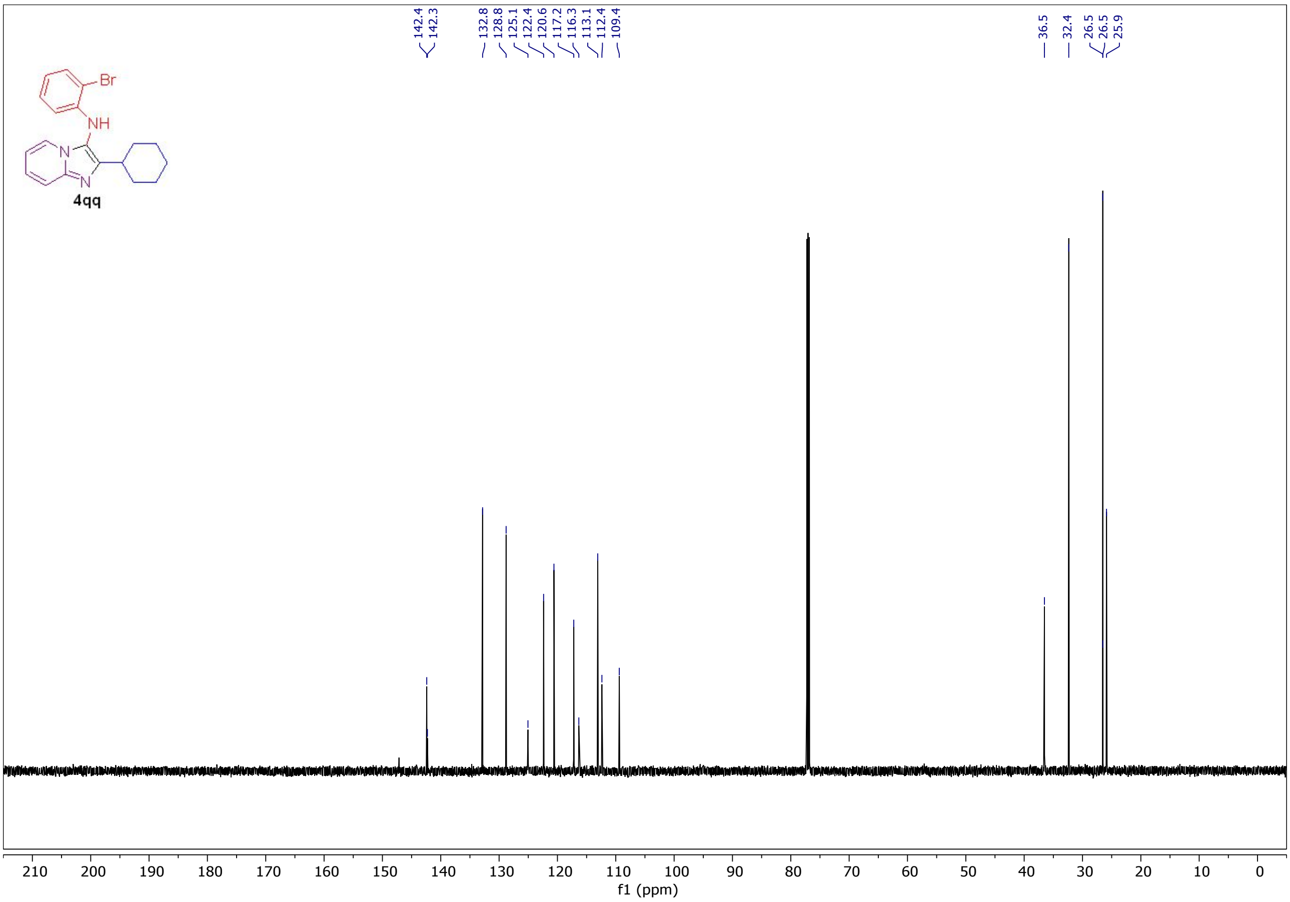

Figure S75. ^{13}C NMR spectrum (151 MHz, $CDCl_3$) of compound **4qq**.

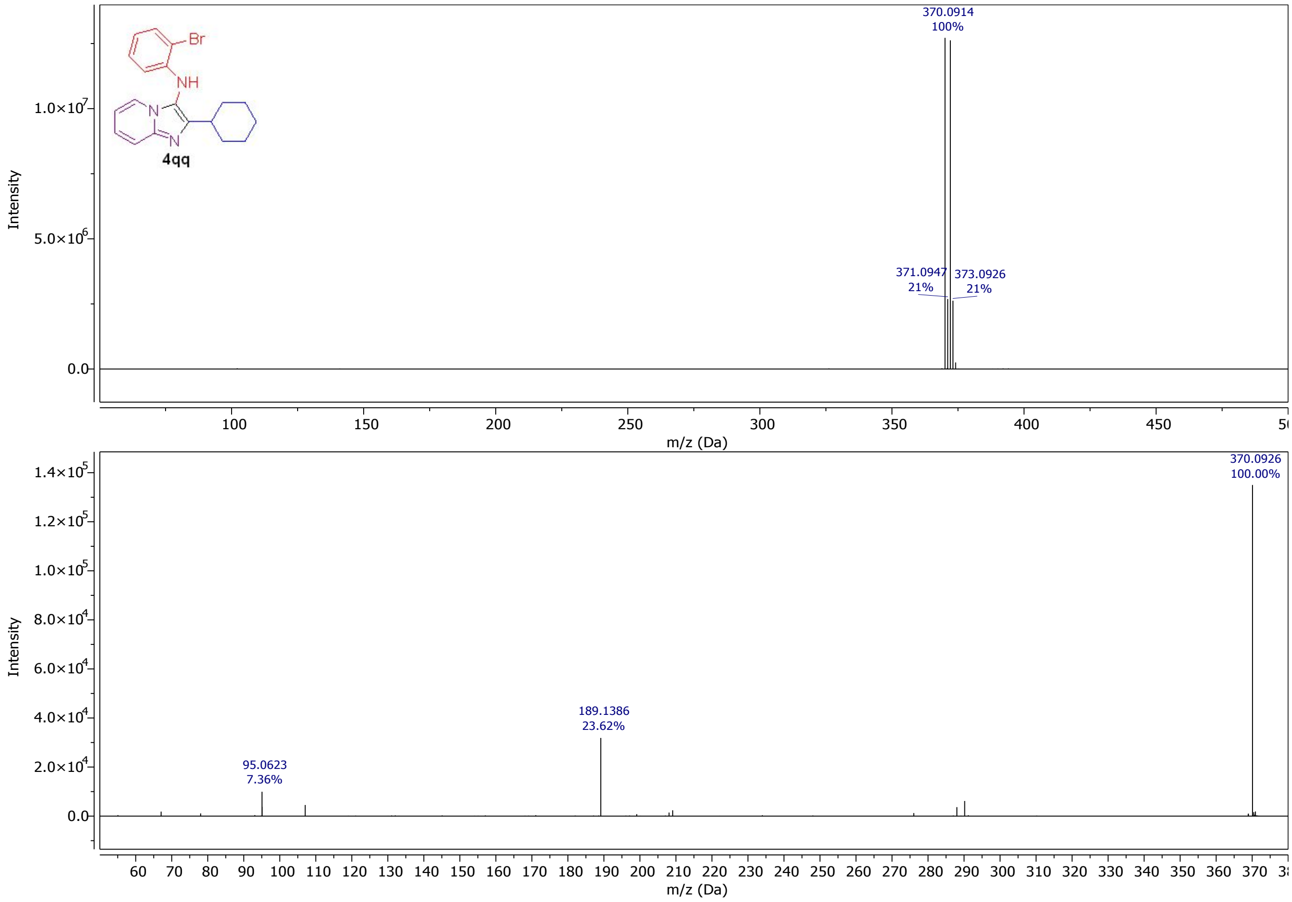

Figure S86. HRMS (ESI-QTOF) of compound **4qq** and HRMS/MS for $[M+H]^+$.

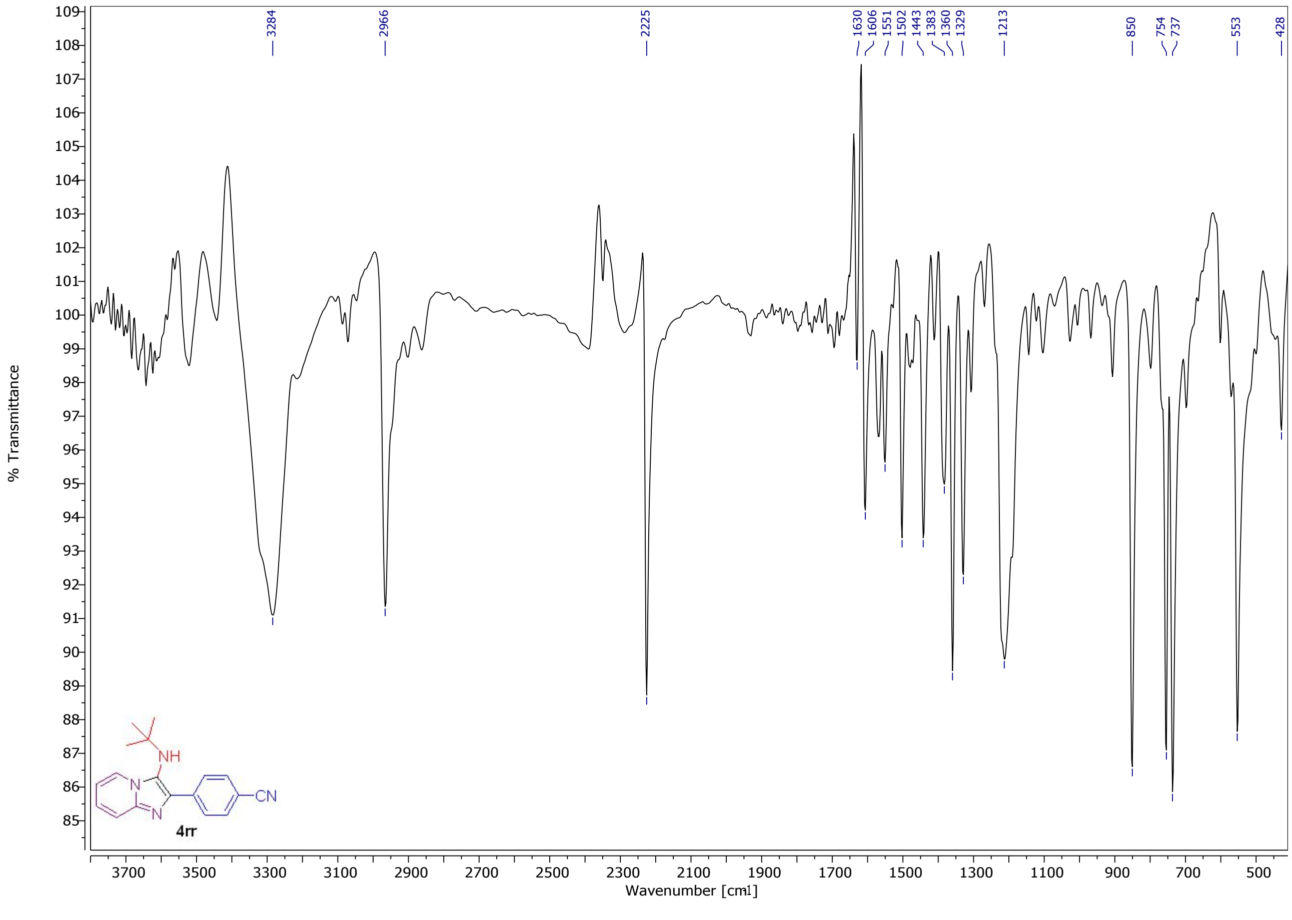

Figure S97. FT-IR (KBr) of compound **4rr**.

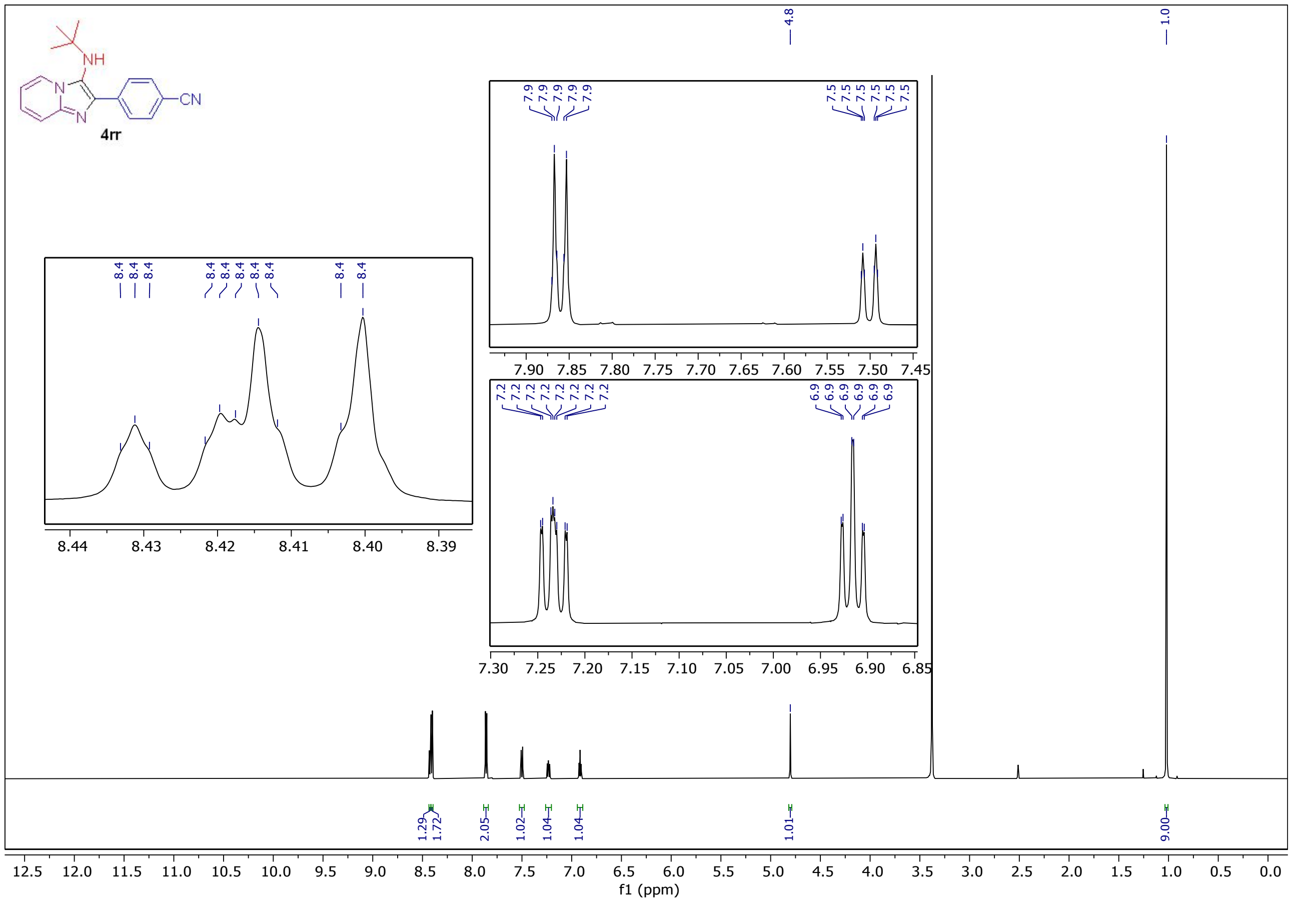

Figure S108. ^{1}H NMR spectrum (600 MHz, DMSO-d_6) of compound **4rr**.

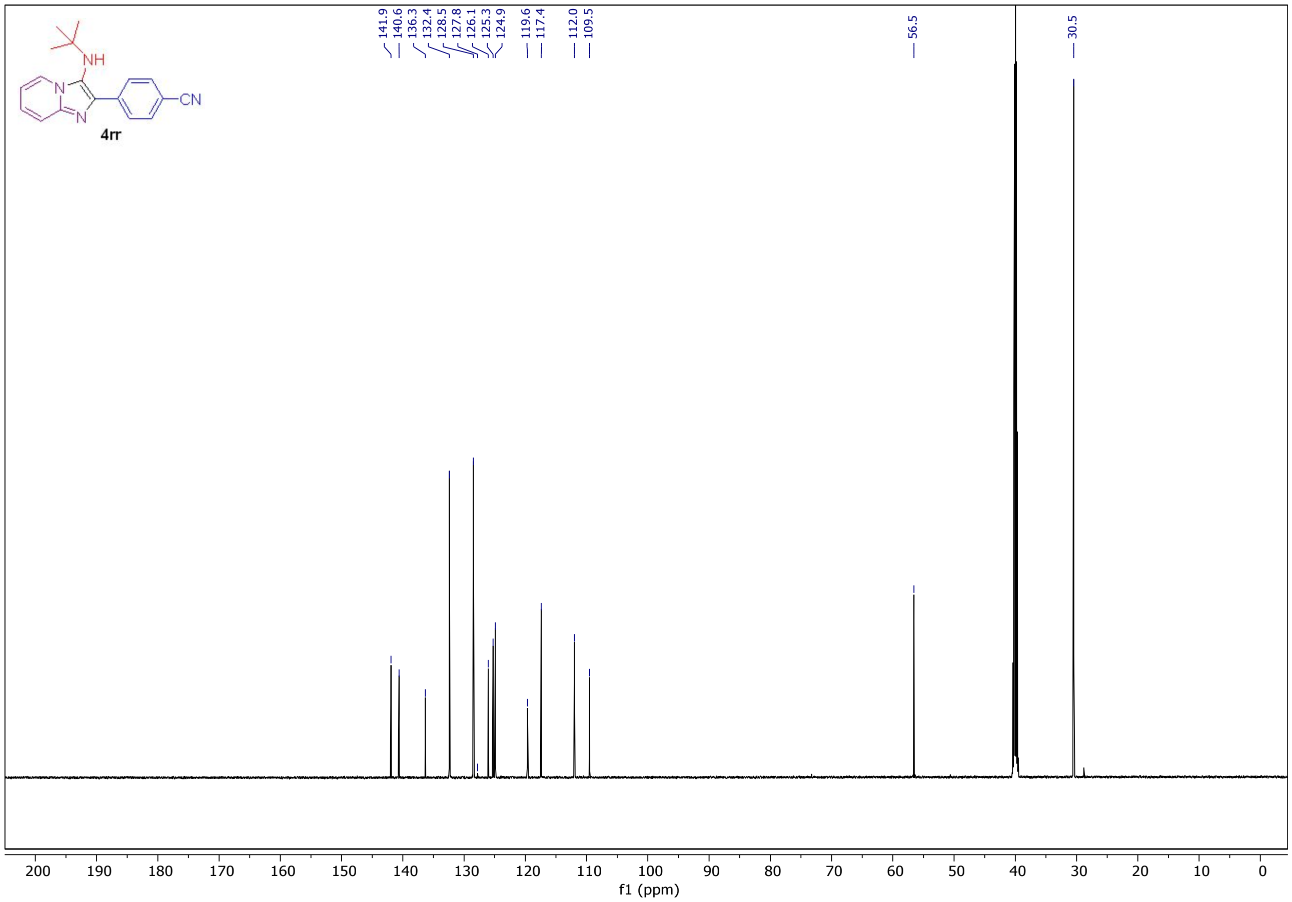

Figure S119. ^{13}C NMR spectrum (151 MHz, DMSO-d_6) of compound **4rr**.

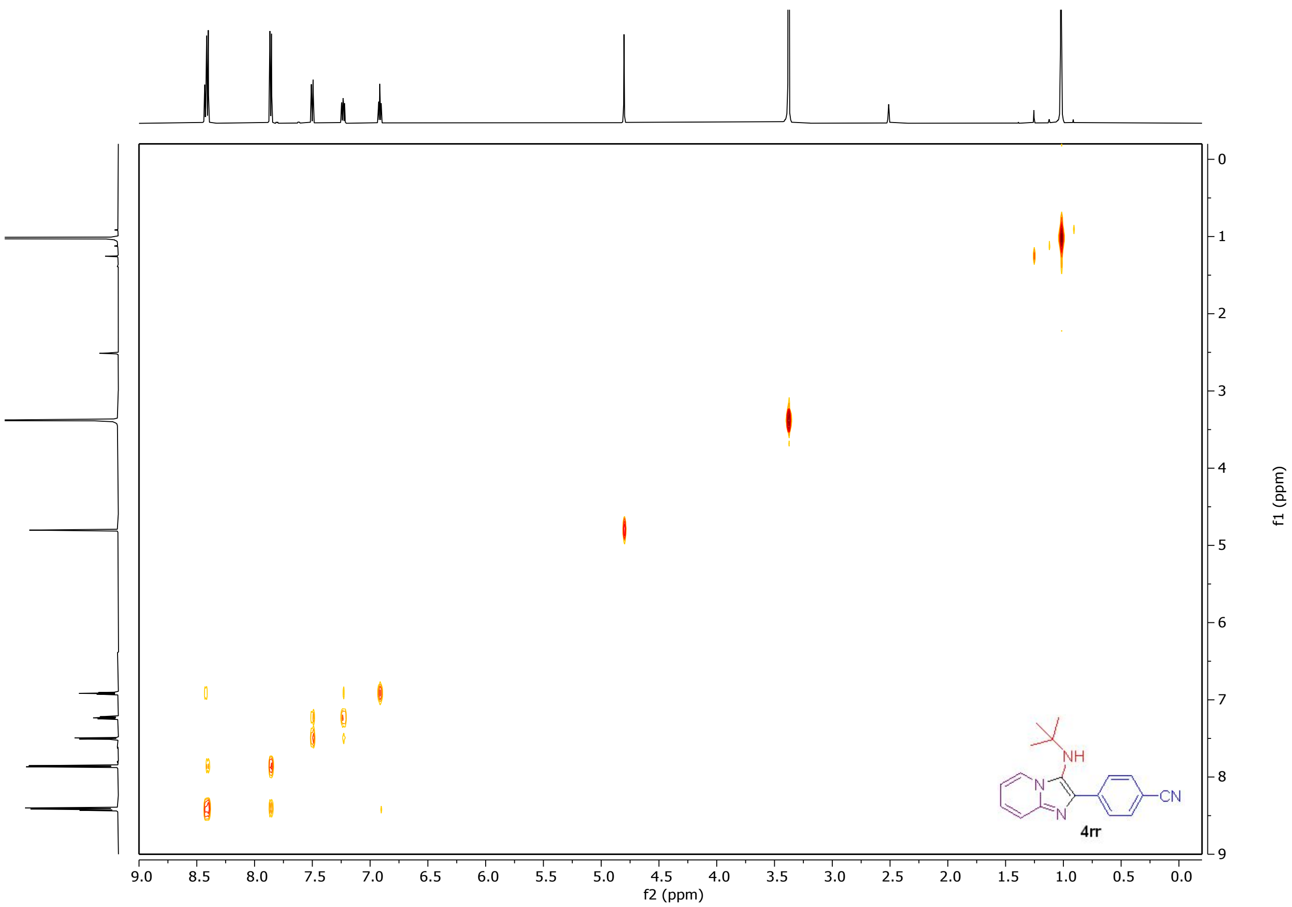

Figure S20. COSY 2D NMR spectrum (600 MHz, DMSO-d_6) of compound **4rr**.

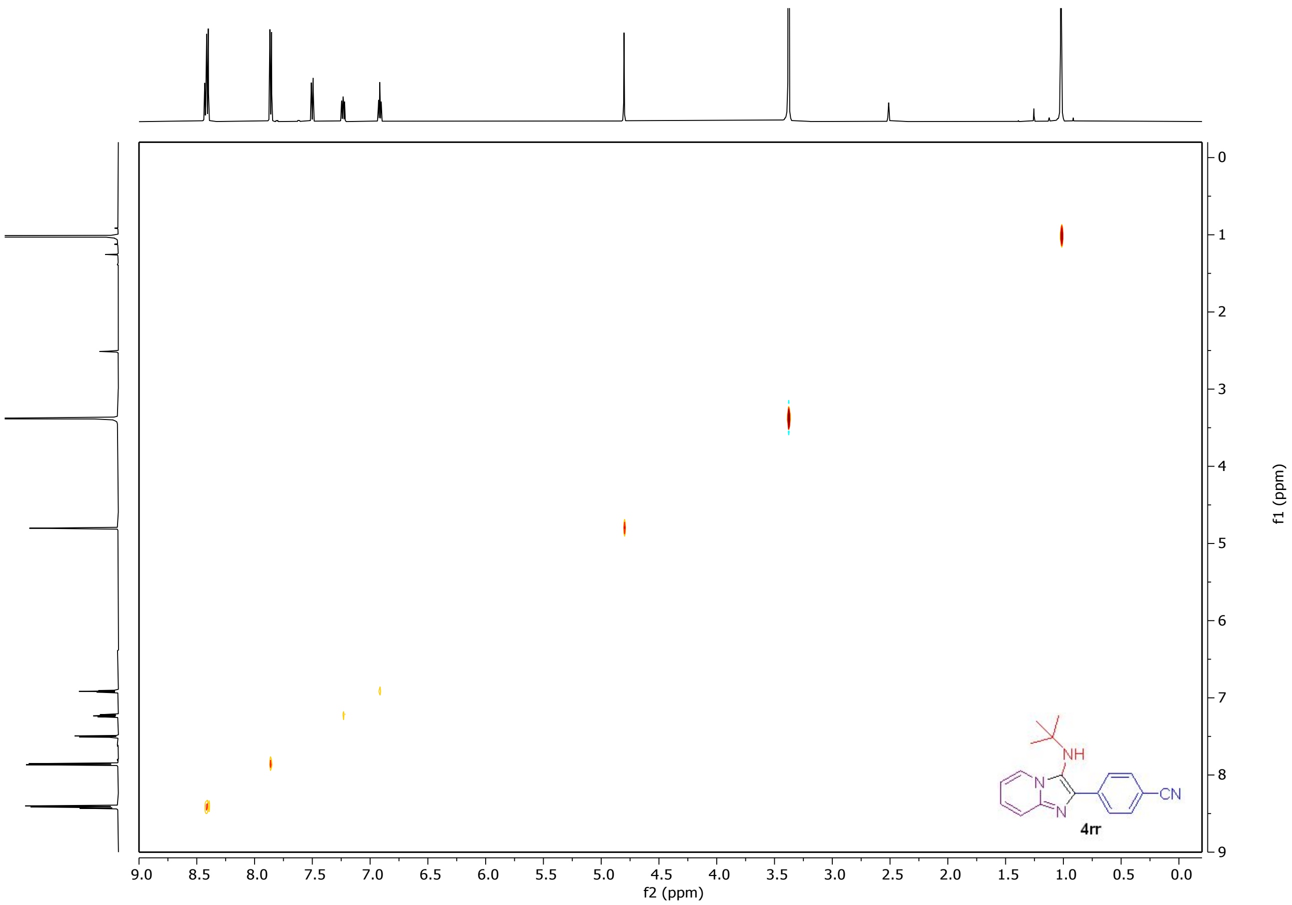

Figure S21. NOESY 2D NMR spectrum (600 MHz, DMSO-d_6) of compound **4rr**.

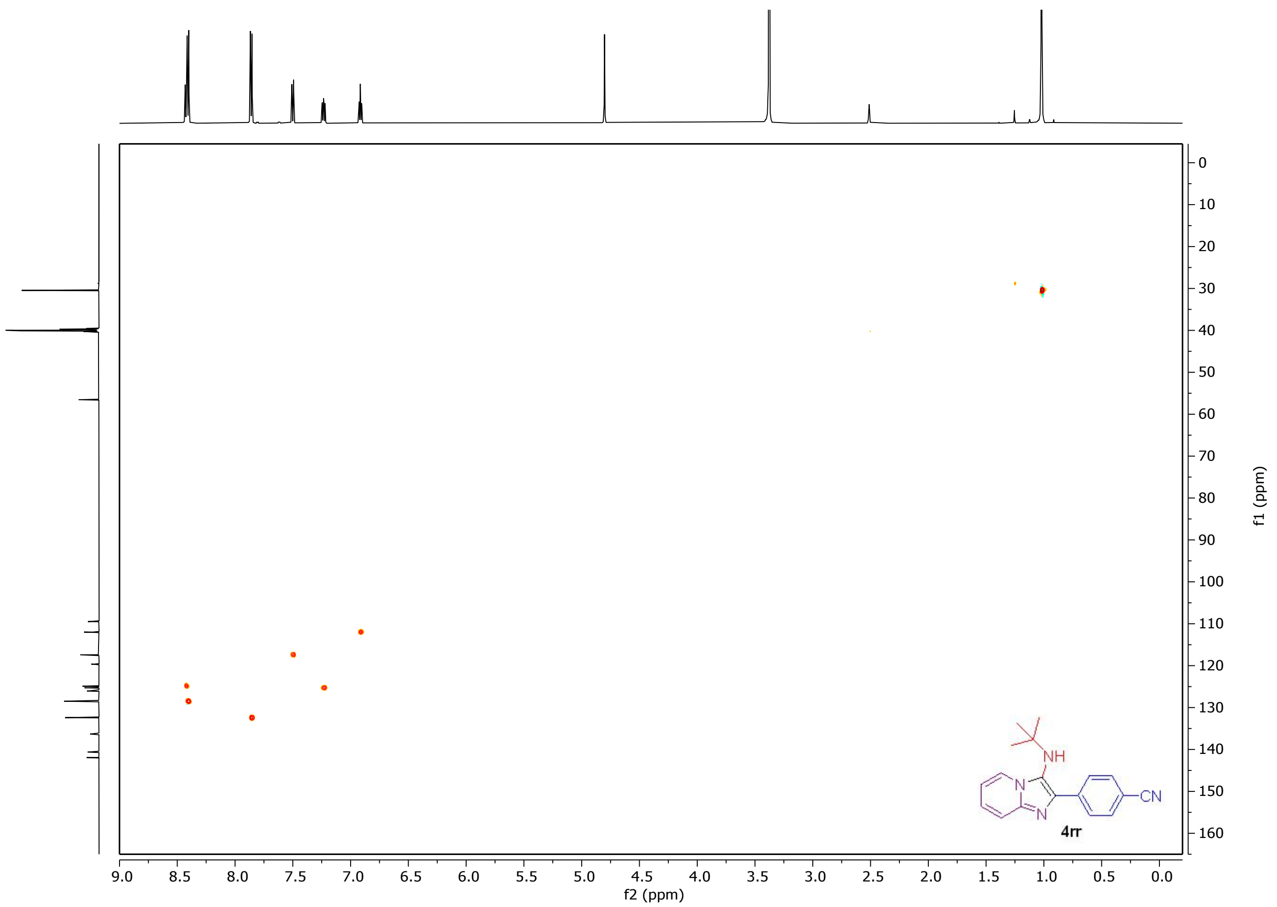

Figure S22. HSQC 2D NMR spectrum (600 MHz, DMSO-d_6) of compound **4rr**.

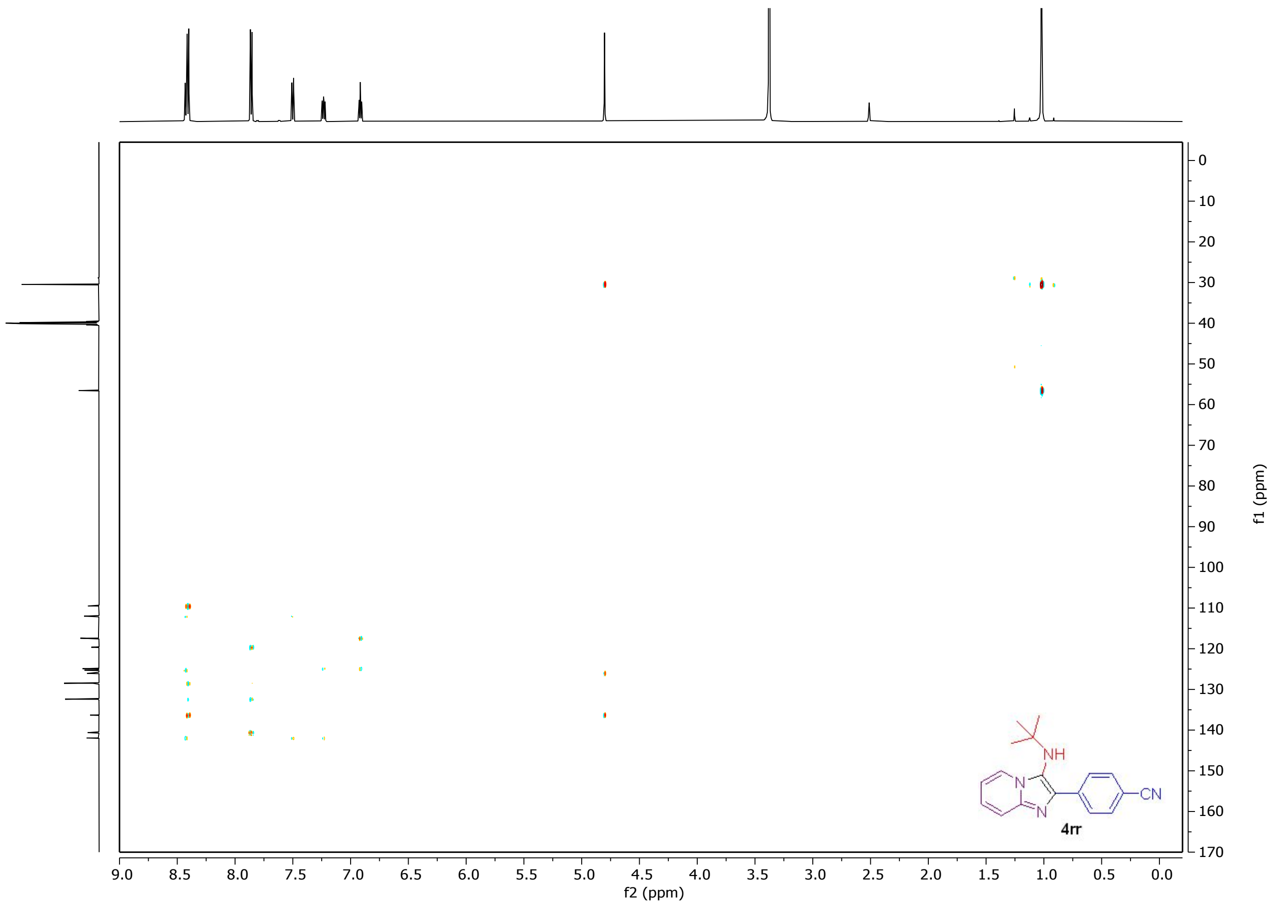

Figure S23. HMBC 2D NMR spectrum (600 MHz, DMSO-d_6) of compound **4rr**.

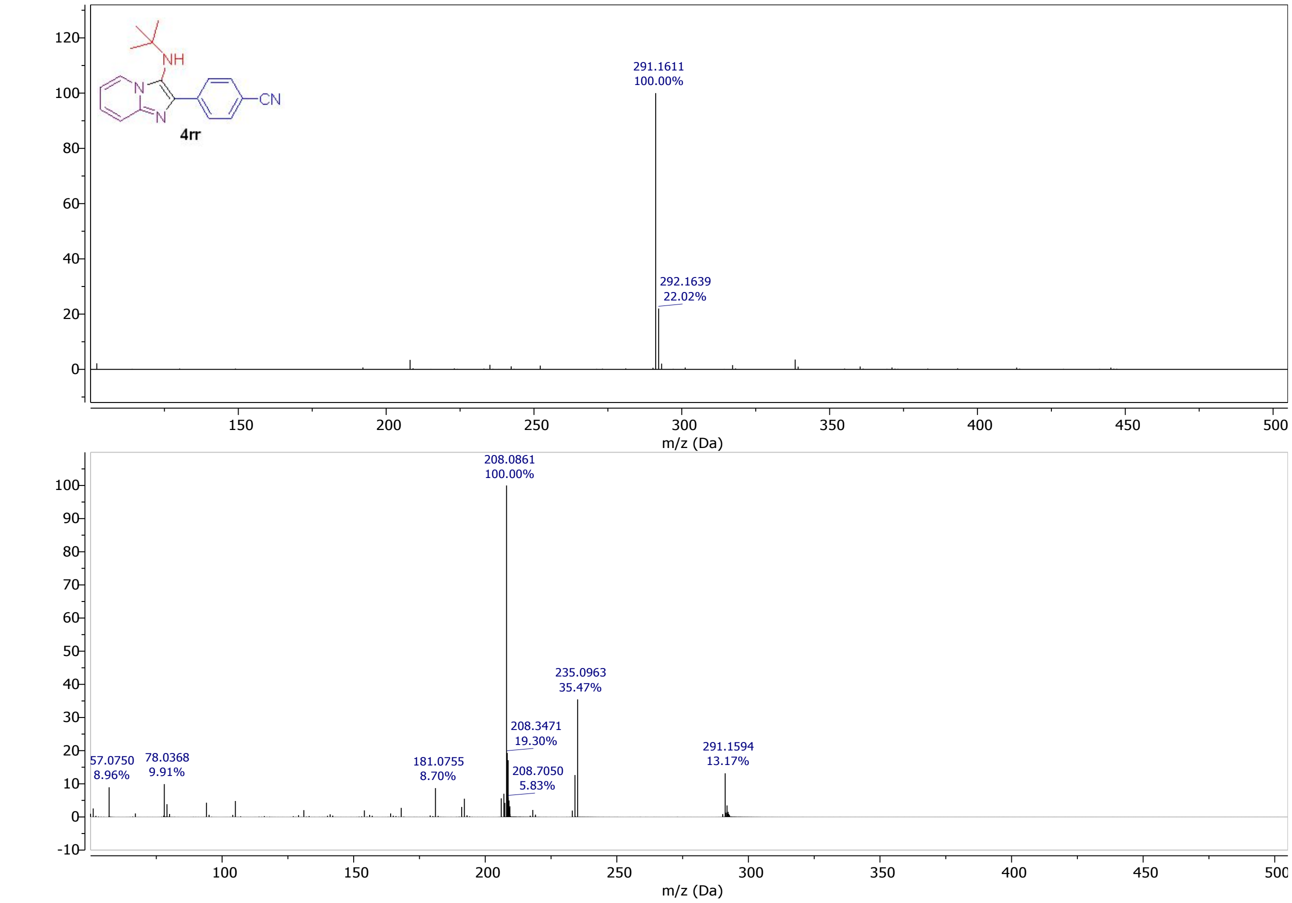

Figure S24. HRMS (ESI-QTOF) of compound **4rr** and HRMS/MS for $[M+H]^+$.

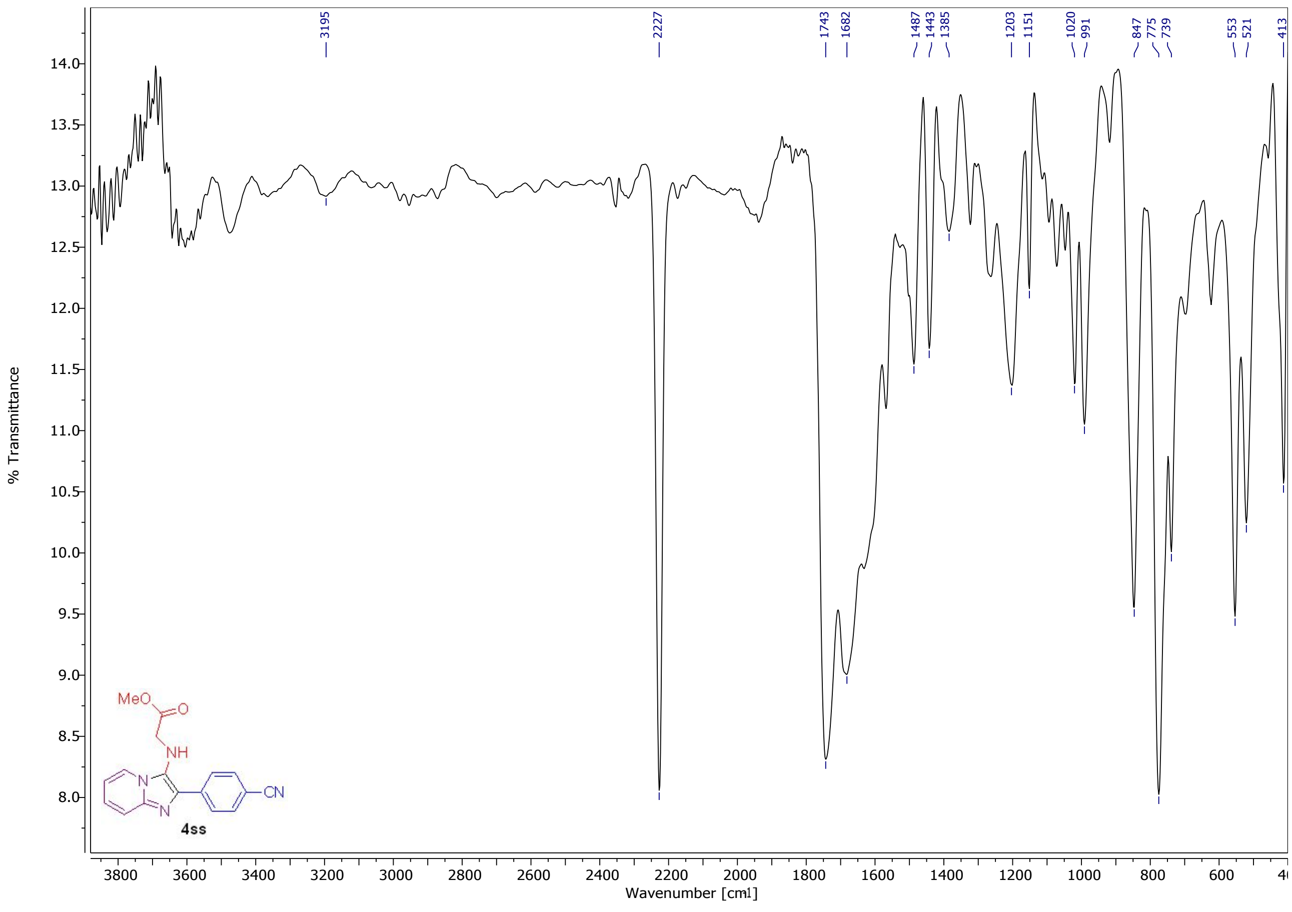

Figure S25. FT-IR (KBr) of compound **4ss**.

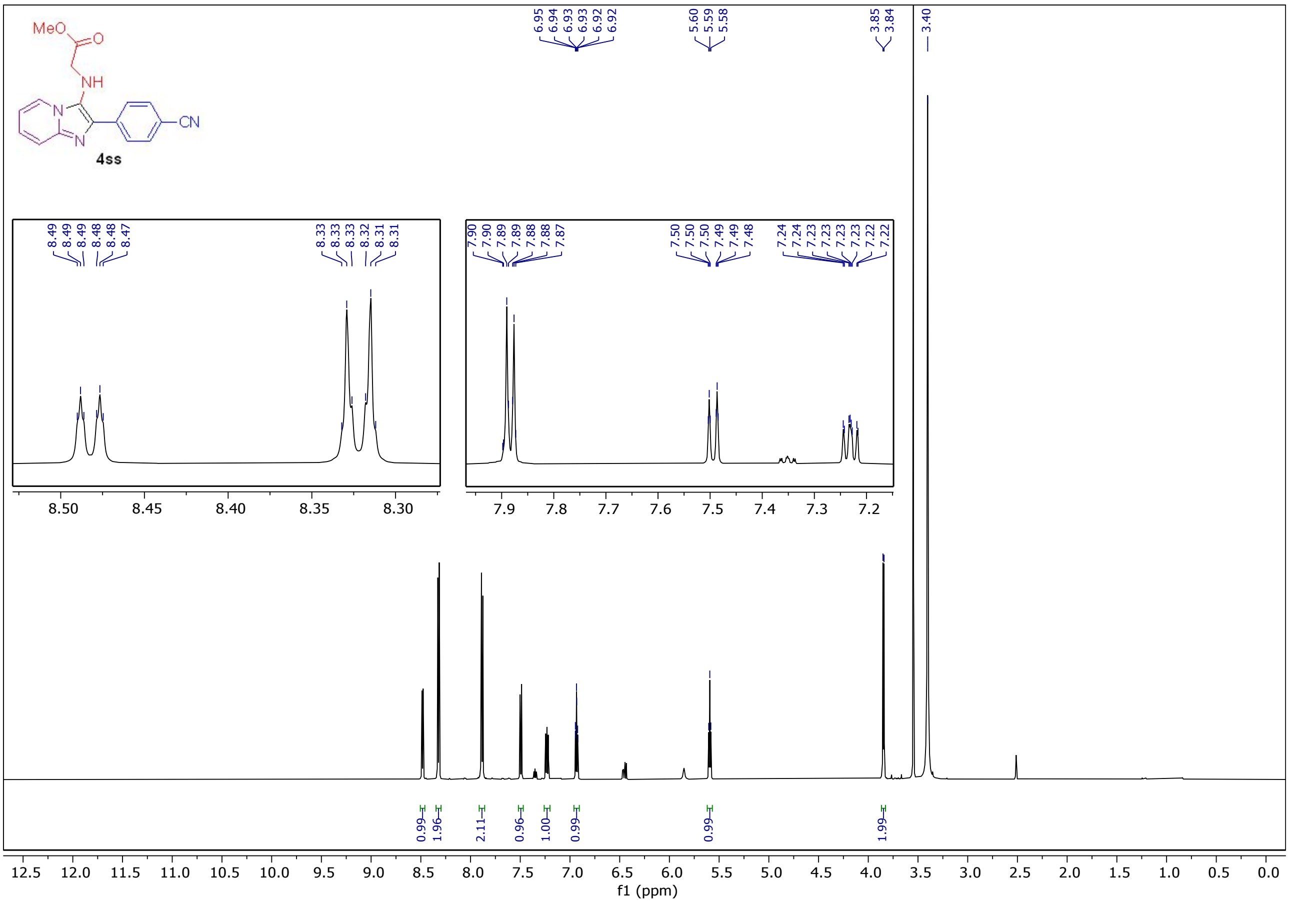

Figure S26. ^{1}H NMR spectrum (600 MHz, DMSO-d_6) of compound **4ss**.

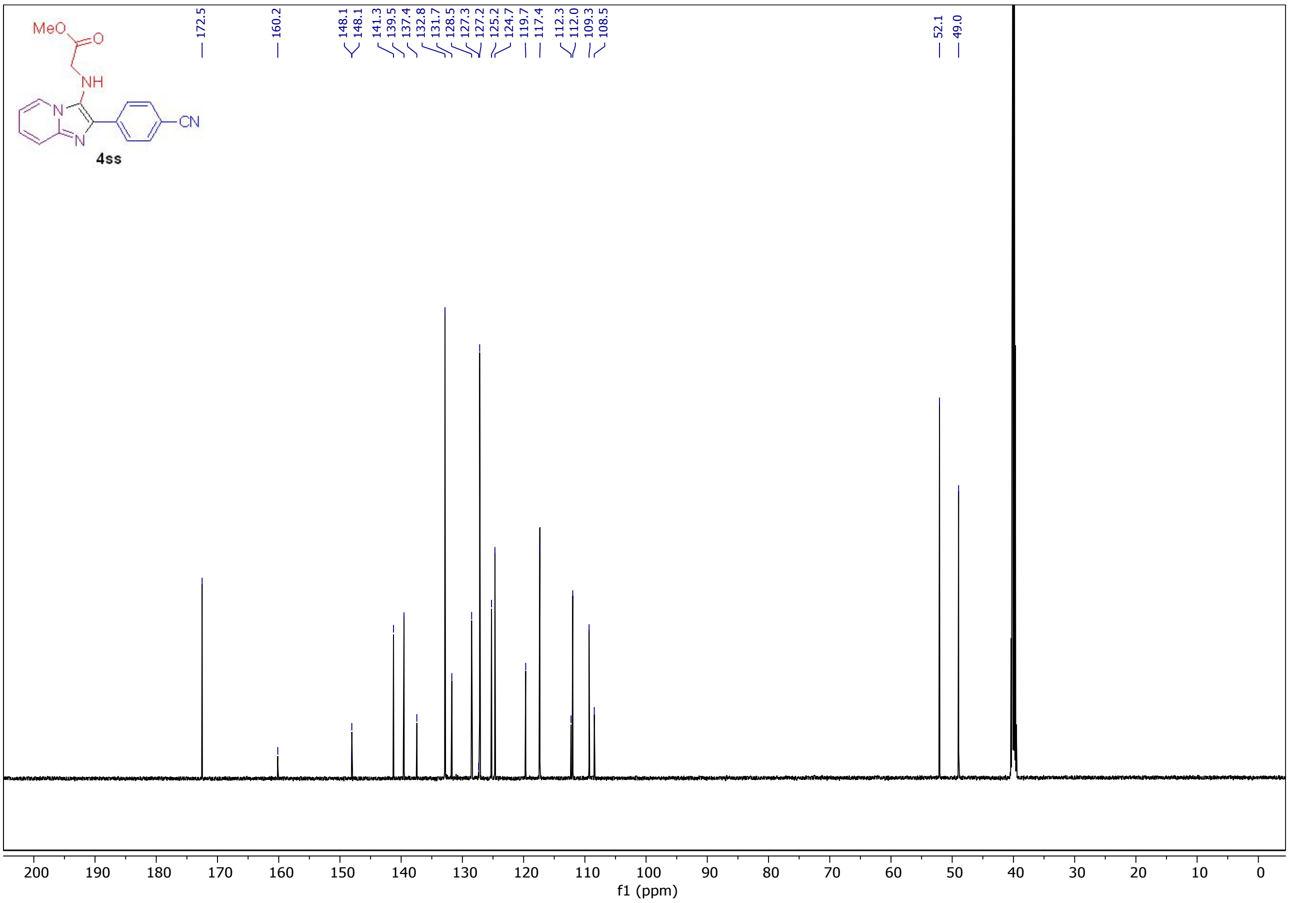

Figure S27. ^{13}C NMR spectrum (151 MHz, DMSO-d_6) of compound **4ss**.

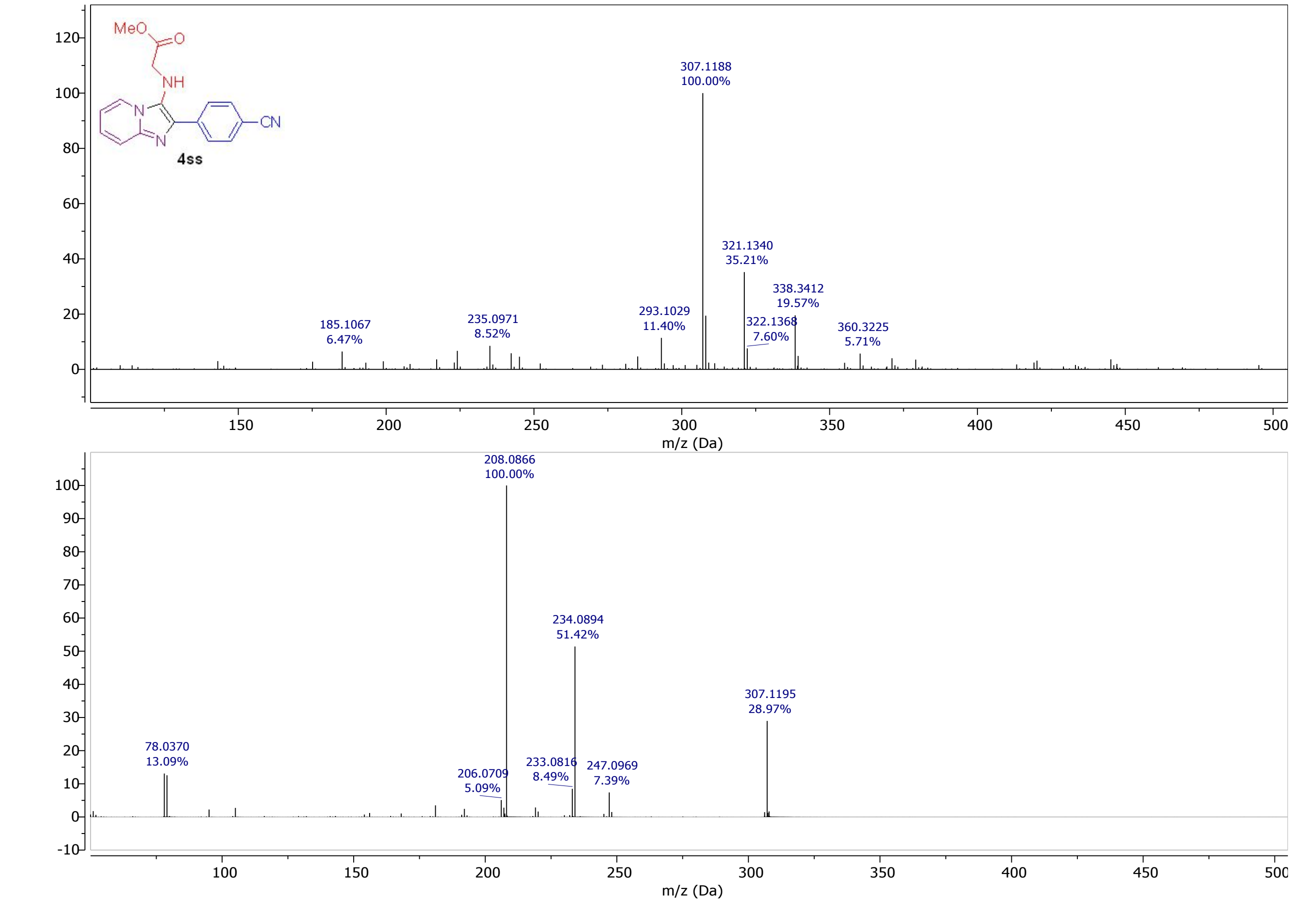

Figure S28. HRMS (ESI-QTOF) of compound **4ss** and HRMS/MS for $[M+H]^+$.

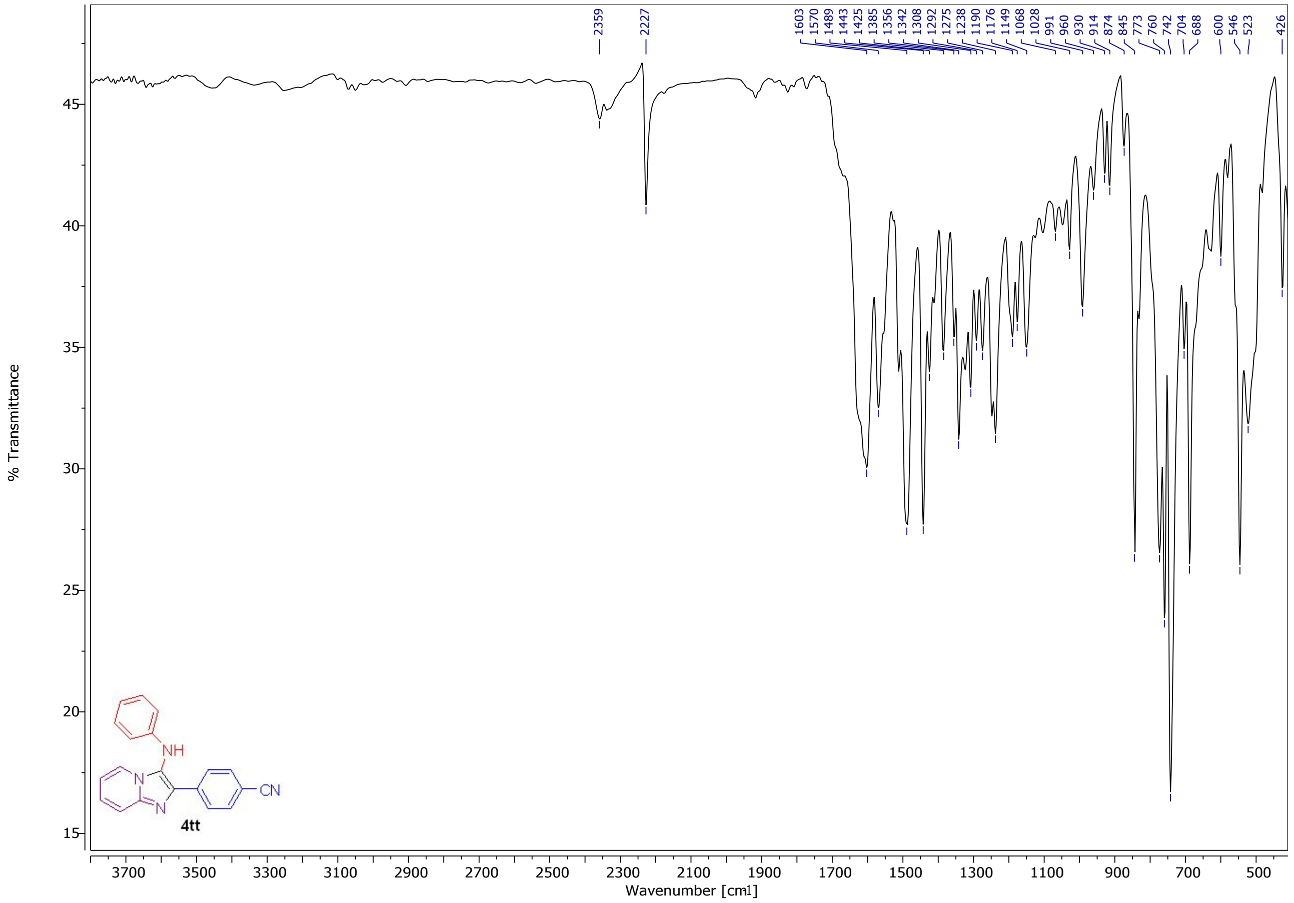

Figure S29. FT-IR (KBr) of compound **4tt**.

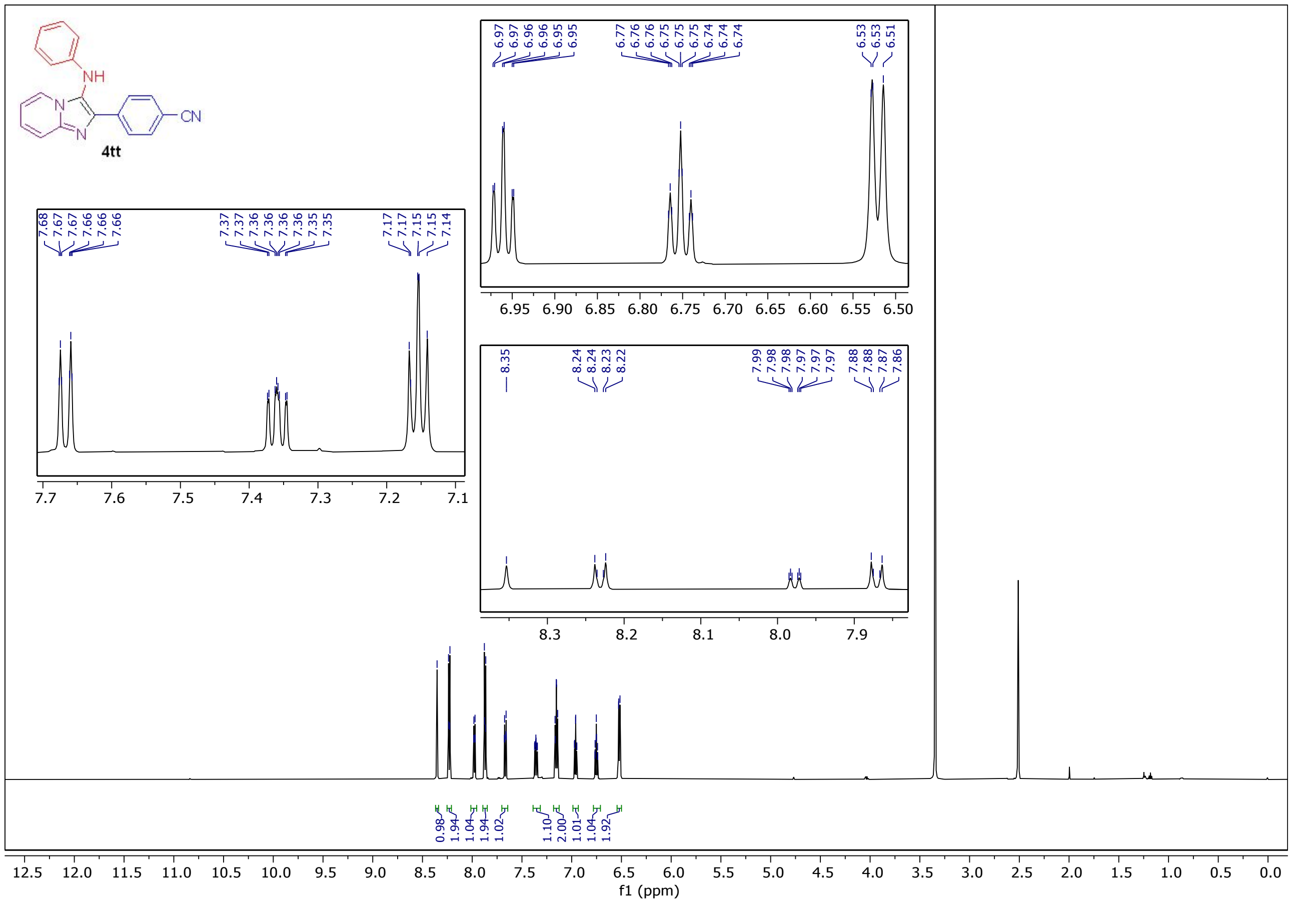

Figure S30. ^{1}H NMR spectrum (600 MHz, DMSO-d_6) of compound **4tt**.

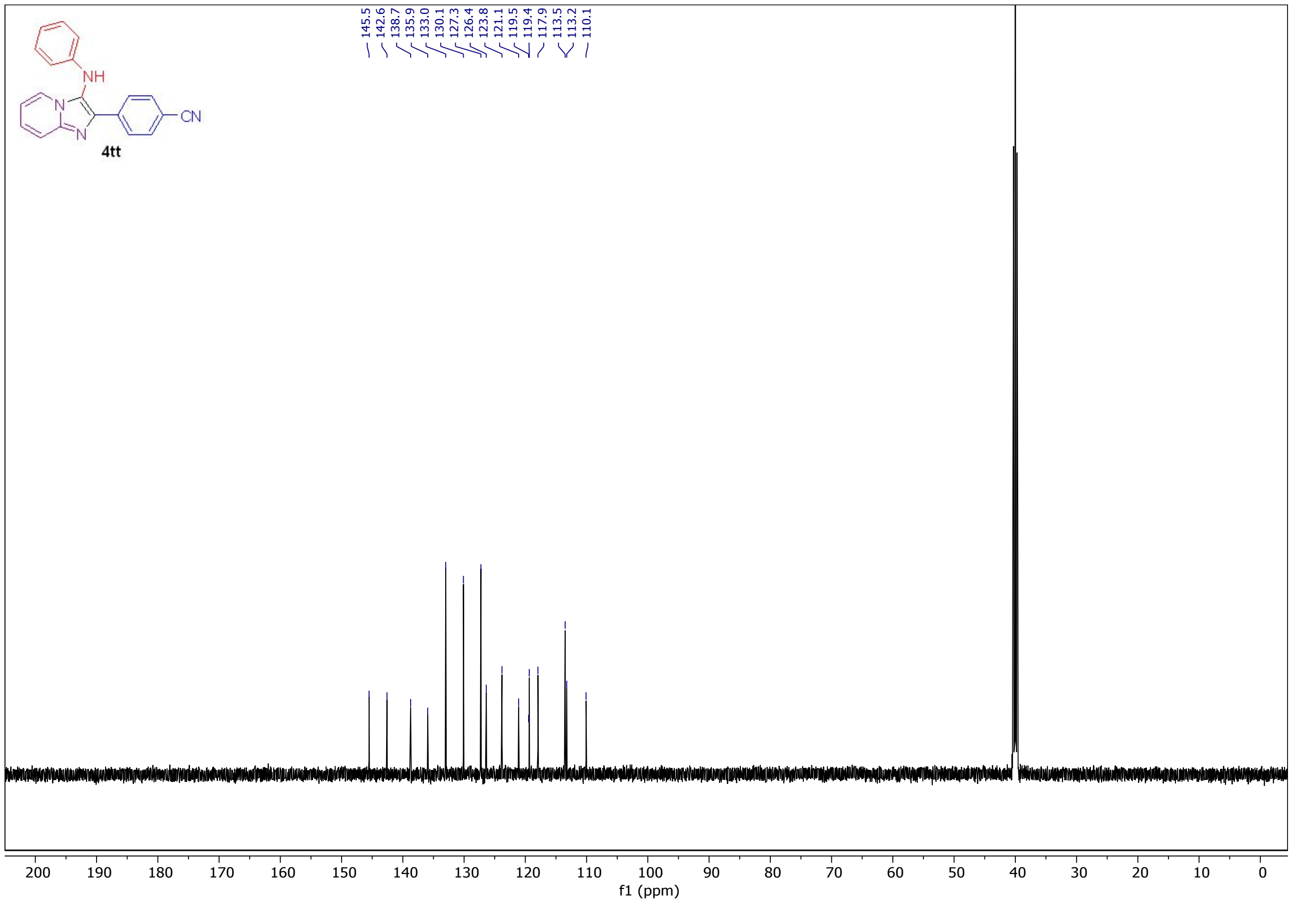

Figure S31. ^{13}C NMR spectrum (151 MHz, DMSO-d_6) of compound **4tt**.

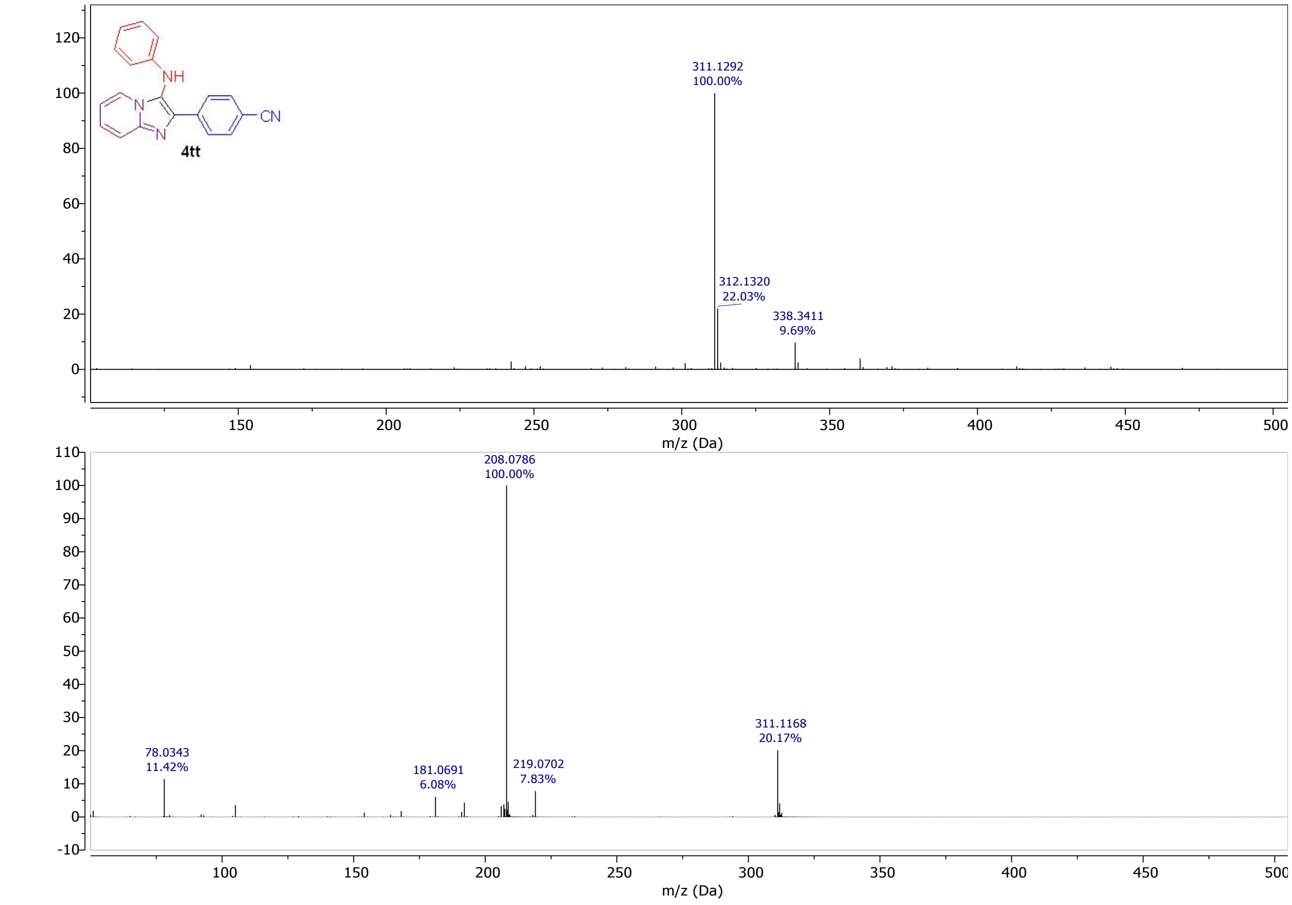

Figure S32. HRMS (ESI-QTOF) of compound **4tt** and HRMS/MS for $[M+H]^+$.

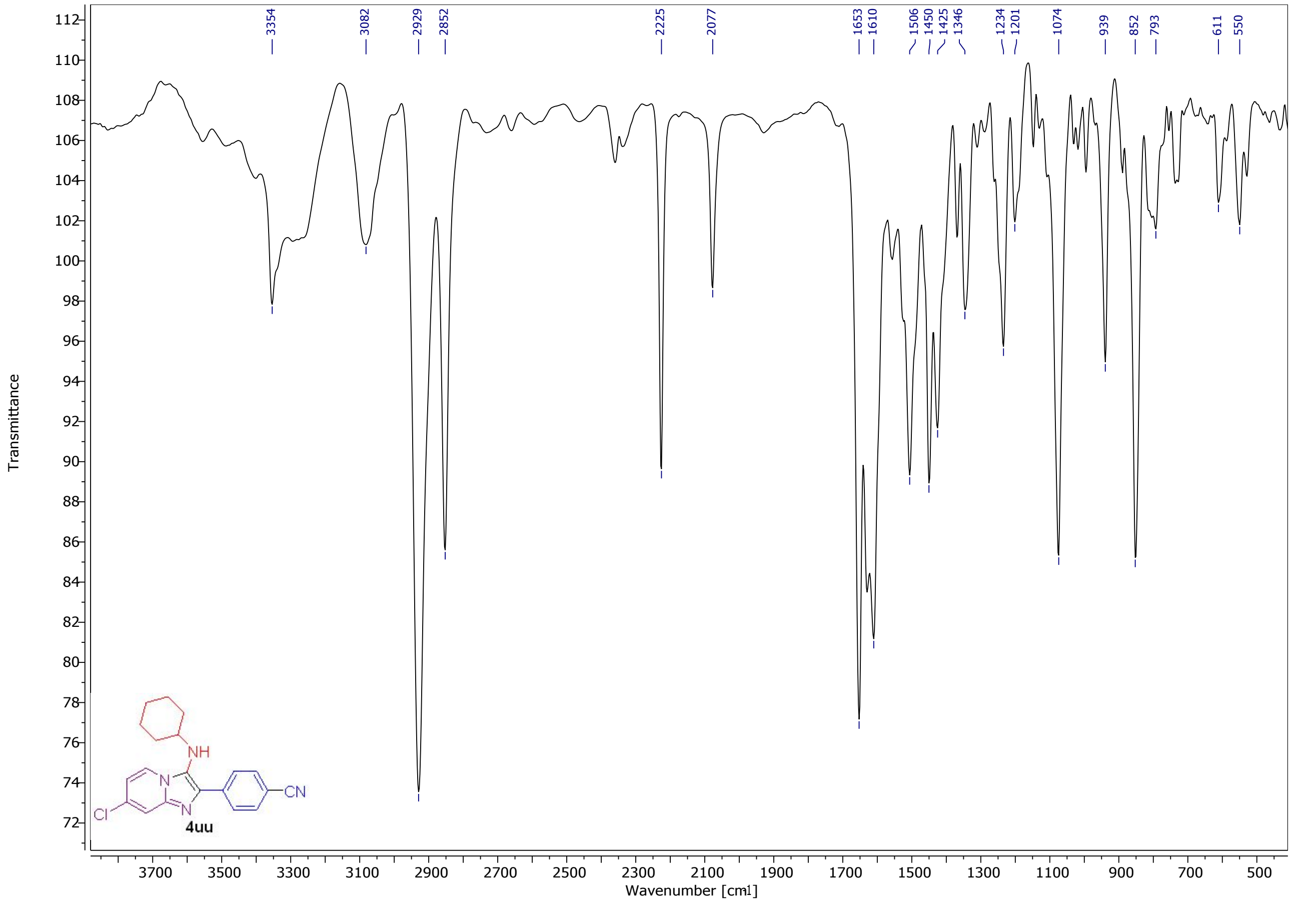

Figure S33. FT-IR (KBr) of compound **4uu**.

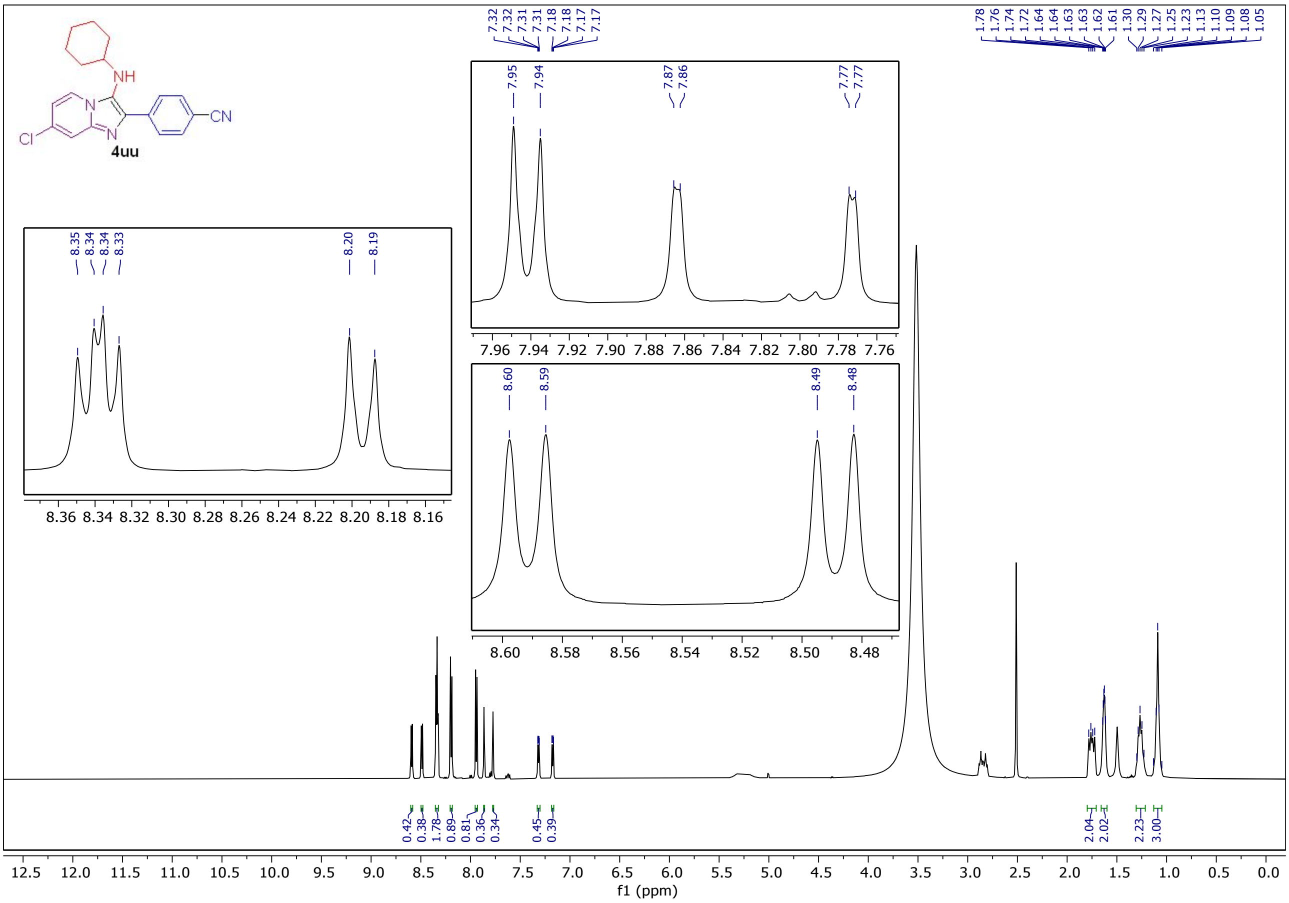

Figure S34. ^{1}H NMR spectrum (600 MHz, DMSO-d_6) of compound **4uu**.

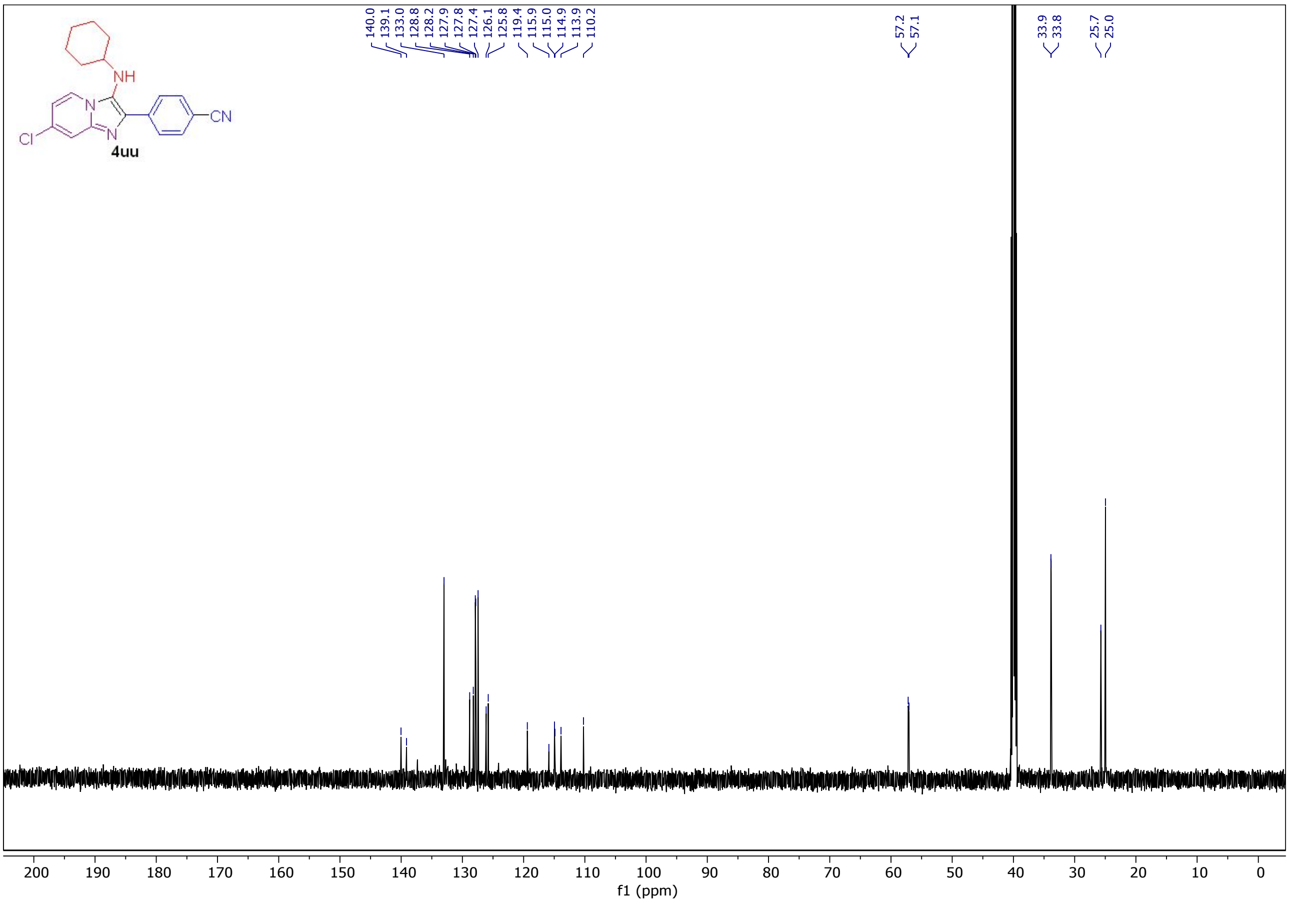

Figure S35. ^{13}C NMR spectrum (151 MHz, DMSO-d_6) of compound **4uu**.

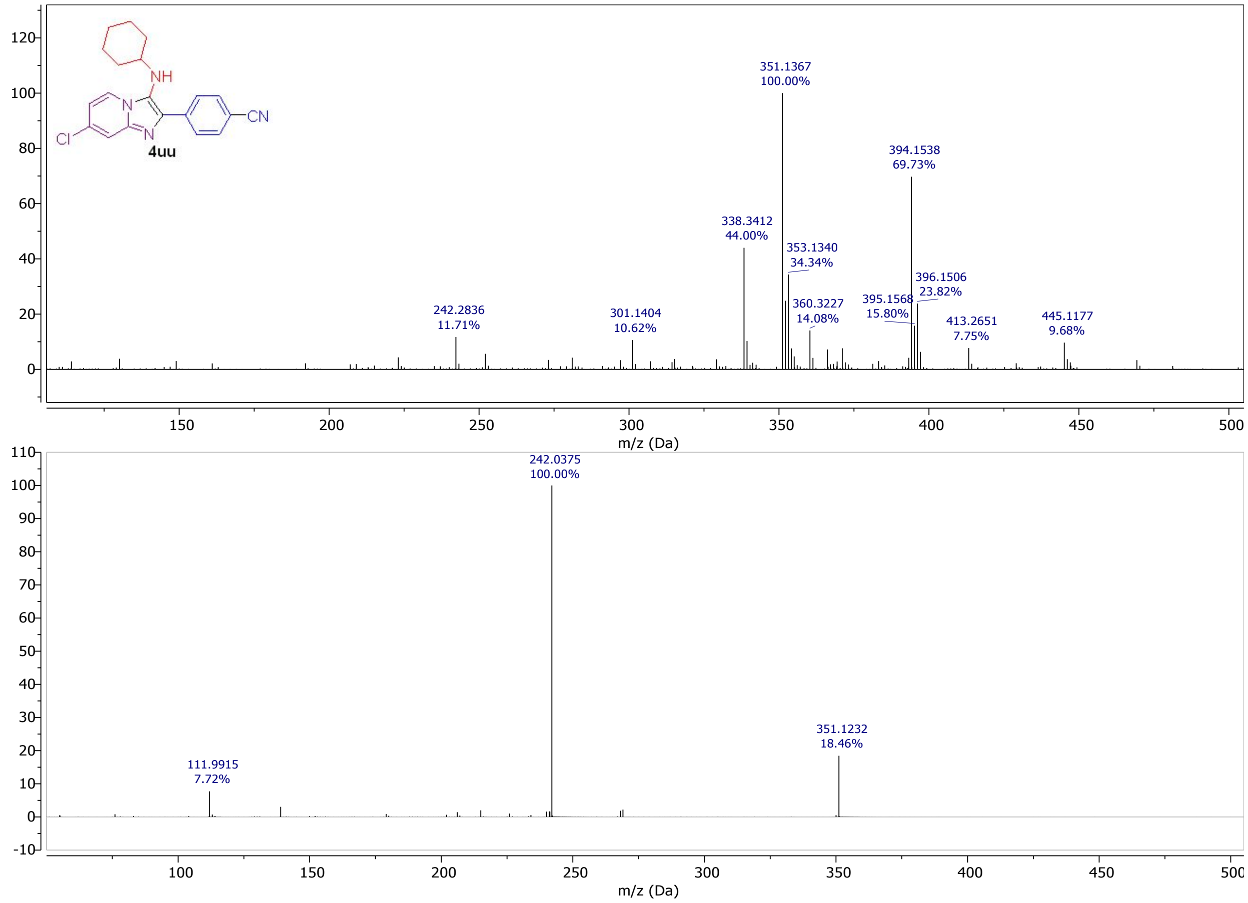

Figure S36. HRMS (ESI-QTOF) of compound **4uu** and HRMS/MS for $[M+H]^+$.

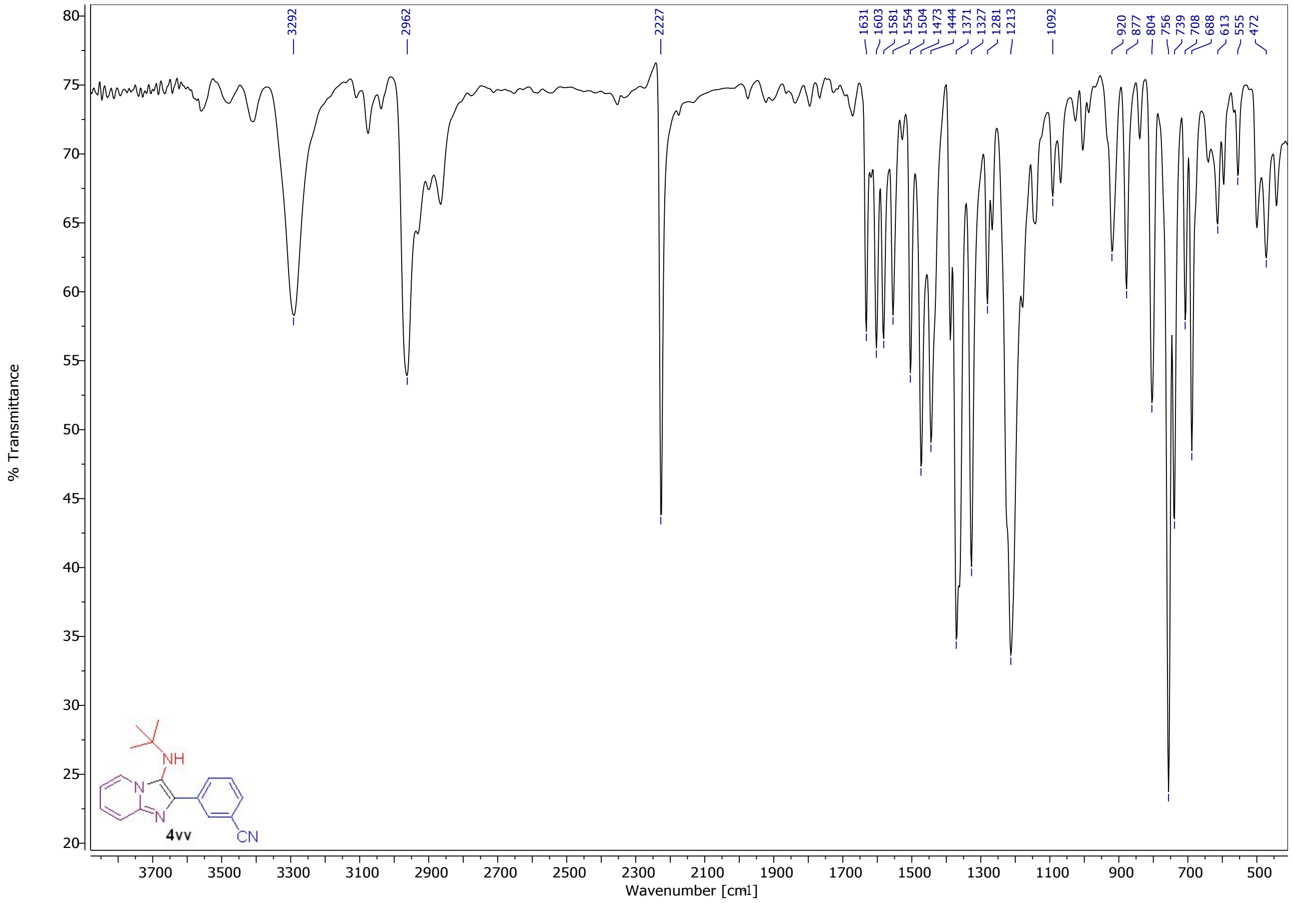

Figure S37. FT-IR (KBr) of compound **4vv**.

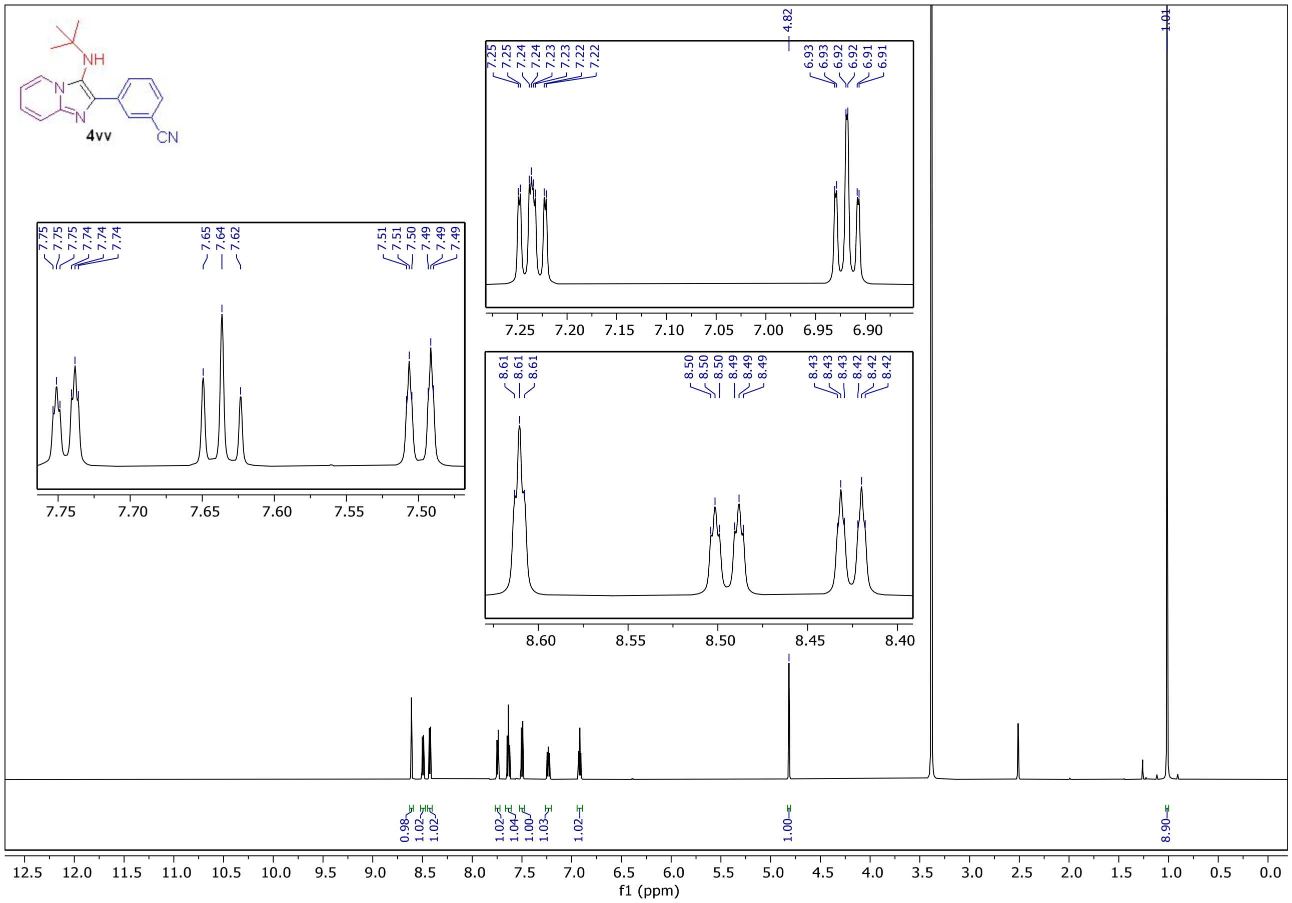

Figure S38. ^{1}H NMR spectrum (600 MHz, DMSO-d_6) of compound **4vv**.

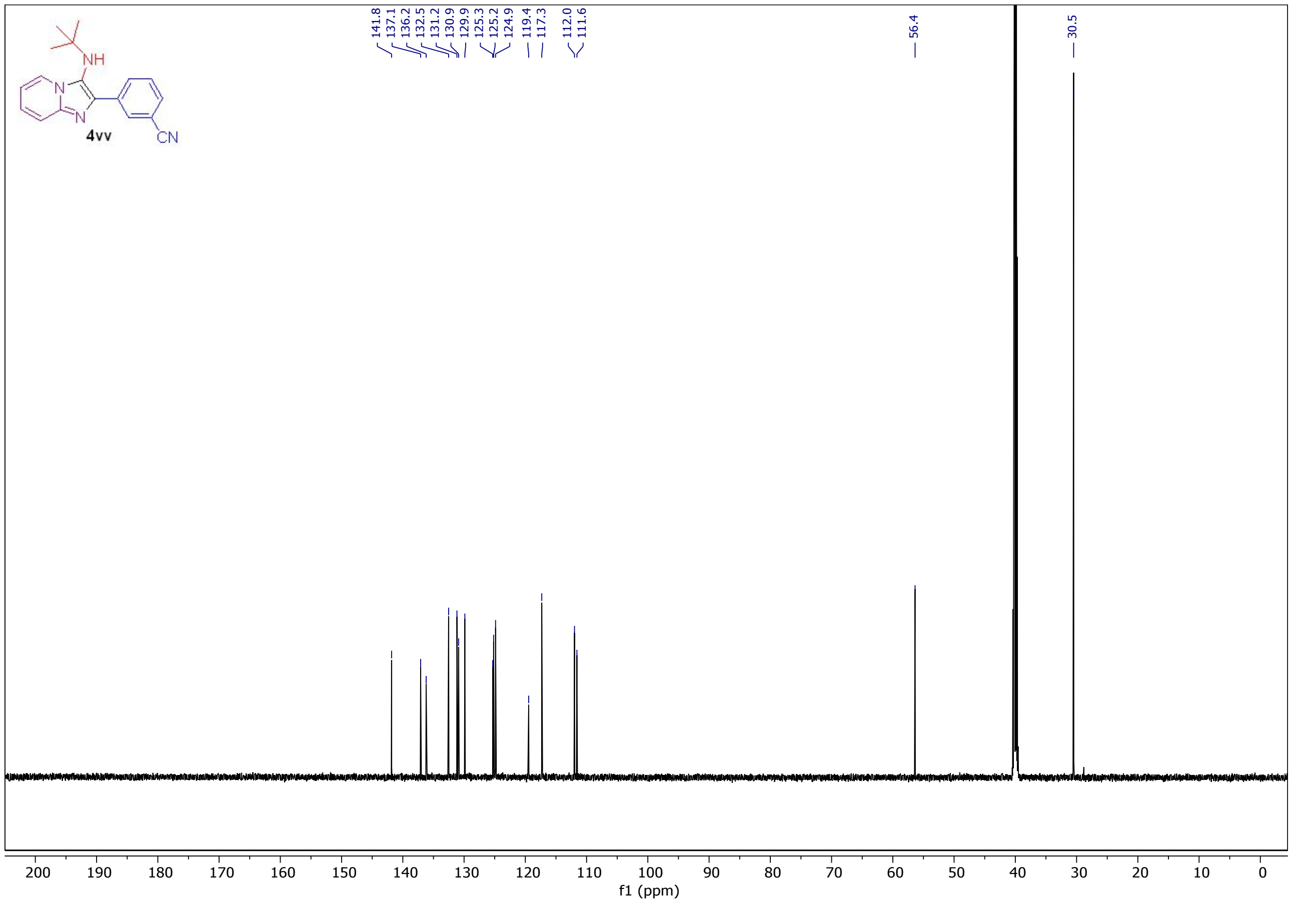

Figure S39. ^{13}C NMR spectrum (151 MHz, DMSO-d_6) of compound **4vv**.

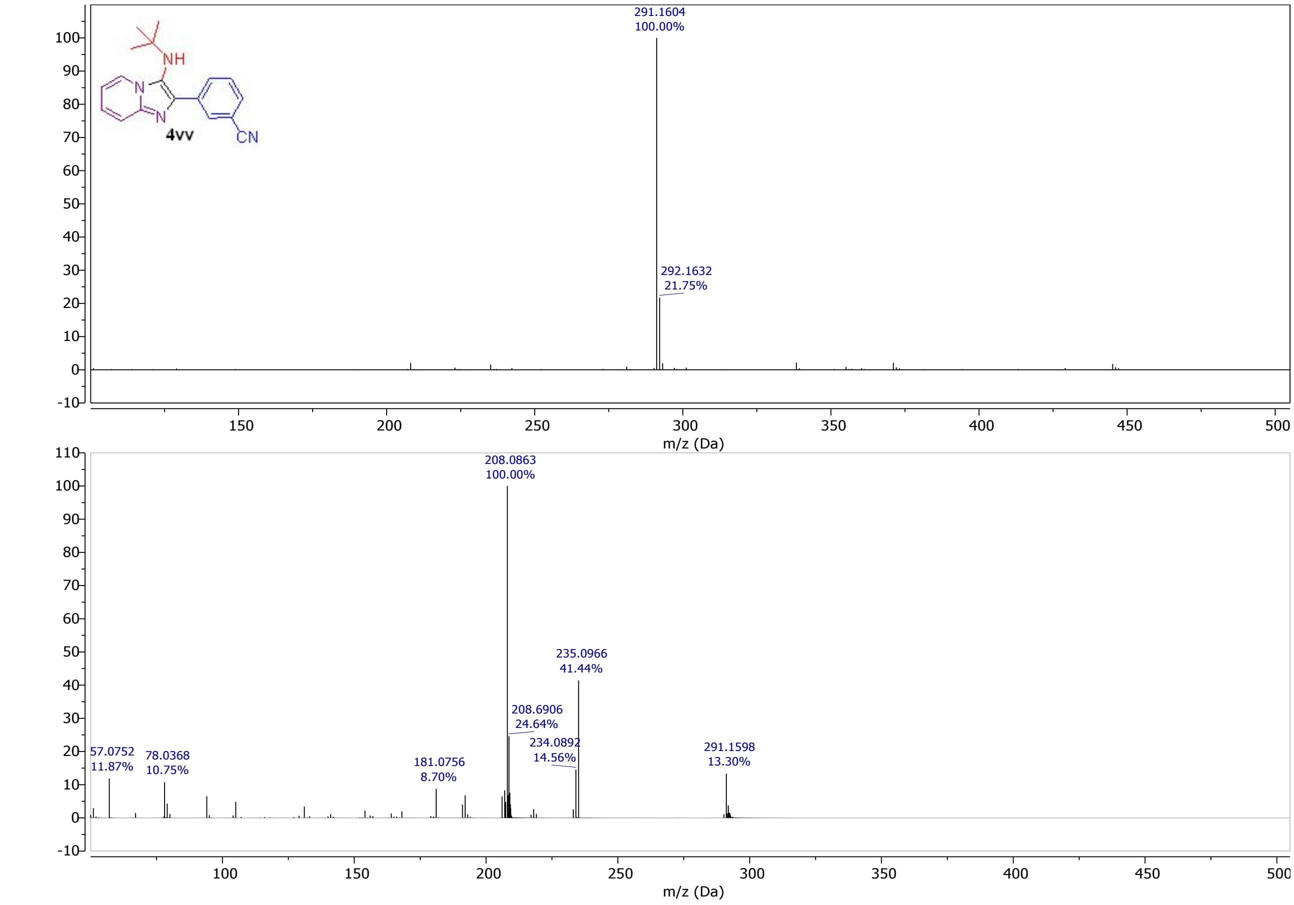

Figure S40. HRMS (ESI-QTOF) of compound **4vv** and HRMS/MS for [M+H]$^+$.

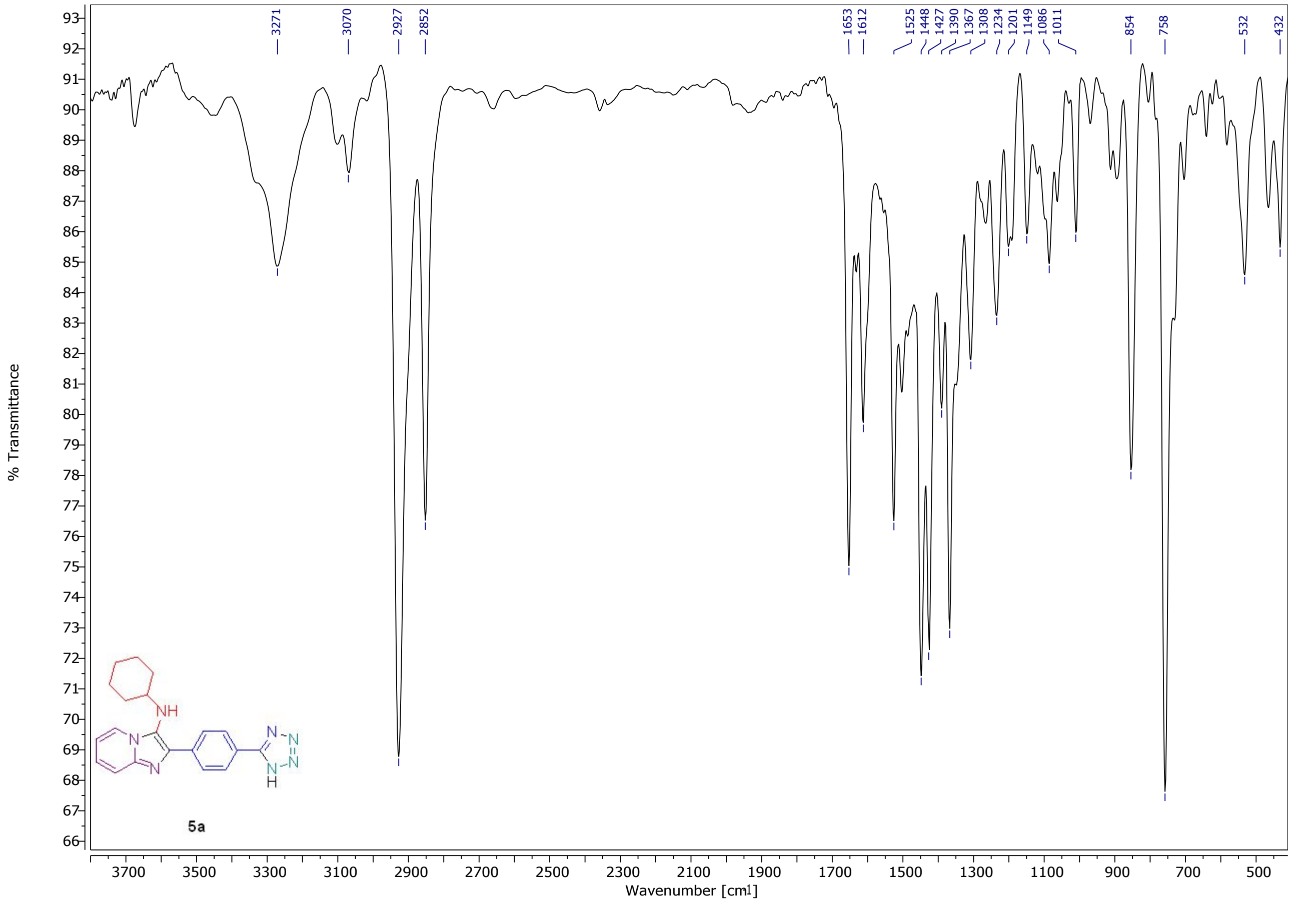

Figure S41. FT-IR (KBr) of compound **5a**.

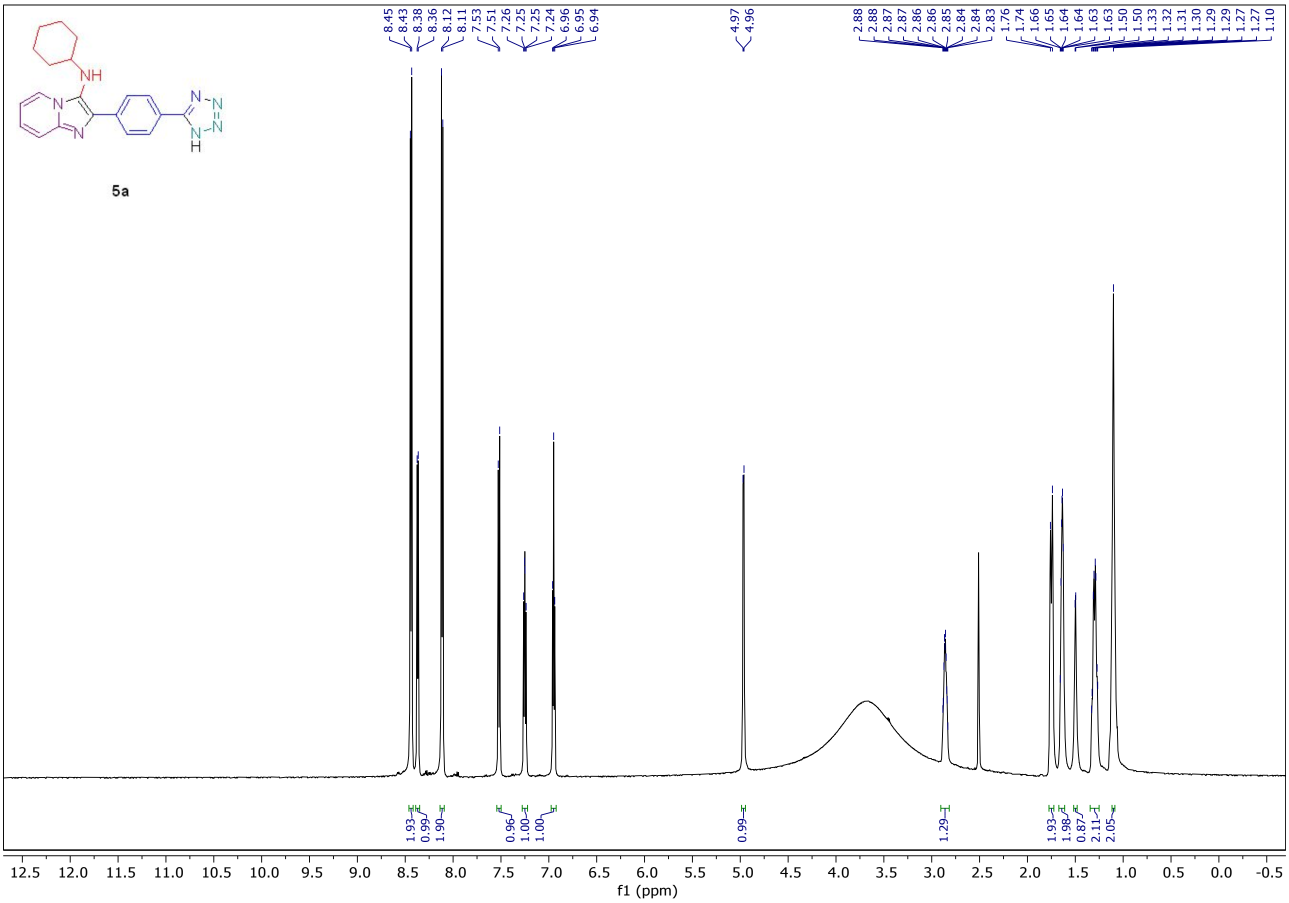

Figure S42. ^{1}H NMR spectrum (600 MHz, DMSO-d_6) of compound **5a**.

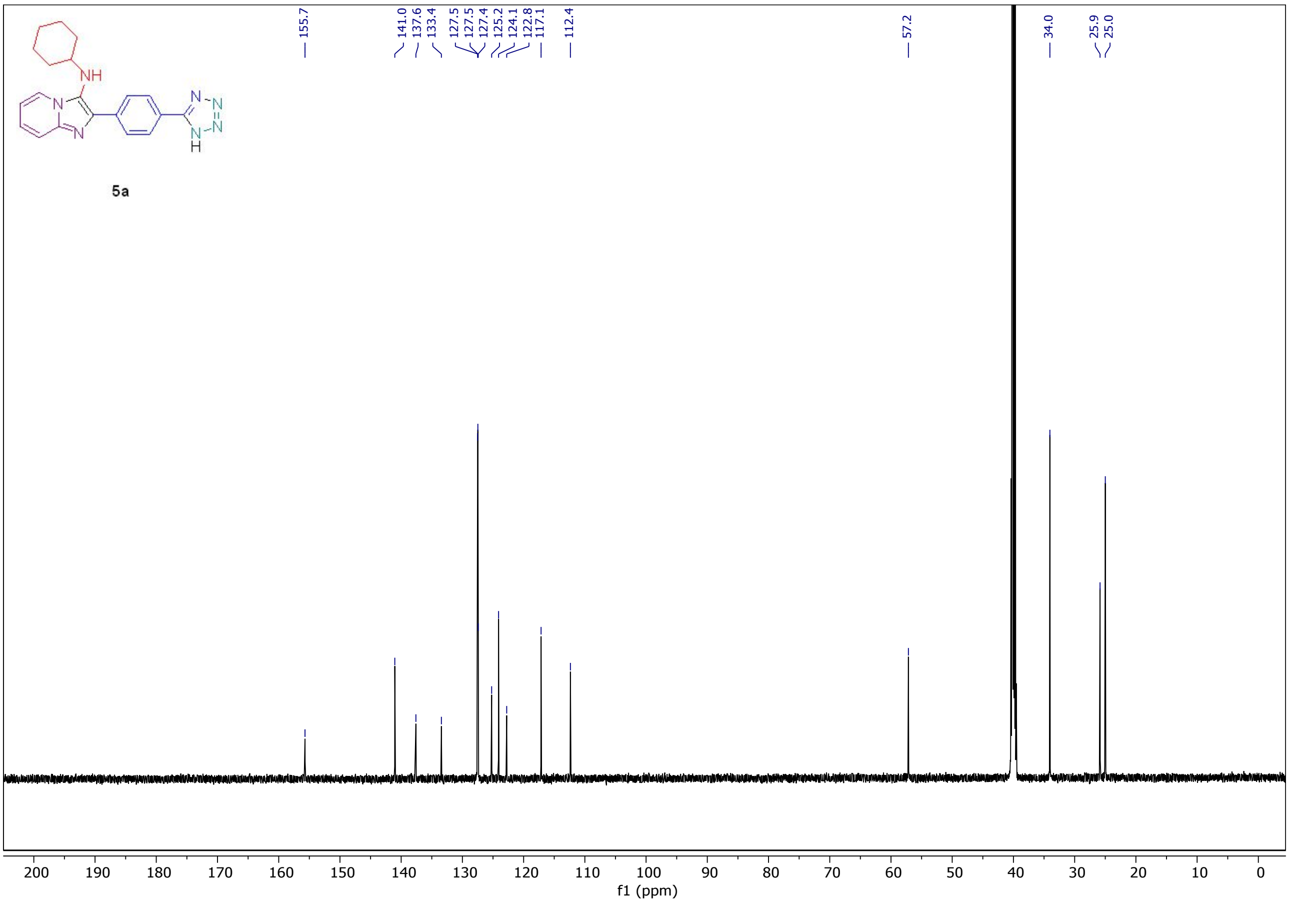

Figure S43. ^{13}C NMR spectrum (151 MHz, DMSO-d_6) of compound **5a**.

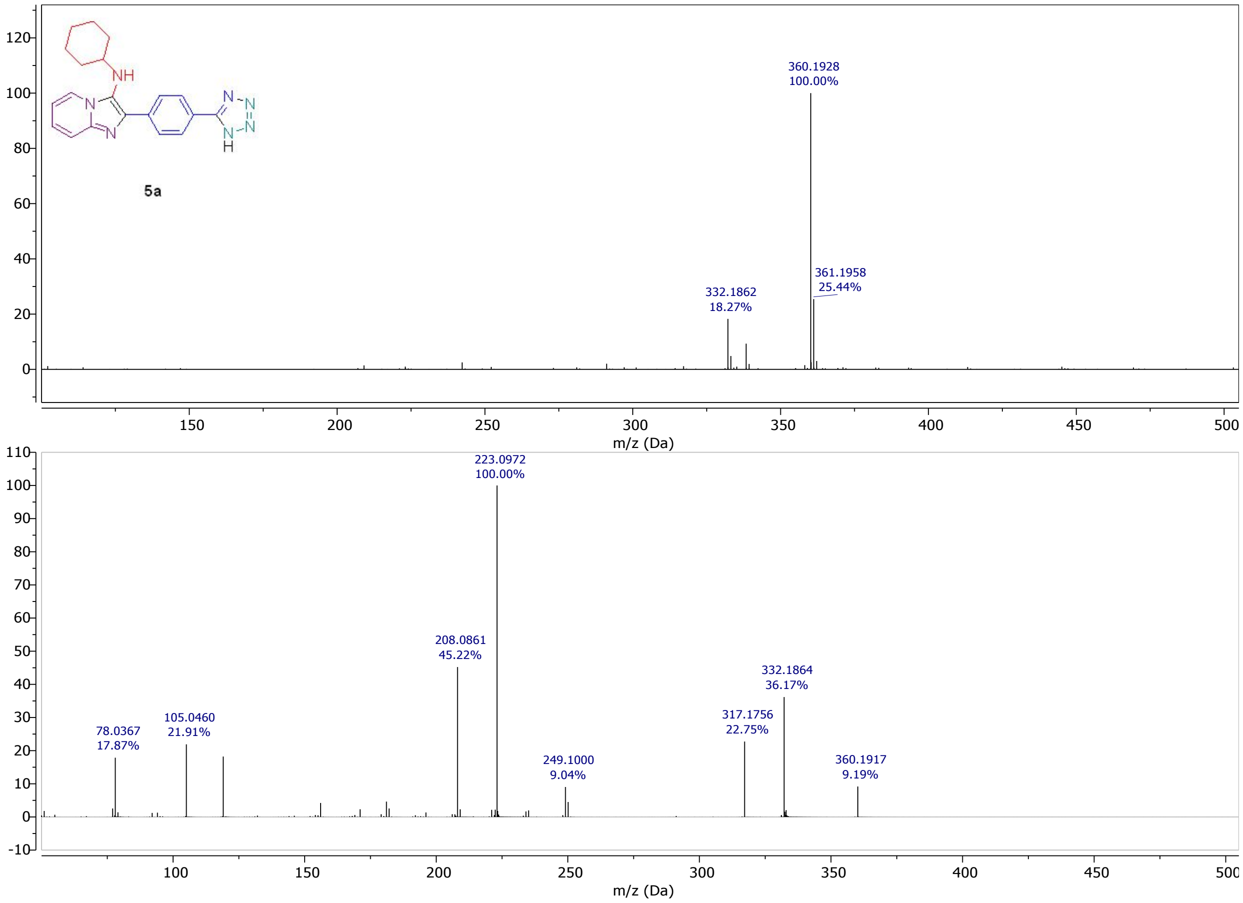

Figure S44. HRMS (ESI-QTOF) of compound **5a** and HRMS/MS for $[M+H]^+$.

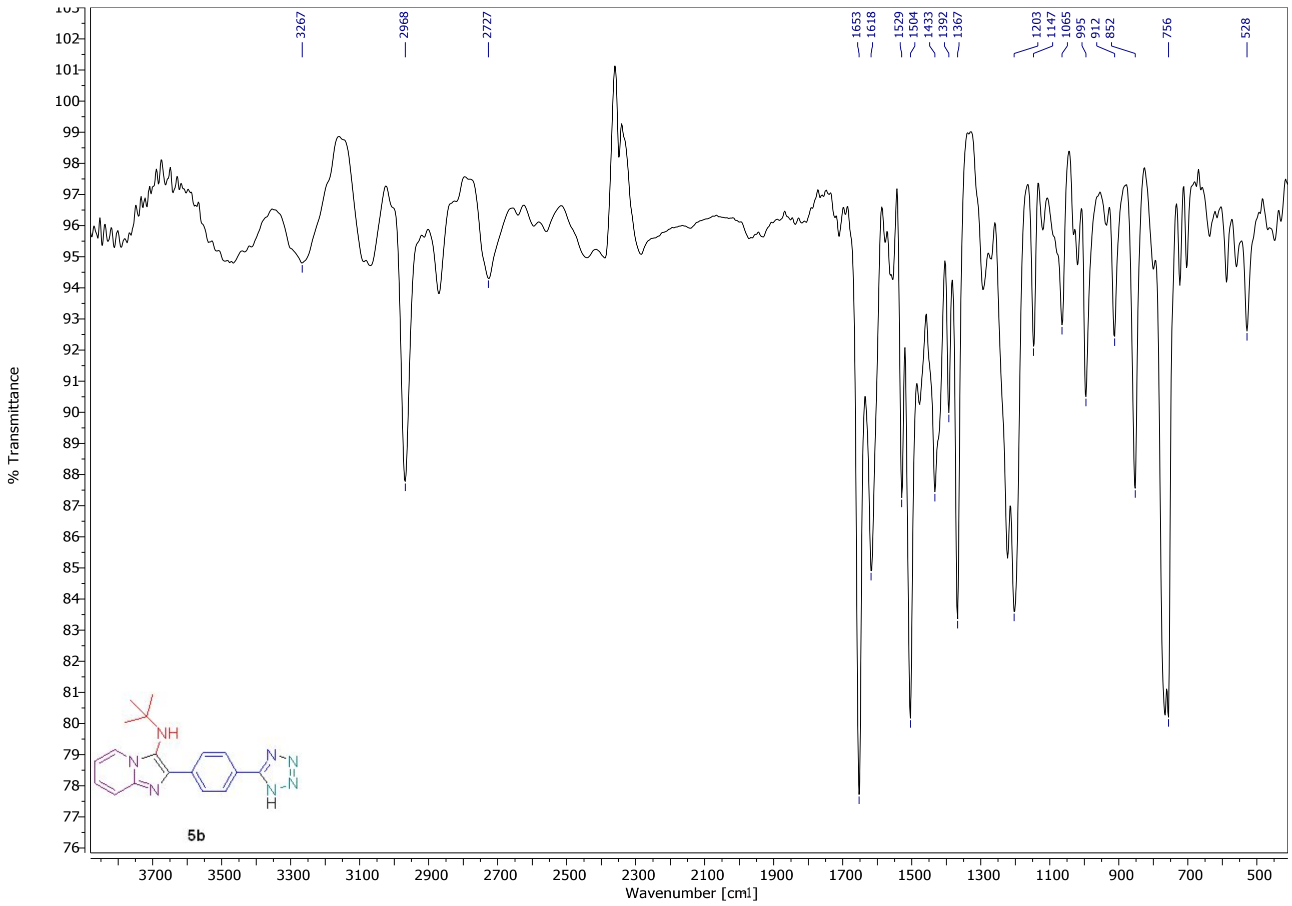

Figure S45. FT-IR (KBr) of compound **5b**.

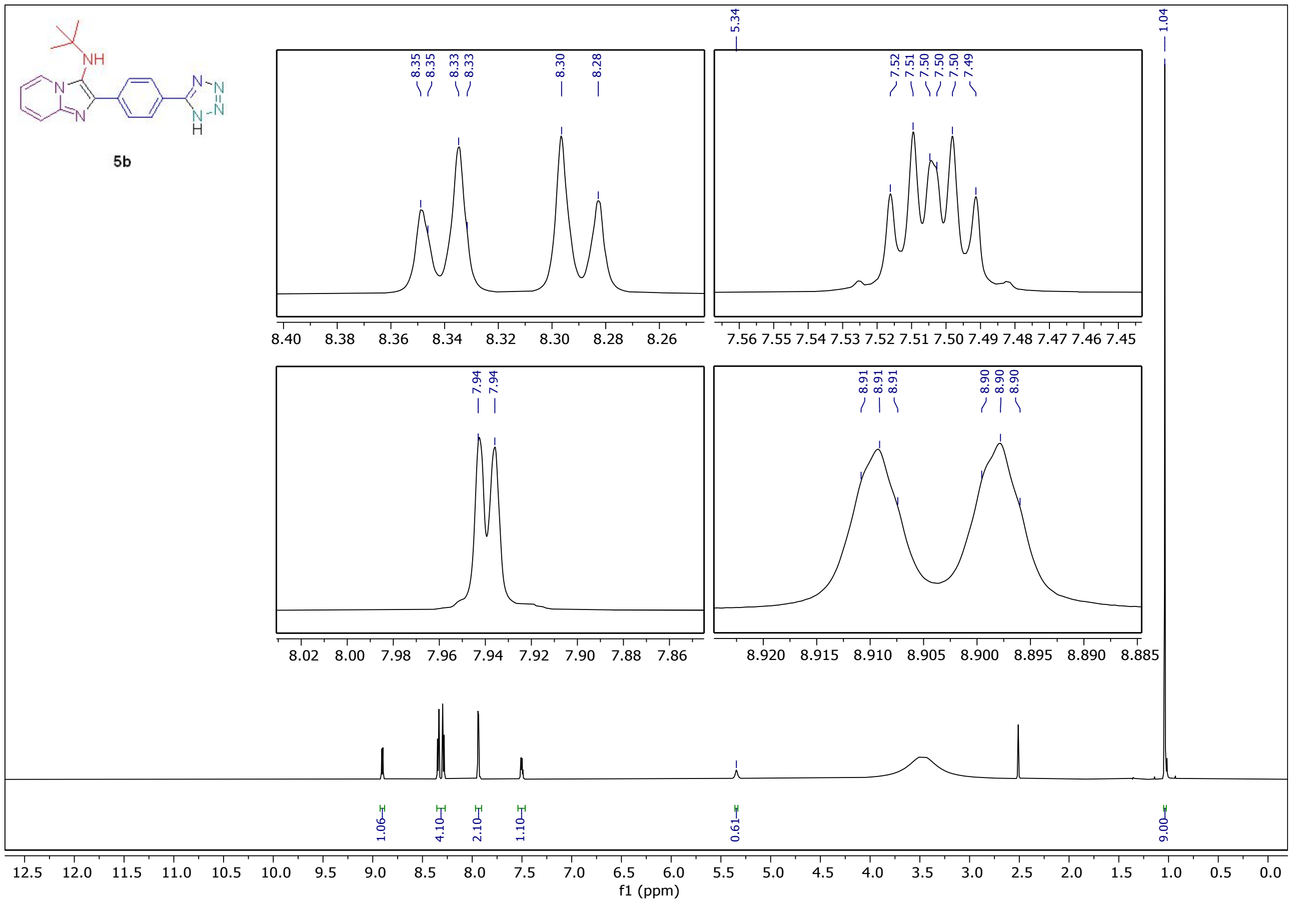

Figure S46. ^{1}H NMR spectrum (600 MHz, DMSO-d_6) of compound **5b**.

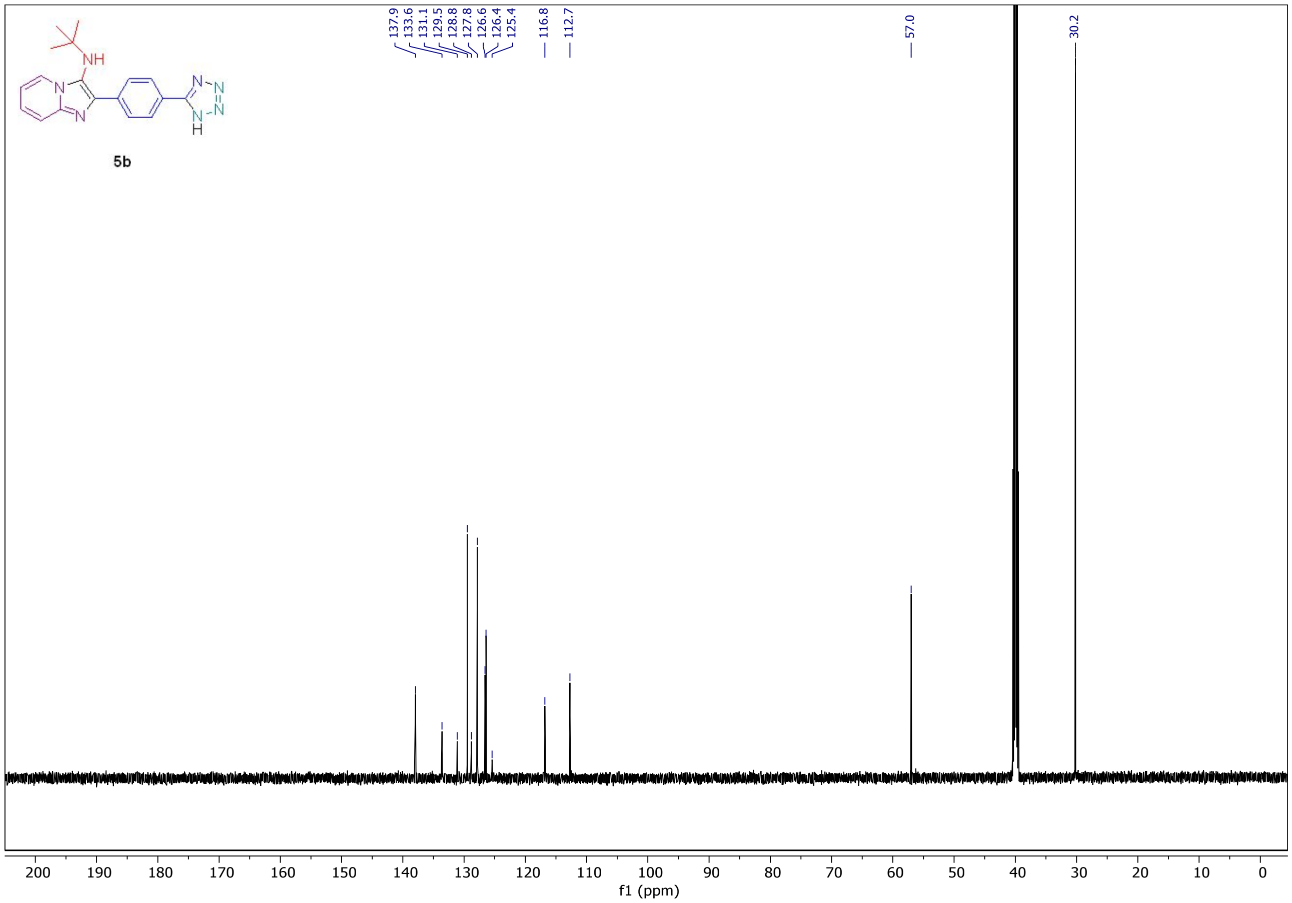

Figure S47. ^{13}C NMR spectrum (151 MHz, DMSO-d_6) of compound **5b**.

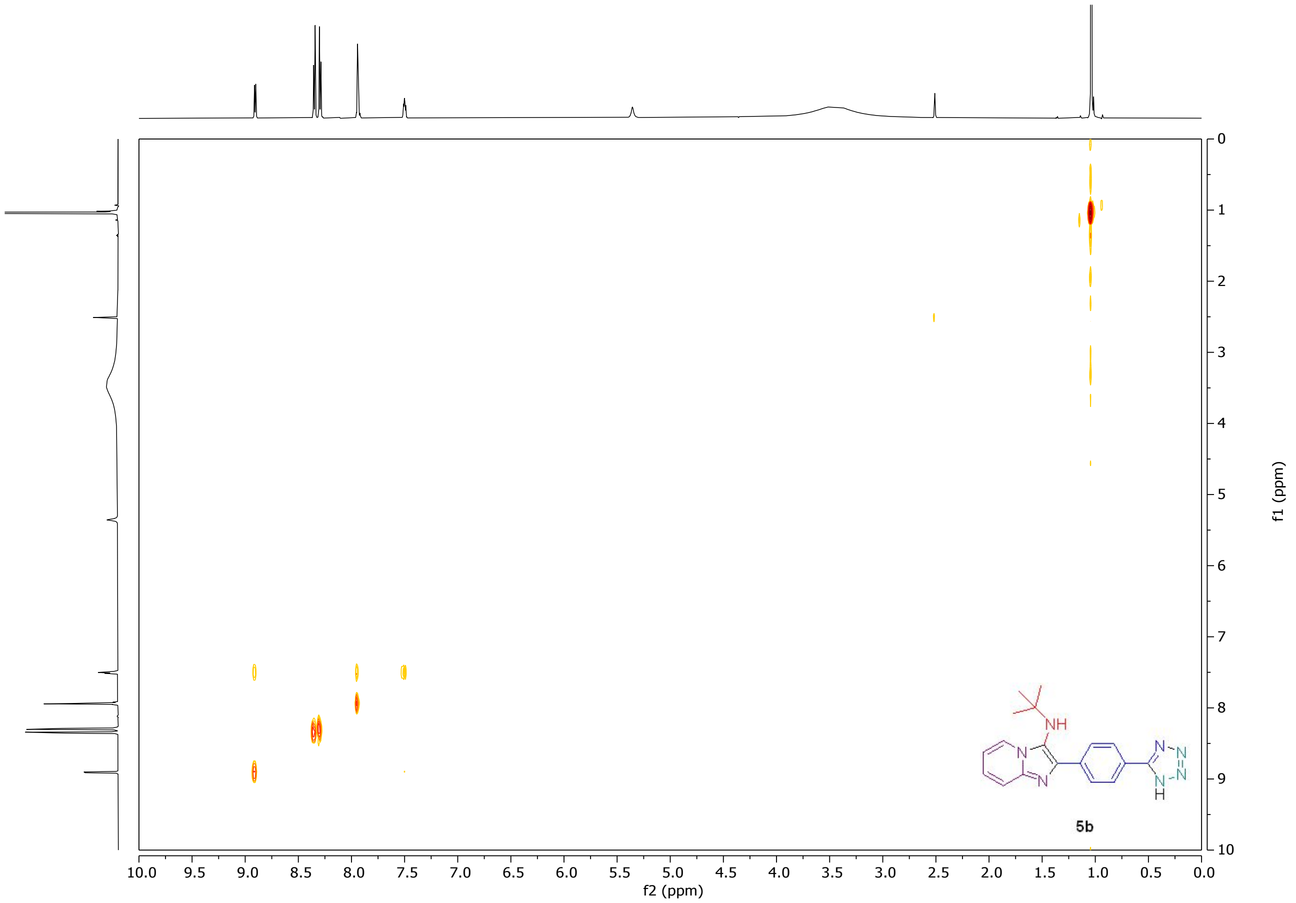

Figure S48. COSY 2D NMR spectrum (600 MHz, DMSO-d_6) of compound **5b**.

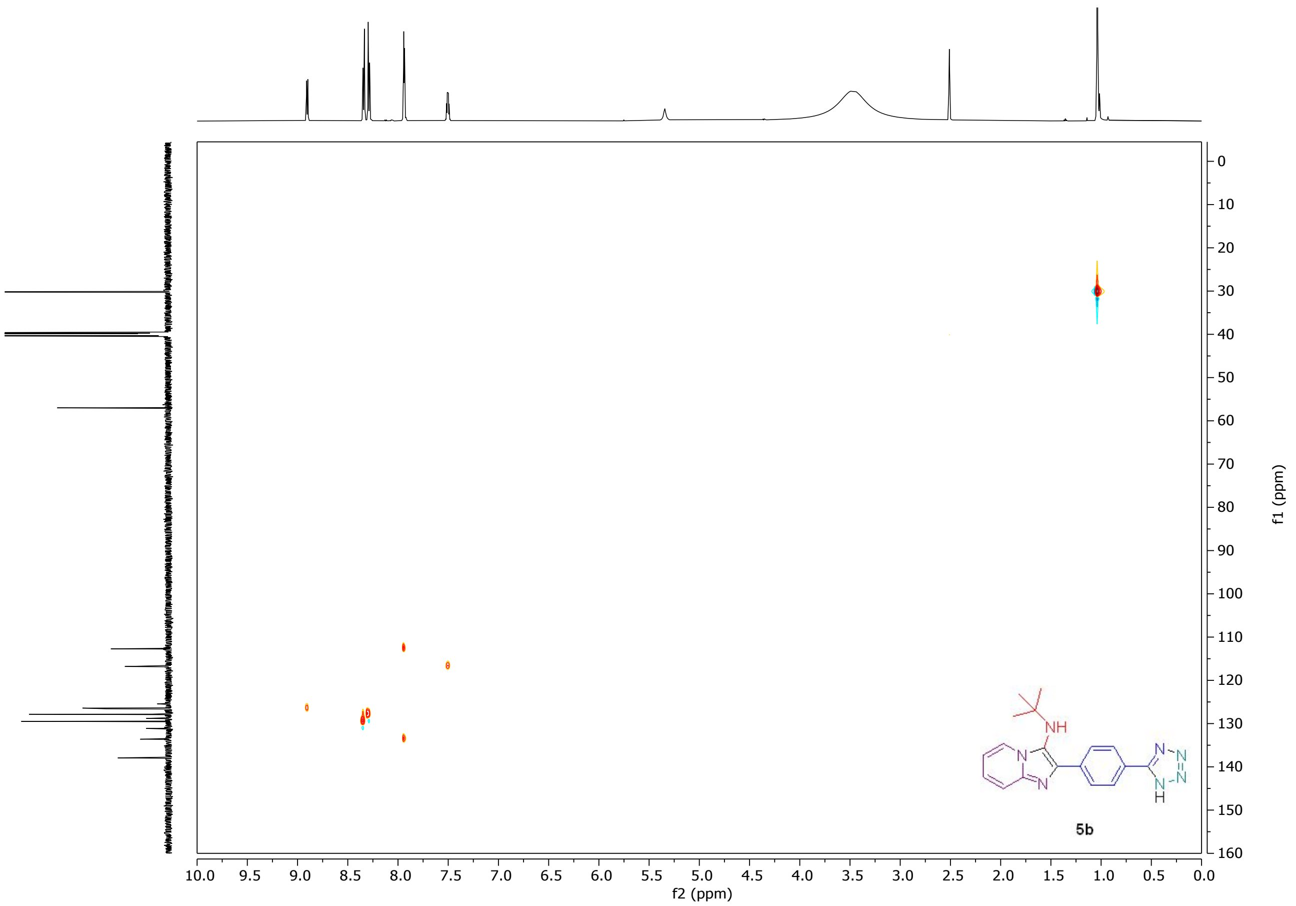

Figure S49. NOESY 2D NMR spectrum (600 MHz, DMSO-d_6) of compound **5b**.

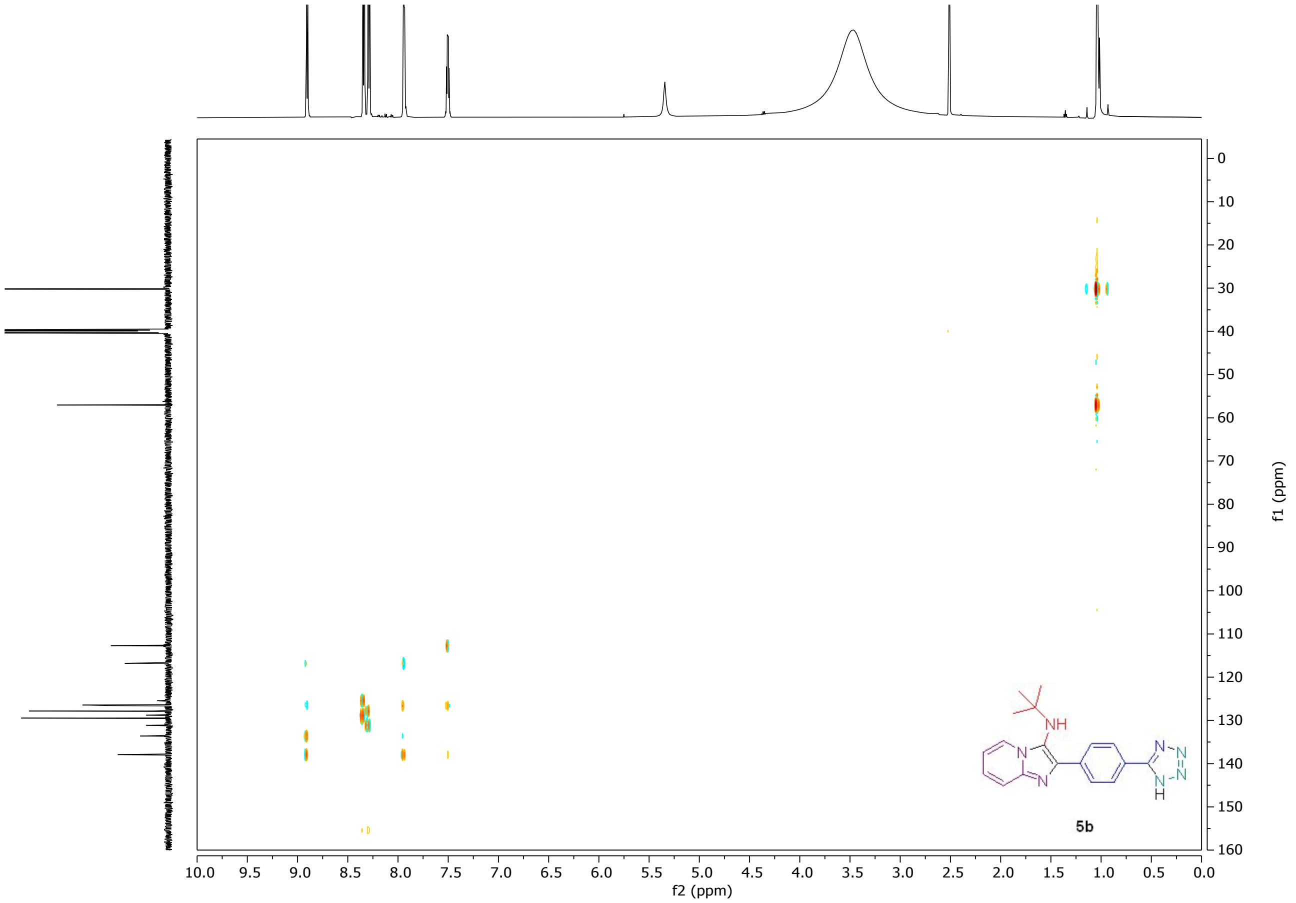

Figure S50. HSQC 2D NMR spectrum (600 MHz, DMSO-d_6) of compound **5b**.

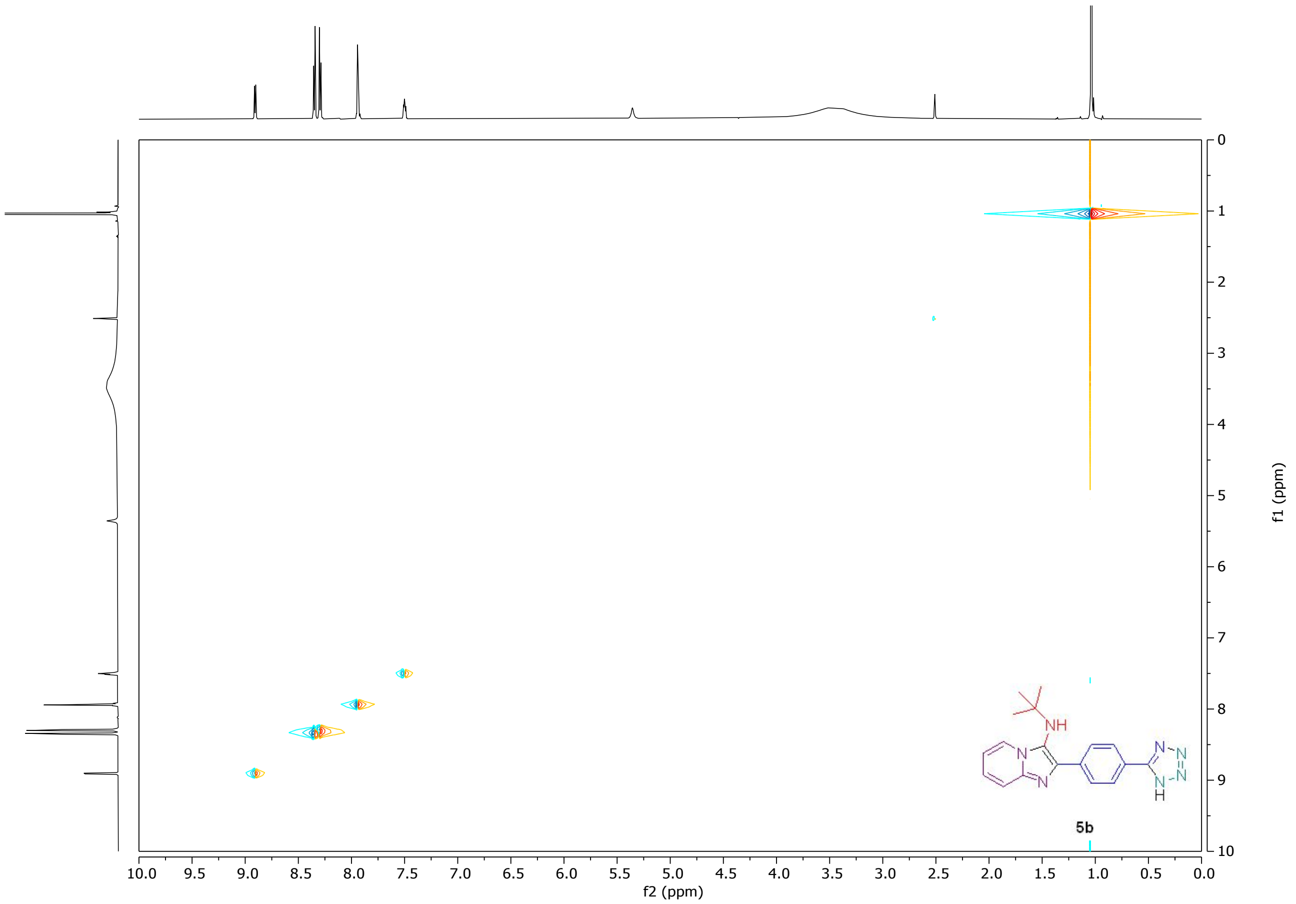

Figure S51. HMBC 2D NMR spectrum (600 MHz, DMSO-d_6) of compound **5b**.

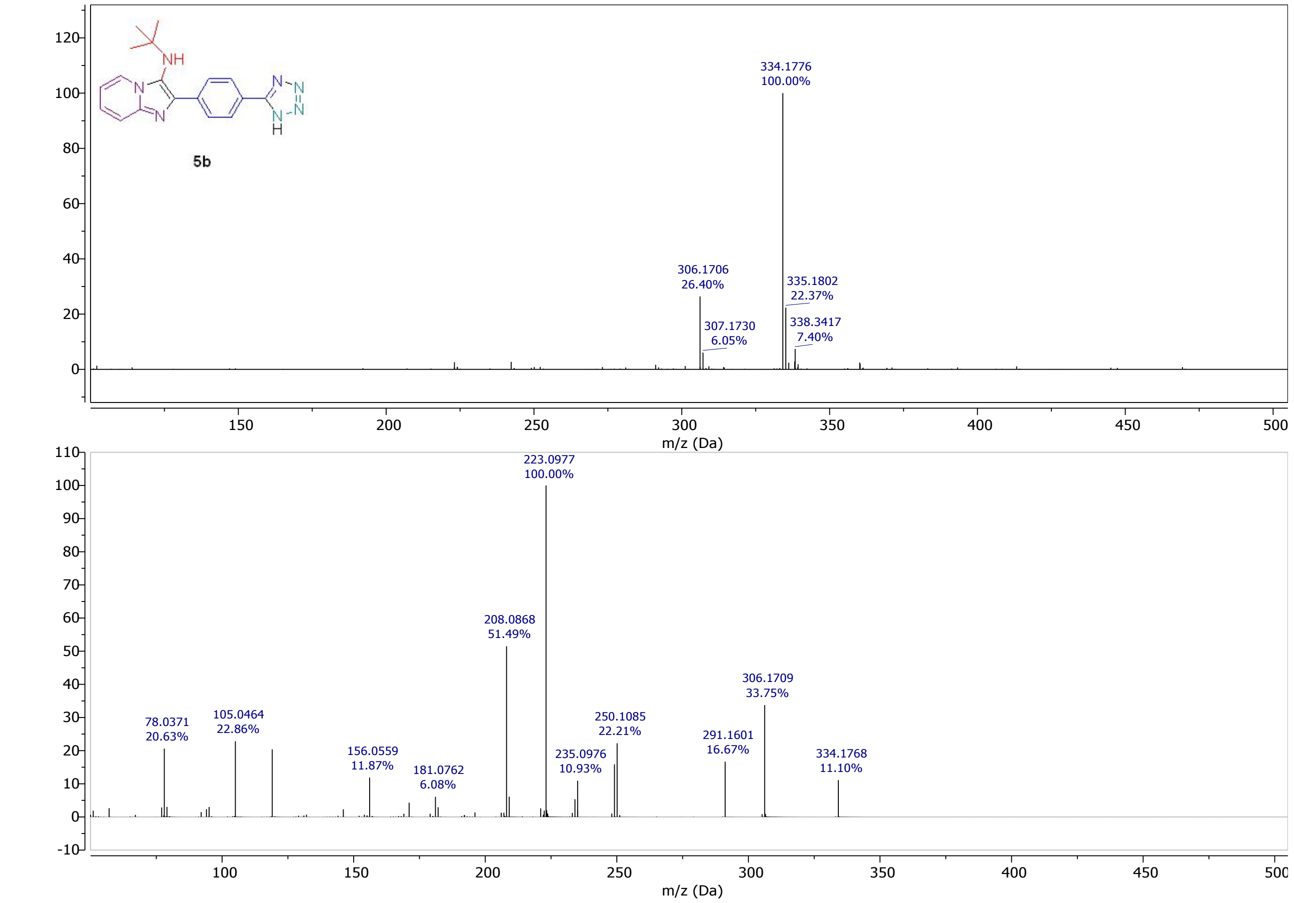

Figure S52. HRMS (ESI-QTOF) of compound **5b** and HRMS/MS for $[M+H]^+$.

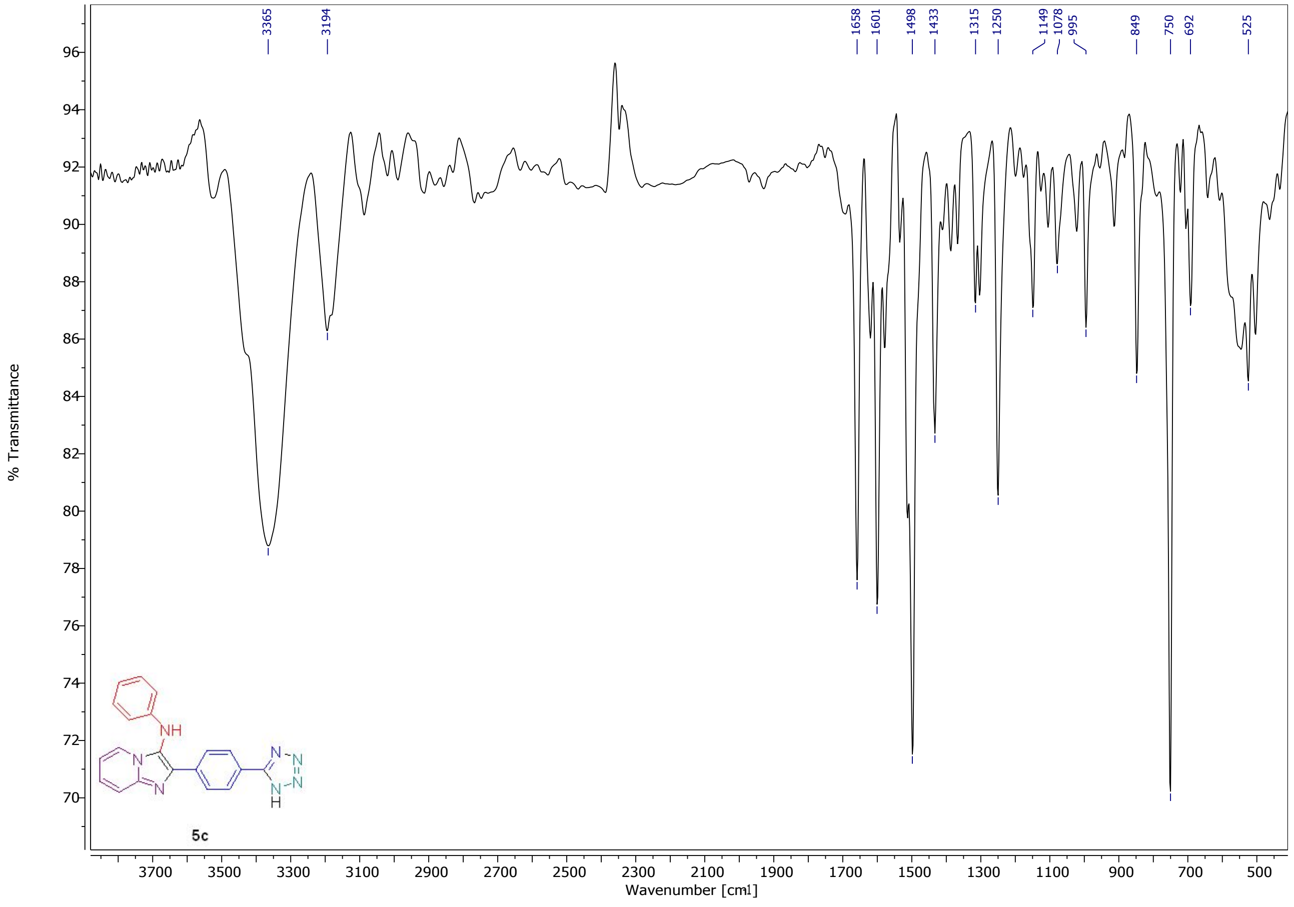

Figure S53. FT-IR (KBr) of compound **5c**.

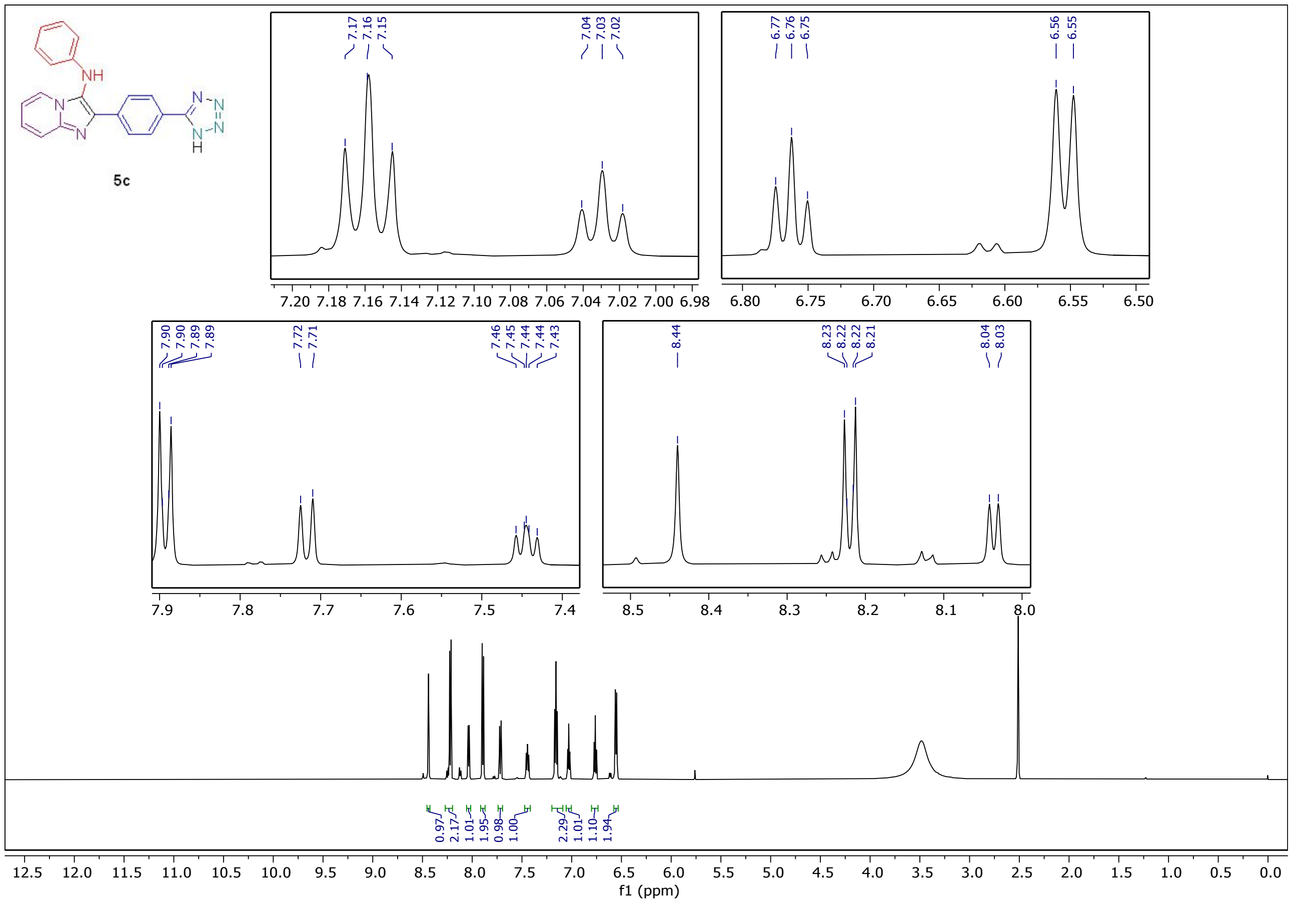

Figure S54. ^{1}H NMR spectrum (600 MHz, DMSO-d_6) of compound **5c**.

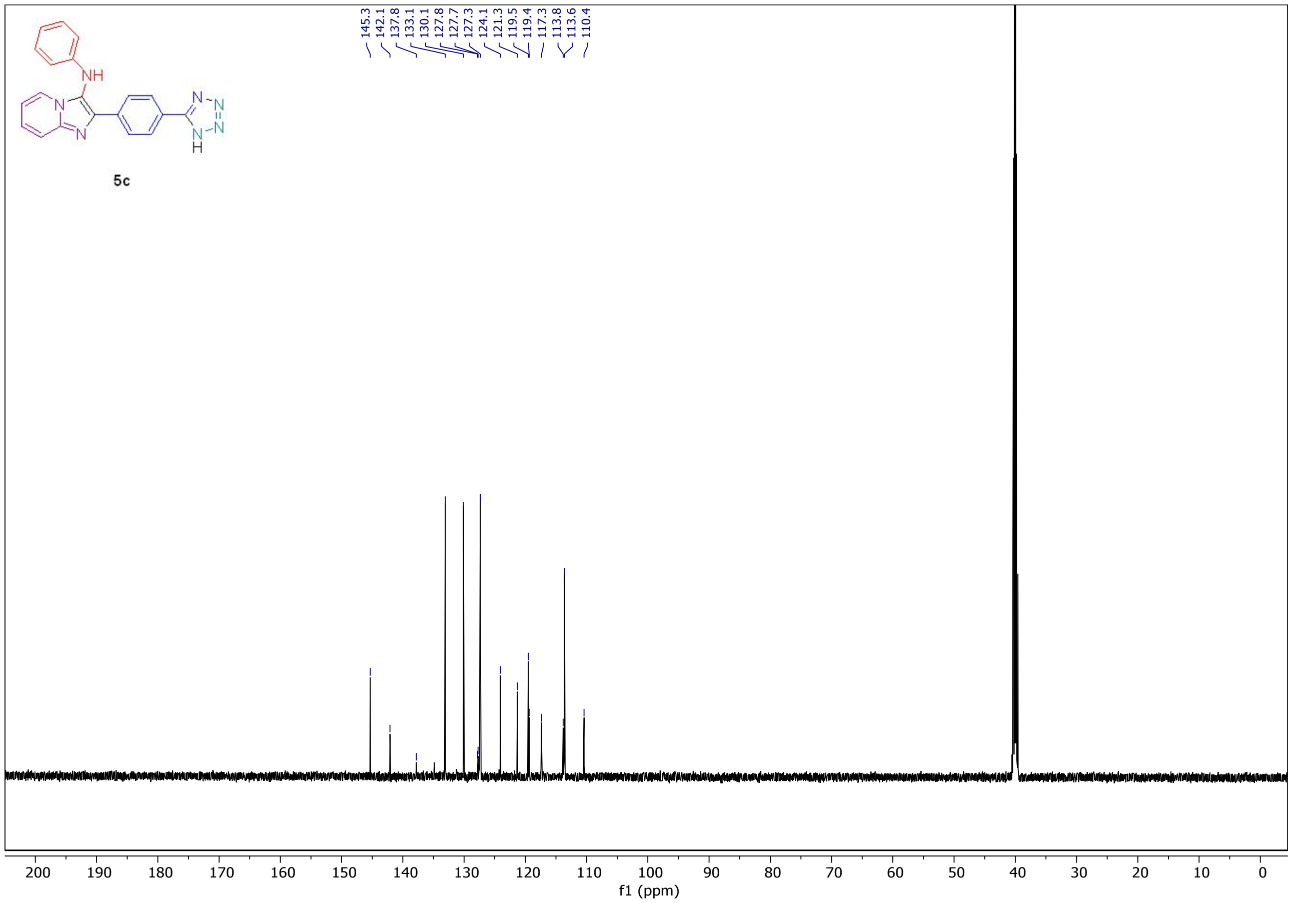

Figure S55. ^{13}C NMR spectrum (151 MHz, DMSO-d_6) of compound **5c**.

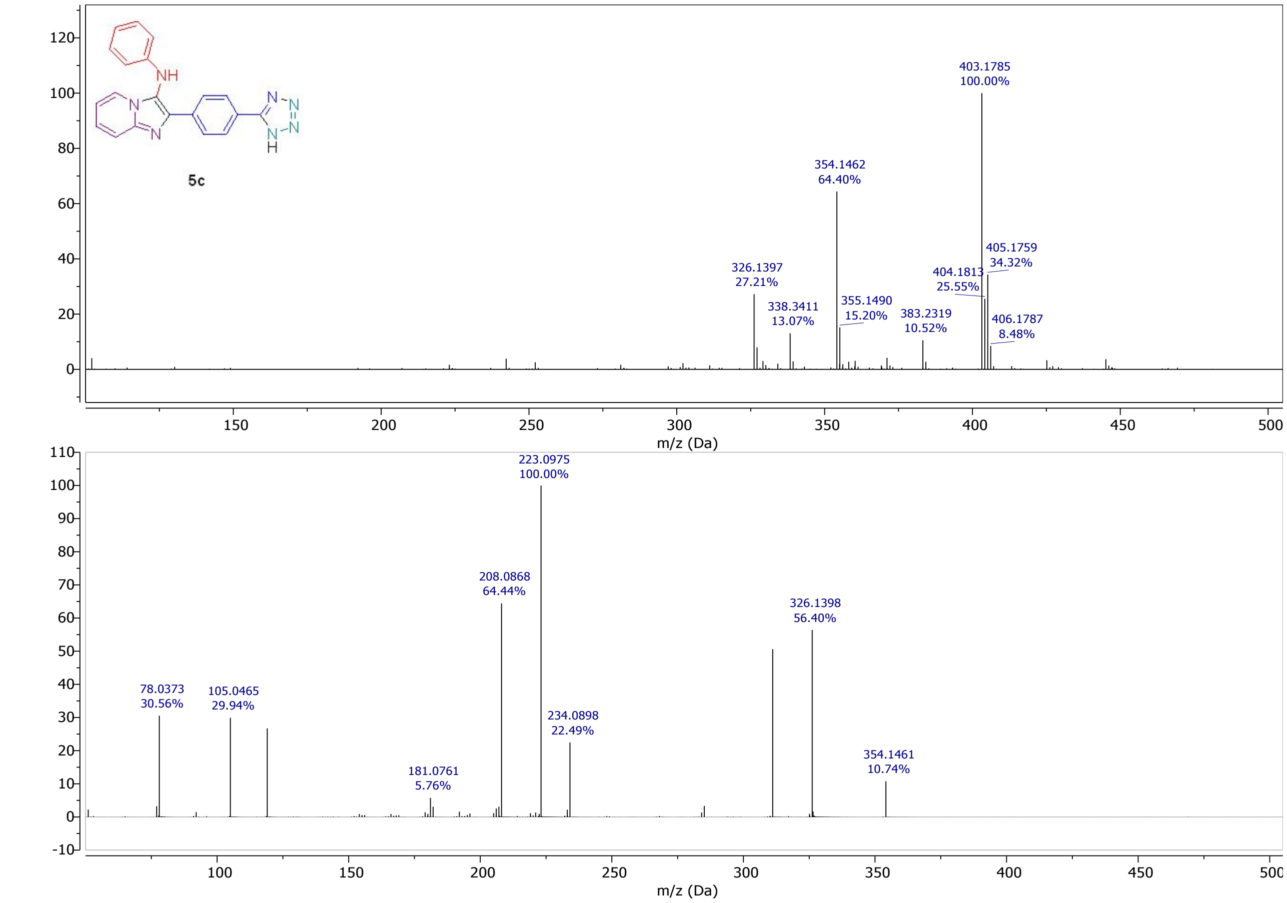

Figure S56. HRMS (ESI-QTOF) of compound **5c** and HRMS/MS for $[M+H]^+$.

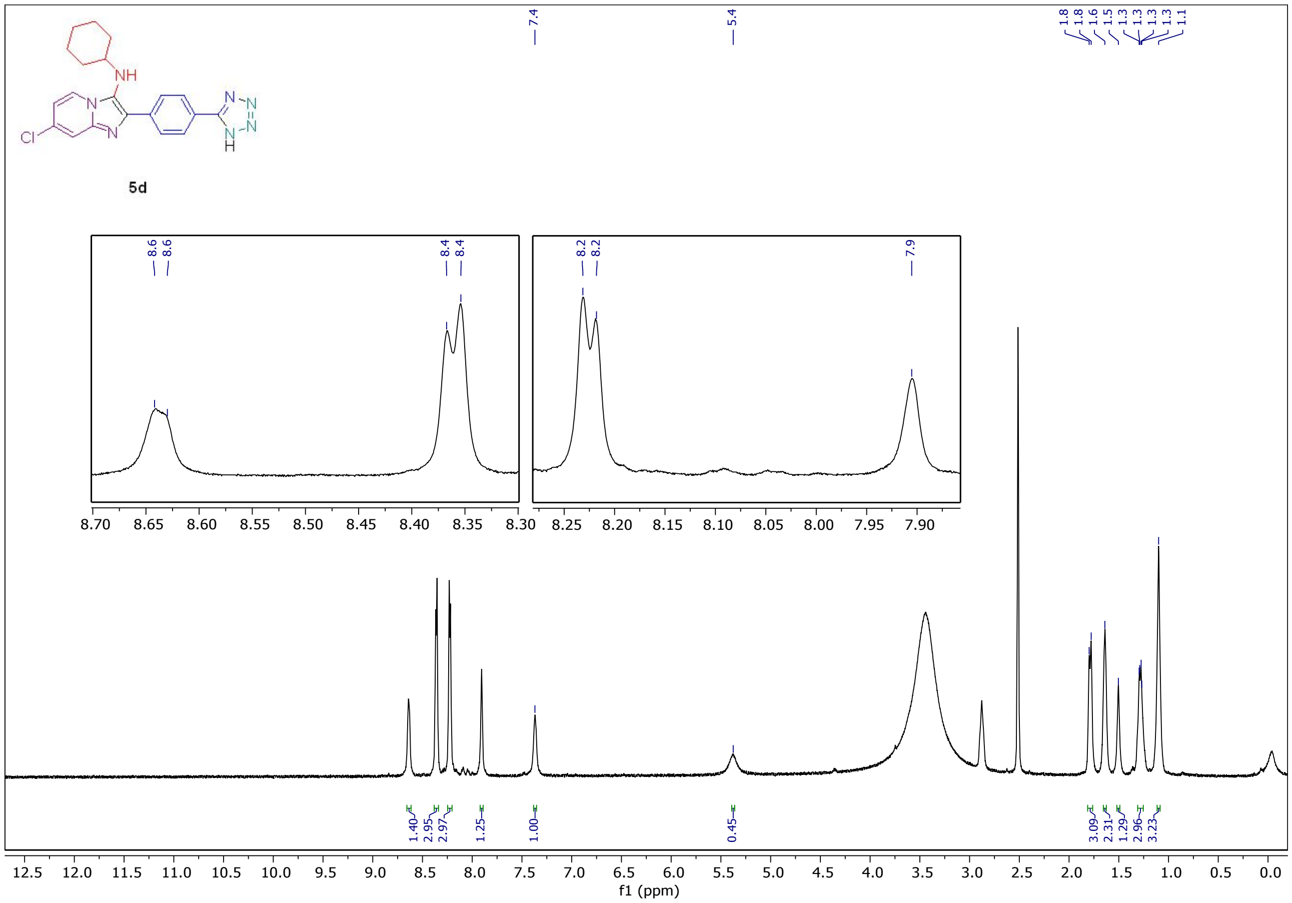

Figure S57. ^{1}H NMR spectrum (600 MHz, DMSO-d_6) of compound **5d**.

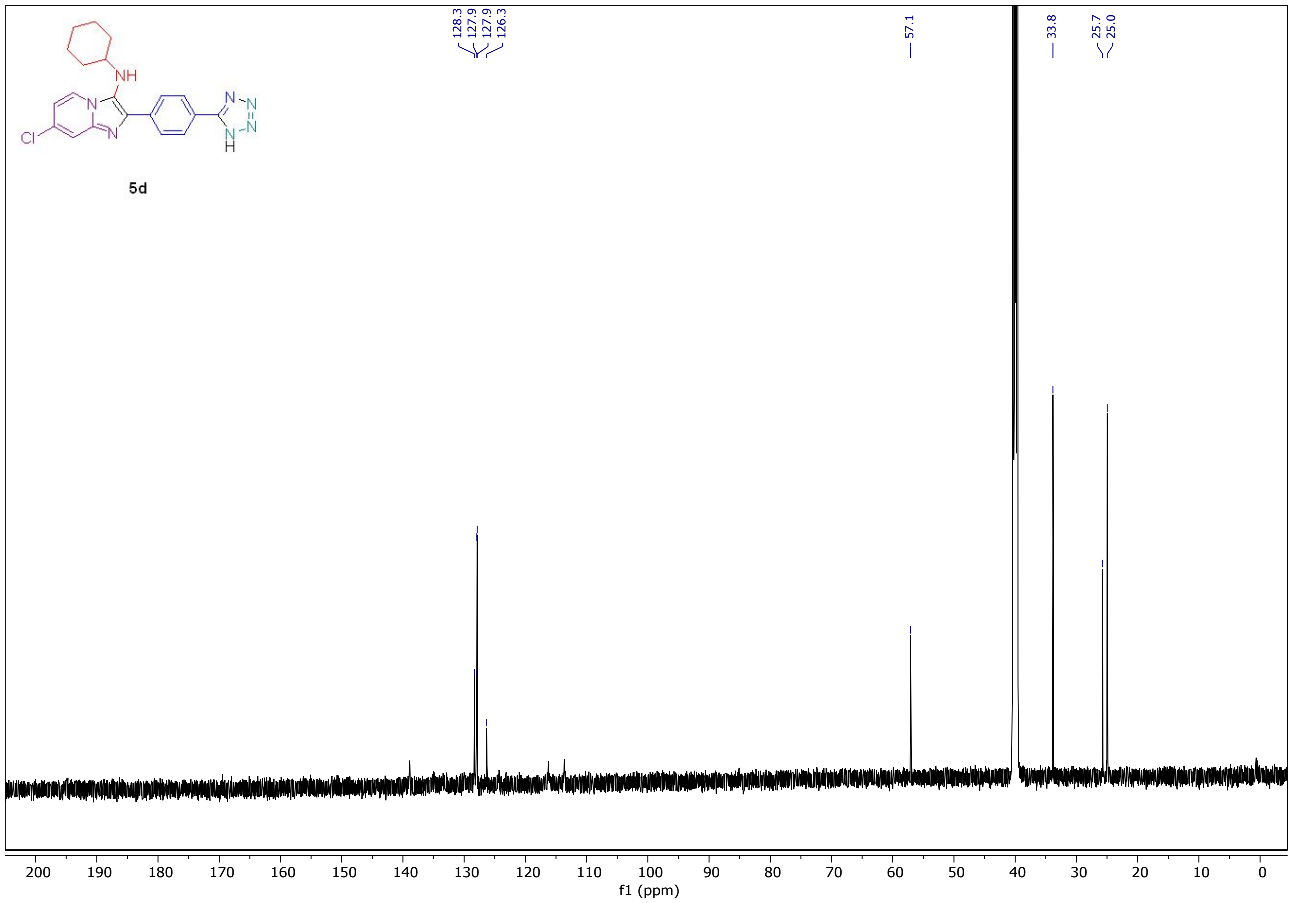

Figure S58. ^{13}C NMR spectrum (151 MHz, DMSO-d_6) of compound **5d**.

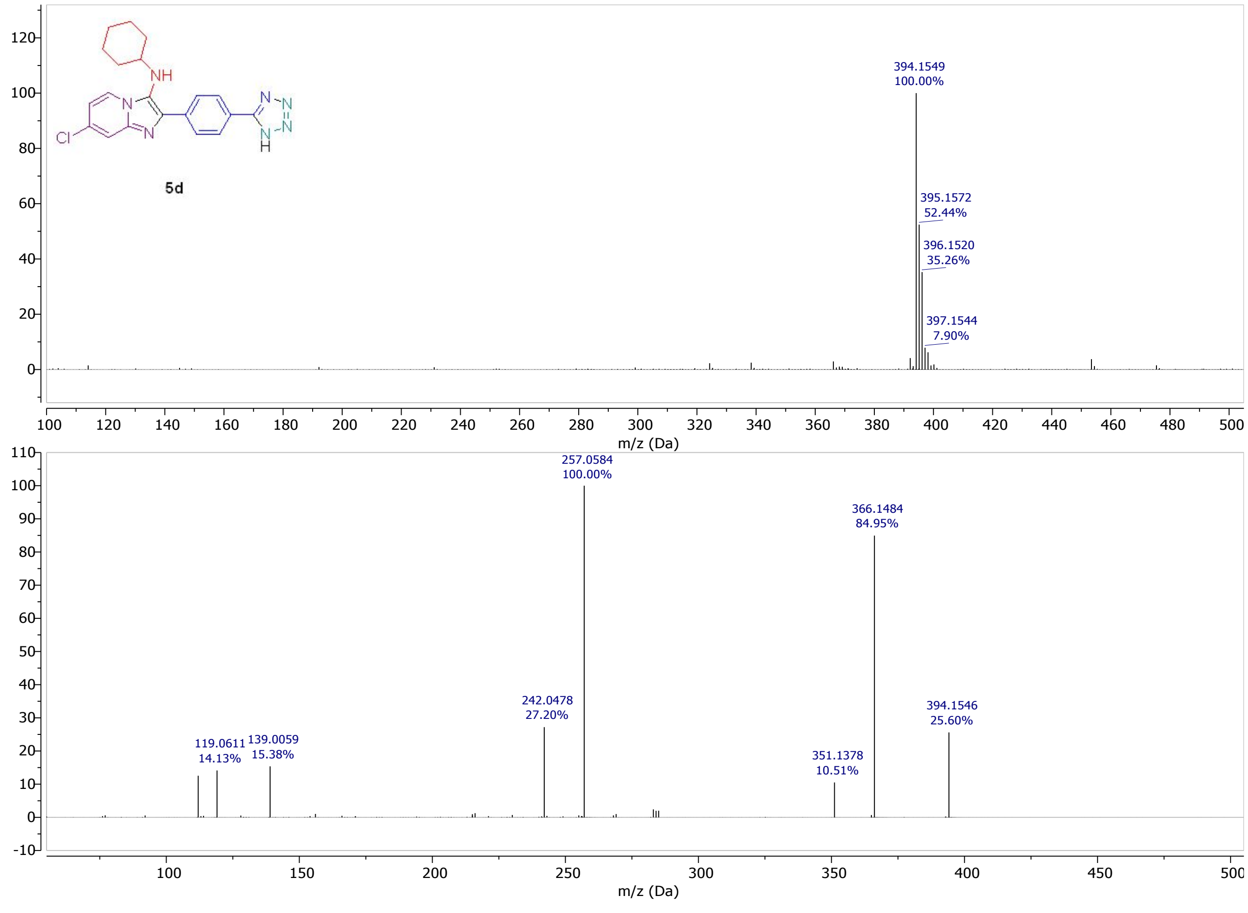

Figure S59. HRMS (ESI-QTOF) of compound **5d** and HRMS/MS for $[M+H]^+$.

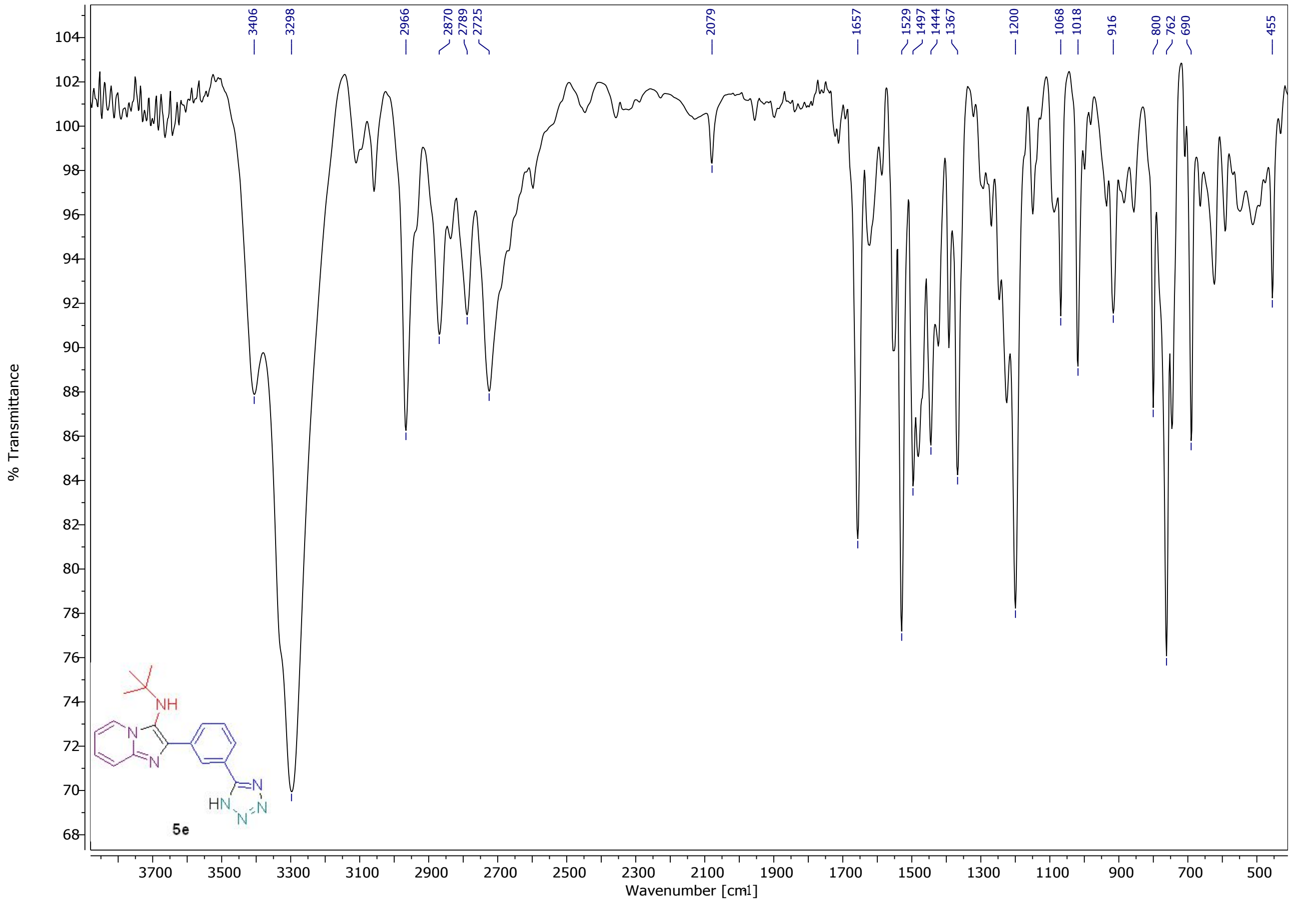

Figure S60. FT-IR (KBr) of compound **5e**.

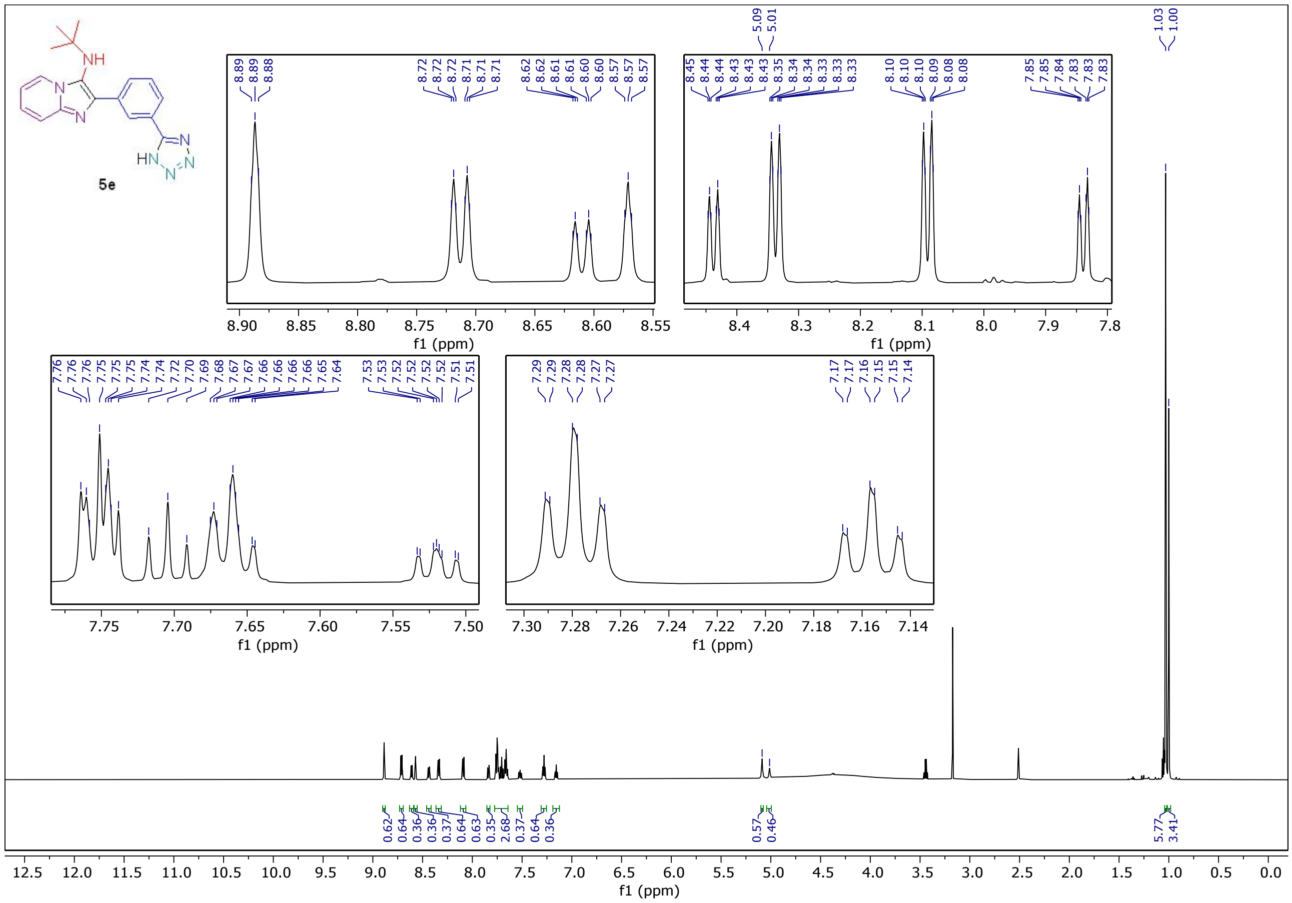

Figure S61. ^{1}H NMR spectrum (600 MHz, DMSO-d_6) of compound **5e**.

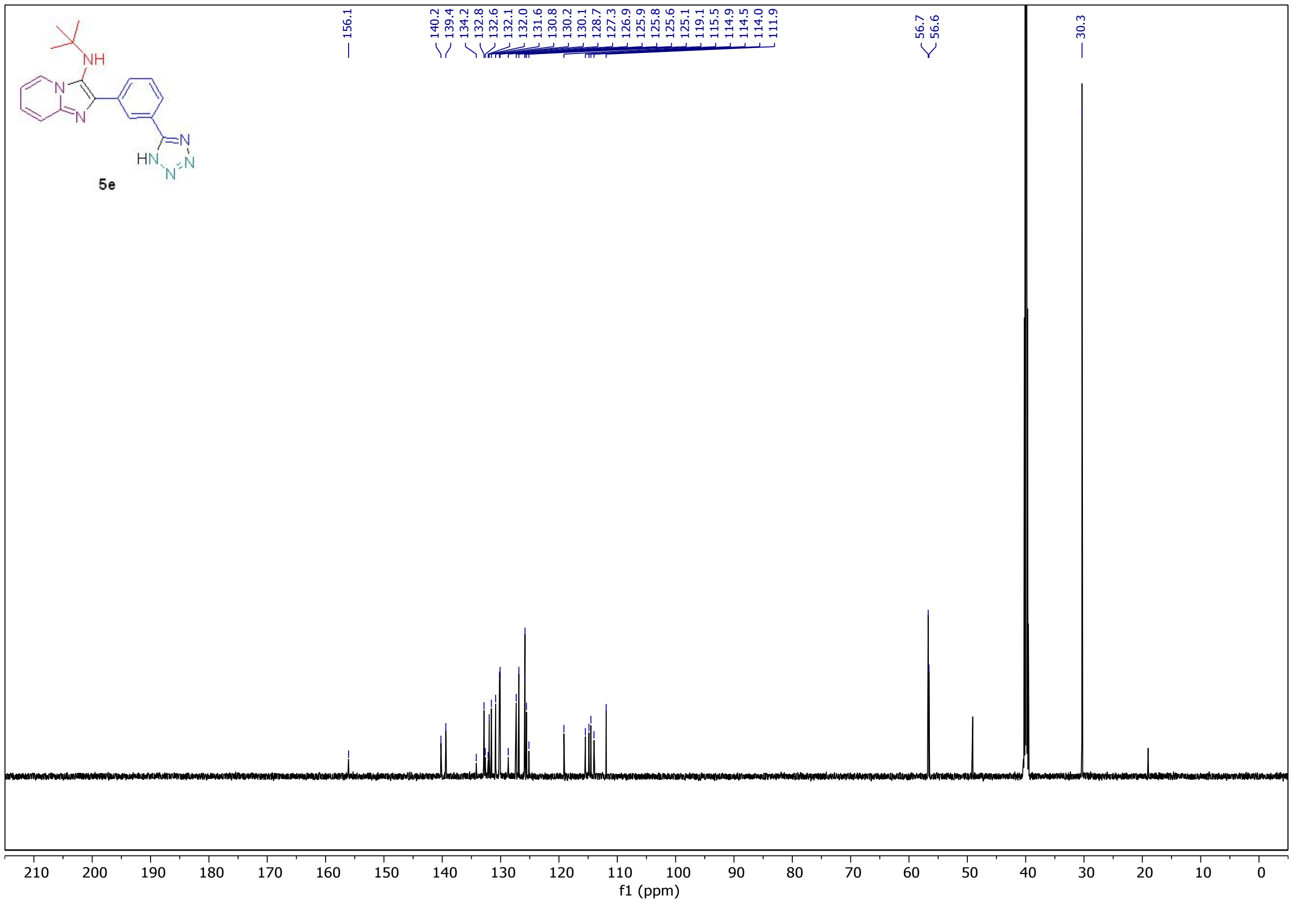

Figure S62. ^{13}C NMR spectrum (151 MHz, DMSO-d_6) of compound **5e**.

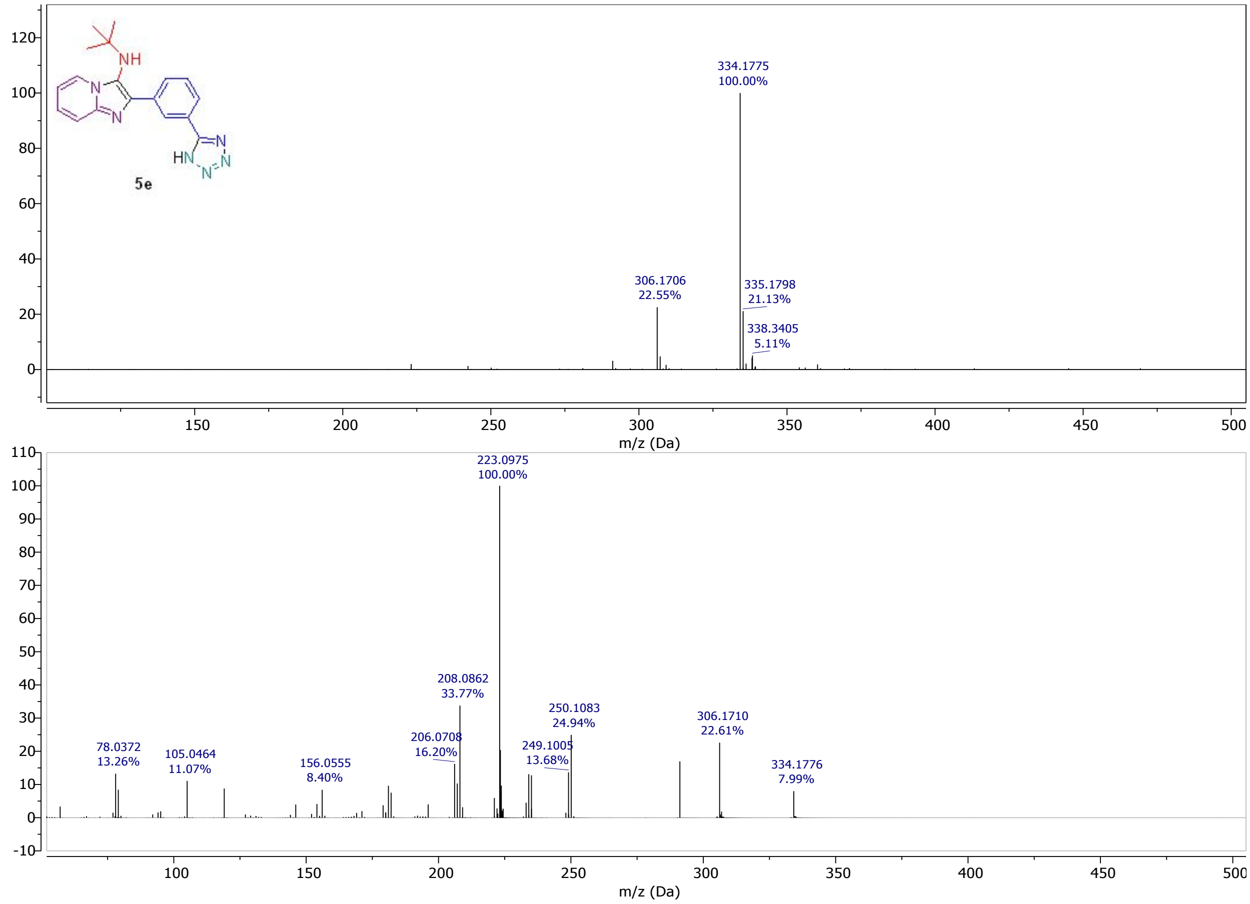

Figure S63. HRMS (ESI-QTOF) of compound **5e** and HRMS/MS for [M+H]$^+$.
